# Highly Stereo- and Enantioselective Syntheses of δ-Alkyl-Substituted (*Z*)-Homoallylic Alcohols

**DOI:** 10.1021/acs.orglett.4c04401

**Published:** 2025-01-06

**Authors:** Ming Chen

**Affiliations:** Department of Chemistry, Virginia Tech, Blacksburg, Virginia 24061, United States

## Abstract

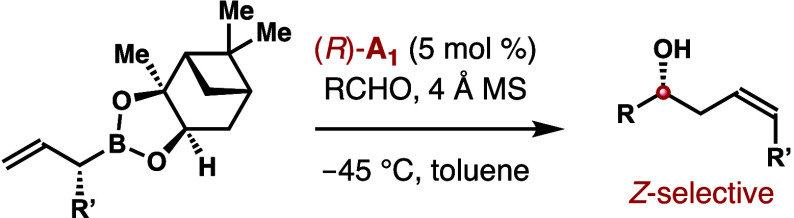

Highly stereo- and enantioselective synthesis of δ-alkyl-substituted (*Z*)-homoallylic alcohols via asymmetric allylation is developed. In the presence of a chiral phosphoric acid catalyst, allylation of aldehydes with α-substituted allylboronates provides δ-alkyl-substituted homoallylic alcohols with excellent (*Z*)-selectivities and enantioselectivities.

Oxylipins make up a class of natural products that are physiologically and pathologically significant.^1^ A key structural motif in these molecules is the δ-alkyl-substituted (*Z*)-homoallylic alcohol (Figure 1). The development of methods that allow for stereo- and enantioselective generation of such structural entities would be greatly important for the syntheses of these bioactive natural products.^2−5^

**Figure 1:**
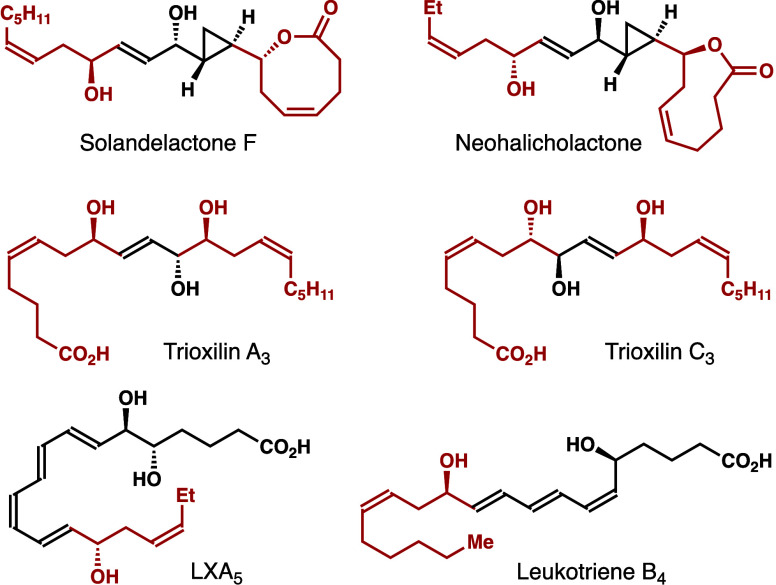
Selected natural products containing a δ-substituted (*Z*)-homoallylic alcohol structural motif.

As illustrated in Scheme 1, several approaches have been reported for the synthesis of enantioenriched δ-substituted (*Z*)-homoallylic alcohol **2**. For example, aldehyde **B** can be prepared from chiral nonracemic homoallylic alcohol **A** via alcohol protection and the oxidative cleavage of the alkene unit. (*Z*)-Selective Wittig olefination of aldehyde **B** produces enantioenriched homoallylic alcohol **2** (Scheme 1a).^2^ A second approach entails the reduction of homopropargylic alcohol **D**, which can be synthesized from allenyl organometallic species **C**.^6^ (*Z*)-Selective partial reduction of the alkyne moiety of **D** forms homoallylic alcohol **2** (Scheme 1b).^3^ Developed by the Nokami group, an allyl transfer approach can also produce homoallylic alcohol **2**.^4^ As shown in Scheme 1c, the diastereoselective allylation of isomenthone (**E**) provides intermediate **F**. Treatment of alcohol **F** with an aldehyde substrate under acidic conditions affords homoallylic alcohol **2**. More recently, a Ni-catalyzed asymmetric diene aldehyde coupling was reported by the Xiao group.^5c^ With a NHC-ligated nickel complex as the catalyst, homoallylic alcohol **2** was obtained with excellent (*Z*)-selectivity and optical purity. Despite these advances, the development of methods to generate enantioenriched homoallylic alcohol **2** remains an important objective in organic synthesis.

**Scheme 1:**
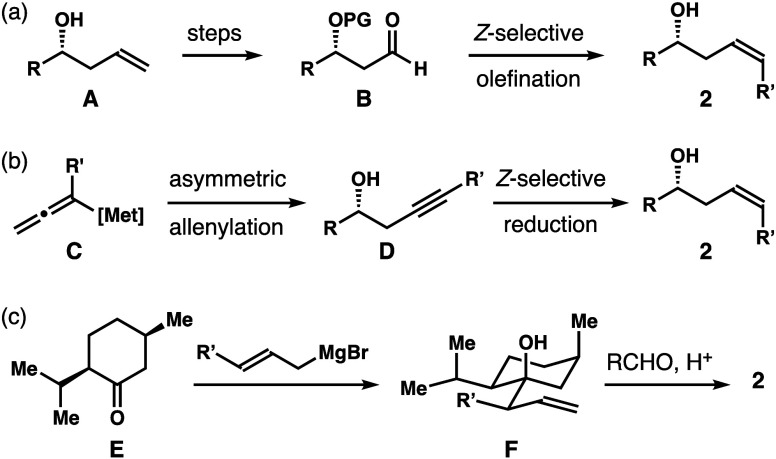
Approaches to (*Z*)-Homoallylic Alcohols

Asymmetric aldehyde allylation is one of the most adopted methods to synthesize enantioenriched homoallylic alcohols.^7^ In principle, the allylation of an aldehyde with α-substituted allylboronate [e.g., **1** (Scheme 2)] could produce (*Z*)-homoallylic alcohol **2**. However, the selectivity of such a reaction is often poor because the two competing transition states, **TS**-**1** and **TS**-**2**, are similar in energy.^8^ As a result, a mixture of (*Z*)- and (*E*)-isomers is obtained with low selectivities.

**Scheme 2:**
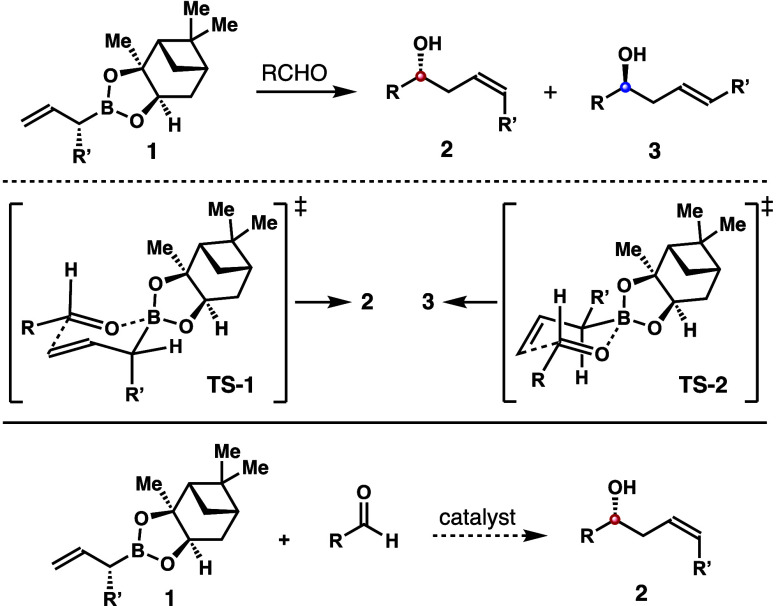
Proposed Strategy to Produce (*Z*)-Homoallylic Alcohols

Pioneered by the Antilla group, chiral phosphoric acids have been shown to catalyze asymmetric aldehyde addition with several classes of boron reagents.^9−12^ Recent studies revealed that (*Z*)-selective allylation can be achieved with achiral α-substituted allylboronates using a chiral phosphoric acid catalyst.^13^ By positioning the α-substituent in a pseudoaxial position in the reaction transition state, these reactions can produce δ-substituted homoallylic alcohols with high (*Z*)-selectivities. With our research interest in organoboron compounds,^14^ we envisaged that chiral phosphoric acid-catalyzed aldehyde allylation with enantioenriched α-substituted allylboronates **1** might be a viable strategy to generate δ-alkyl-substituted (*Z*)-homoallylic alcohols **2** (Scheme 2). We report herein the successful implementation of the approach, and homoallylic alcohols **2** were synthesized with excellent stereo- and enantioselectivities from α-substituted allylboronates **1**.

Our initial studies were focused on the syntheses of enantioenriched α-alkyl-substituted allylboronates **1**. Pioneered by the Matteson group, pinanediol is an inexpensive chiral auxiliary for the syntheses of boronic esters bearing a stereogenic center at the α-position.^15^Scheme 3 summarizes asymmetric syntheses of α-substituted allylboronates **1** under the Matteson homologation conditions, and a variety of α-alkyl-substituted allylboronates **1** were synthesized from alkyl boronic esters **I**.

**Scheme 3:**
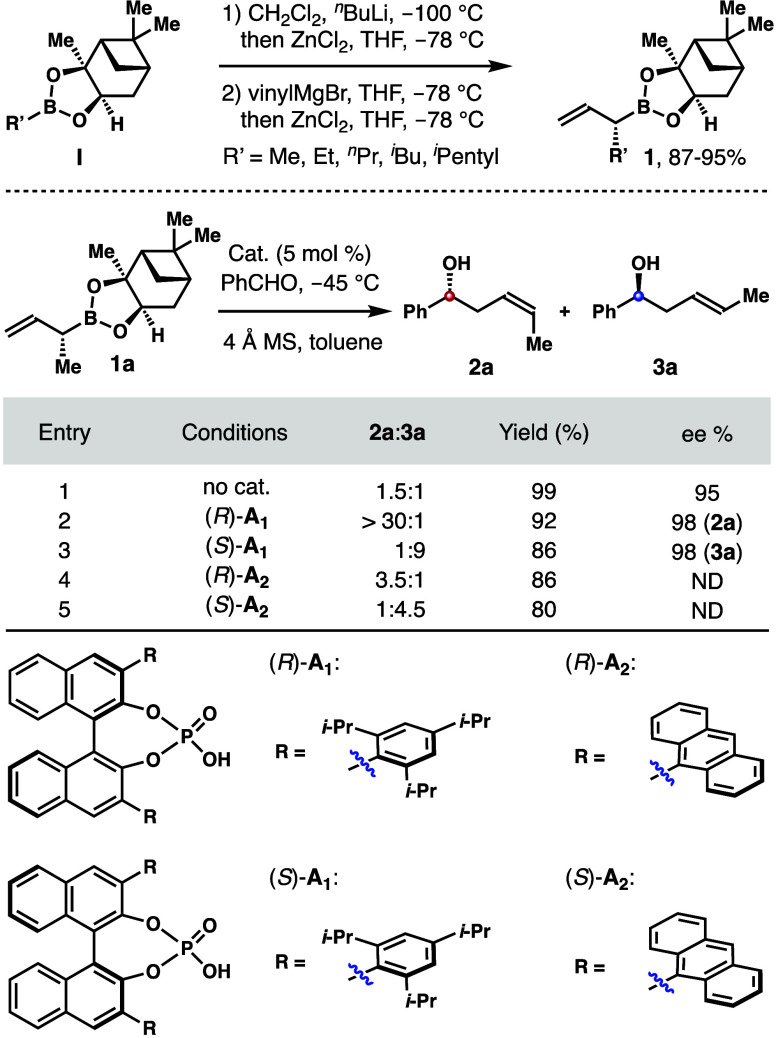
Syntheses of α-Alkyl-Substituted Allylboronates **1** and Evaluation of the Conditions for Stereoselective Allylation with Boronate **1a**– Reaction conditions: allylboronate **1a** (0.12 mmol, 1.2 equiv), PhCHO (0.1 mmol, 1.0 equiv), phosphoric acid (5 mol %), 4 Å MS (50 mg), toluene, −45 °C. The *Z*/*E* ratios were determined by ^1^H NMR analyses of the crude reaction products. Yields of isolated products are listed. Enantioselectivities were determined by Mosher ester analyses.

With these boron reagents in hand, allylation studies with boronate **1a** were conducted first. As shown in Scheme 3, the reaction of benzaldehyde without any catalyst gave a 1.5:1 mixture of alcohols **2a** and **3a**, slightly favoring (*Z*)-isomer **2a** (entry 1). When 5 mol % chiral phosphoric acid (*R*)-**A**_**1**_ was used, the reaction afforded **2a** as the only detectable product (*Z*:*E* > 30:1) in 92% yield with 98% ee (entry 2).^16^ When enantiomeric catalyst (*S*)-**A**_**1**_ was employed, a 1:9 mixture of **2a** and **3a** was obtained in a combined 86% yield, with (*E*)-isomer **3a** (98% ee) as the major product (entry 3). Therefore, by using different enantiomers of the acid catalyst, either the (*Z*)-isomer or the (*E*)-isomer can be obtained selectively from the same boron reagent **1a**. The reactions with acid (*R*)-**A**_**2**_ or (S)-**A**_**2**_ as the catalyst were also examined. However, the (*E*/*Z*)-selectivities of these reactions were moderate (entries 4 and 5).

Scheme 4 summarizes the scope of phosphoric acid (*R*)-**A**_**1**_-catalyzed asymmetric allylation with boronate **1a**. A broad range of aldehydes participated in the reactions to deliver δ-methyl-substituted homoallylic alcohols **2** in high yields with excellent (*Z*)-selectivities and enantioselectivities. For instance, reactions of *para*-substituted aromatic aldehydes with **1a** gave products **2b**–**e** in 79–91% yields with 97–98% ee. Aromatic aldehydes with other substitution patterns also reacted to form alcohols **2f**–**i** in 83–94% yields with 98–99% ee. The reaction proceeded smoothly with α,β-unsaturated aldehydes, affording products **2j**–**l** in 70–90% yields with 97–99% ee. Aldehydes with a heterocycle reacted with **1a** to generate alcohols **2m**–**o** in 84–93% yields with 98–99% ee. Reactions with several aliphatic aldehydes also worked well to furnish products **2p**–**r** in 72–84% yields with 98–99% ee. In all cases, (*Z*)-isomers were obtained with excellent selectivities (>30:1).

**Scheme 4:**
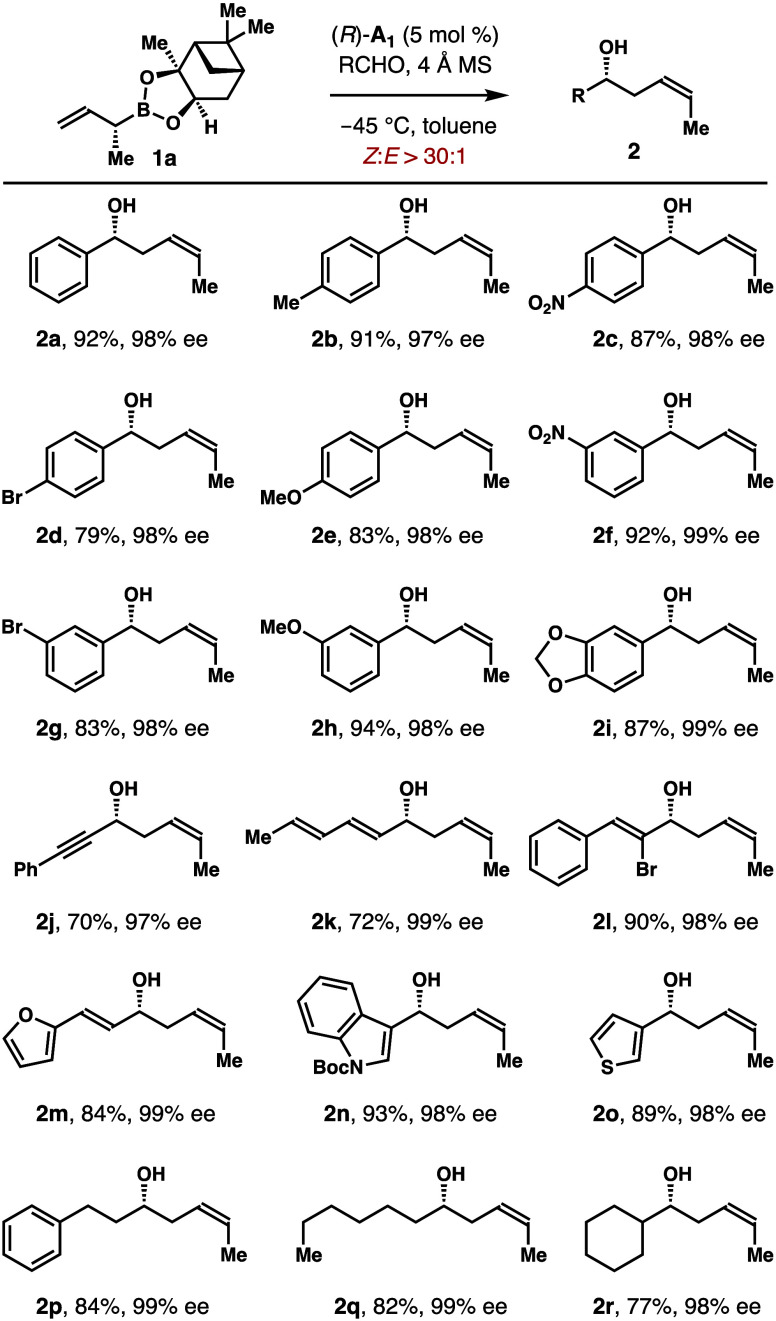
Scope of the Asymmetric Allylboration with Boronate **1a** Catalyzed by Phosphoric Acid (*R*)-**A_1_**– Reaction conditions: allylboronate **1a** (0.12 mmol, 1.2 equiv), aldehyde (0.1 mmol, 1.0 equiv), phosphoric acid (*R*)-**A**_**1**_ (5 mol %), 4 Å molecular sieves (50 mg), toluene (0.3 mL), −45 °C, 48 h. Enantioselectivities of **2** were determined by Mosher ester analyses. The *Z*/*E* ratios were determined by ^1^H NMR analyses of the crude reaction products. Yields of isolated products are listed.

Reactions of reagent **1a** with two enantioenriched aldehydes were examined. As shown in Scheme 5, allylation of aldehyde **4** with **1a** using (*R*)-**A**_**1**_ as the catalyst afforded product **2s** in 91% yield with >30:1 (*Z*)-selectivity and diastereoselectivity. Under identical conditions, the reaction with enantiomeric aldehyde **5** gave product **2t** in 87% yield, again with excellent (*Z*)-selectivity and diastereoselectivity. The results showed that these two reactions proceeded with perfect catalyst control.

**Scheme 5:**
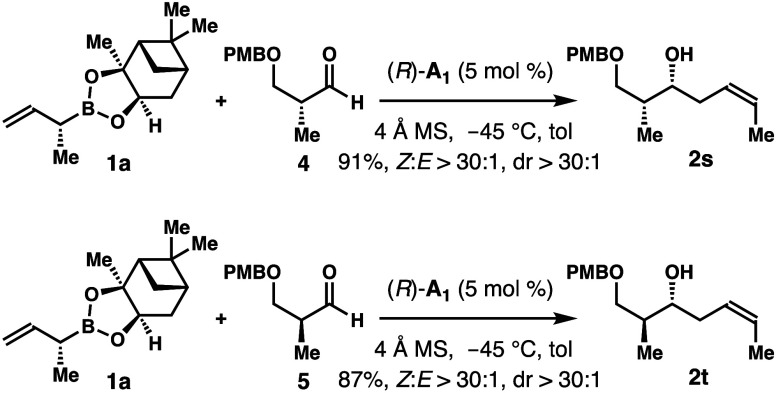
Allylboration with Enantioenriched Aldehydes

Reactions with allylboronates bearing different alkyl groups at the α-position were explored next, and the results are summarized in Scheme 6. Several alkyl groups, including ethyl, *n*-propyl, *i*-butyl, and *i*-pentyl groups, are tolerated. The reactions of alkyl-substituted allylboronates **1b**–**e** with a few representative aldehydes provided homoallylic alcohols **6a**–**l** in 73–95% yields with 94–99% ee and >30:1 (*Z*)-selectivities.

**Scheme 6:**
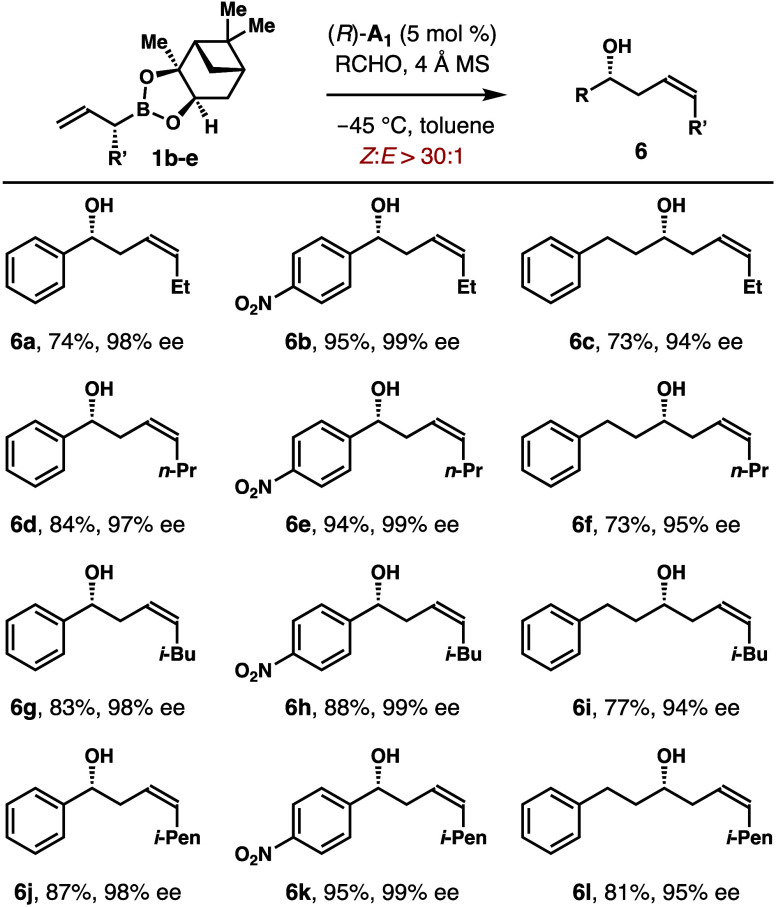
Scope of Phosphoric Acid (*R*)-**A_1_**-Catalyzed Allylboration with Allylboronates **1b–e**– Reaction conditions: allylboronate **1** (0.12 mmol, 1.2 equiv), aldehyde (0.1 mmol, 1.0 equiv), phosphoric acid (*R*)-**A**_**1**_ (5 mol %), 4 Å molecular sieves (50 mg), toluene (0.3 mL), −45 °C, 48 h. Enantioselectivities of **6** were determined by Mosher ester analyses. The *Z*/*E* ratios were determined by ^1^H NMR analyses of the crude reaction products. Yields of isolated products are listed.

The method was applied to the fragment synthesis of leukotriene B_4_. As shown in Scheme 7, the reaction of aldehyde **7** with *n*-pentyl-substituted allylboronate **1f** in the presence of catalyst (*R*)-**A**_**1**_ gave homoallylic alcohol **8** in 73% yield with excellent (*Z*)-selectivity and enantioselectivity. The reaction with aldehyde **9** under identical reaction conditions generated product **10** in 70% yield with >30:1 (*Z*)-selectivity and 95% ee. Either alcohol **8** or **10** could serve as an intermediate for the synthesis of leukotriene B_4_.

**Scheme 7:**
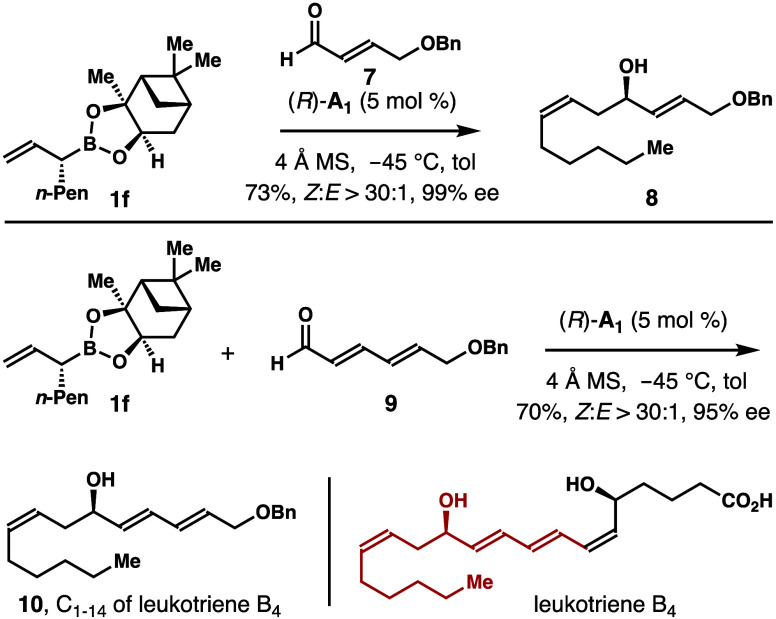
Fragment Synthesis of Leukotriene B_4_

The origin of the observed (*Z*)-selectivity is rationalized by the transition state analyses shown in Scheme 8. On the basis of the Goodman model,^12^ the addition of reagent **1a** to benzaldehyde with acid (*R*)-**A**_**1**_ as the catalyst has two competing transition states, where the acid catalyst forms two hydrogen bonds with the boronate–aldehyde complex. In transition state **TS**-**3** that forms (*Z*)-adduct **2a**, the α-methyl group adopts the pseudoaxial position, and the pinanediol on boron is positioned away from the aryl groups of the catalyst. By contrast, in transition state **TS**-**4** that forms (*E*)-adduct **3a**, the pinanediol group develops steric interactions with the acid catalyst. Moreover, the gauche interactions between the pseudoequatorially positioned methyl group and the pinanediol group on boron further destabilize **TS**-**4**. Therefore, transition state **TS**-**3** is strongly favored. When acid (*S*)-**A**_**1**_ was used as the catalyst for the reaction with the same reagent **1a**, transition state **TS**-**5** is favored, as it minimizes the steric repulsion between the pinanediol group and the acid catalyst. However, it also suffers the nonbonding gauche interactions between the α-methyl group and the pinanediol group on boron. As a result, (*E*)-isomer **3a** is formed selectively, albeit with diminished (*E*)-selectivity.

**Scheme 8:**
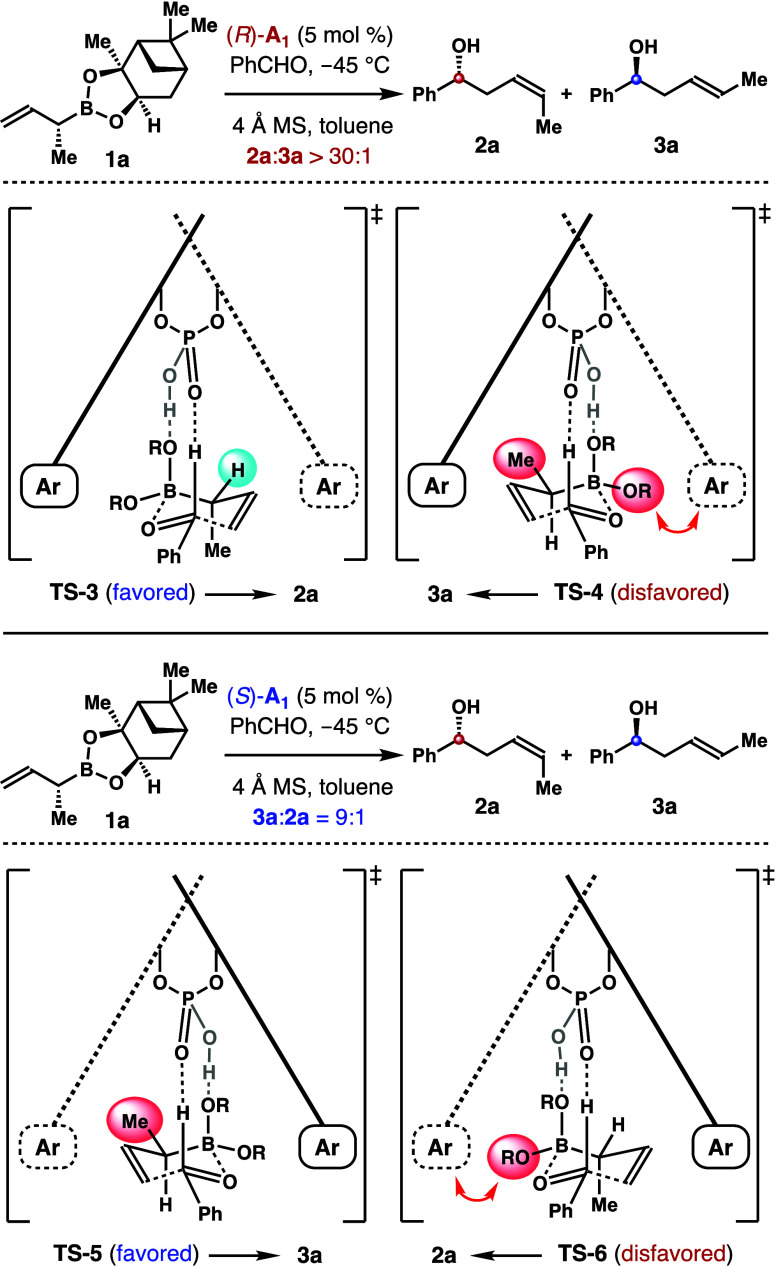
Transition State Analyses

In summary, we developed highly stereo- and enantioselective syntheses of δ-alkyl-substituted (*Z*)-homoallylic alcohols from α-substituted allylboronates. In the presence of a catalytic amount of chiral phosphoric acid, the addition of α-substituted allylboron reagents to aldehydes provided δ-alkyl-substituted homoallylic alcohols with excellent (*Z*)-selectivities and enantioselectivities. It is anticipated that the reaction should be highly valuable for the syntheses of natural products that contain a (*Z*)-homoallylic alcohol unit as depicted in Figure 1. Synthetic applications are currently underway.

## Data Availability

The data underlying this study are available in the published article and its Supporting Information.

## Supporting Information Available

Experimental procedures and spectra for all new compounds (PDF)
