# Supplementary material for: Highly Stereo- and Enantioselective Syntheses of δ-Alkyl-Substituted (Z)-Homoallylic Alcohols
Source: Org Lett. 2025 Jan 6;27(2):612–7. doi: 10.1021/acs.orglett.4c04401 (PMC11744790; doi:10.1021/acs.orglett.4c04401)
Supplement: Supplementary file 1 — ol4c04401_si_001.pdf [file ol4c04401_si_001.pdf]

Highly Stereo- and Enantioselective Syntheses of δ-Alkyl-substituted (*Z*)-Homoallylic Alcohols

Ming Chen*

Department of Chemistry, Virginia Tech

E-mail: mzc0102@vt.edu

Supporting Information: Experimental Procedures, Tabulated Spectroscopic Data, ^{1}H and ^{13}C Spectra of New Compounds

General Experimental Details. All reaction solvents were purified before use. Tetrahedrofuran and toluene were purified by passing through a column composed of activated A-1 alumina. Unless indicated otherwise, all reactions were conducted under argon atmosphere using flame- or oven-dried (140 °C) glassware. The term "concentrated under reduced pressure" refers to the removal of solvents and volatile materials using a rotary evaporator with the water bath temperature below 30 °C, followed by the removal of residual solvents at high vacuum (< 0.2 mbar).

Proton nuclear magnetic resonance (^{1}H NMR) spectra were acquired on commercial instruments at 400, 500 and 600 MHz. Carbon-13 nuclear magnetic resonance (^{13}C NMR) spectra were acquired at 101, 126, and 151 MHz. The proton signal for the residual non-deuterated solvent (δ 7.26 for $CHCl_3$) was used as an internal reference for ^{1}H NMR spectra. For ^{13}C NMR spectra, chemical shifts are reported relative to the δ 77.36 resonance of $CHCl_3$. Coupling constants are reported in Hz. High-resolution mass spectra were recorded on a commercial high-resolution mass spectrometer (mass analyzer type: QTOF).

Analytical thin layer chromatography (TLC) was performed on Kieselgel 60 F254 glass plates precoated with a 0.25 mm thickness of silica gel. The TLC plates were visualized with UV light and/or by staining with Hanessian solution (ceric sulfate and ammonium molybdate in aqueous sulfuric acid) or $KMnO_4$. Column chromatography was generally performed using Kieselgel 60 (230-400 mesh) silica gel, typically using a 50-100:1 weight ratio of silica gel to crude product.

Chiral phosphoric acid catalysts (*R*)-$\mathbf{A_1}$, (S)-$\mathbf{A_1}$, (*R*)-$\mathbf{A_2}$ and (S)-$\mathbf{A_2}$ were purchased from commercial sources.

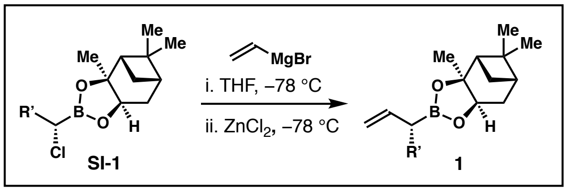

General procedure for syntheses of allylboronates 1: Boronic ester **SI-1**[1] (1 mmol) was dissolved in anhydrous THF (5 mL) in a round bottom flask. The mixture was cooled to −78 °C, and vinyl magnesium bromide (1 mL, 1 mmol, 1 M in THF) was added. After stirring at −78 °C for 30 min, $ZnCl_2$ (1 mL, 1 mmol, 1 M in Et_2O) was added and the mixture was kept stirring at −78 °C for 3 h. Then hexane (15 mL) was added and the resulting mixture was allowed to warm to ambient temperature. The reaction mixture was filtered through a pad of silica gel and the filtrate was concentrated under reduced pressure. Purification of the crude product was performed by column chromatography to provide allylboronates **1**.

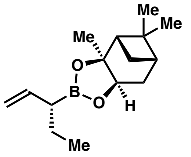

(3a*S*,4*S*,6*S*,7a*R*)-3a,5,5-trimethyl-2-((*R*)-pent-1-en-3-yl)hexahydro-4,6-methanobenz o[*d*][1,3,2]dioxaborole (1b) Prepared according to the general procedure. The crude mixture was purified by flash column chromatography (gradient elution with hexane and Et_2O, 50:1 to 10:1) to give compound **1b** as colorless oil in 95% yield (236 mg). ^1H NMR (400 MHz, $CDCl_3$) δ 5.81 (ddd, *J* = 18.0, 9.4, 9.4 Hz, 1H), 4.94 – 5.02 (m, 2H), 4.27 (d, *J* = 8.6 Hz, 1H), 2.31 – 2.36 (m, 1H), 2.19 – 2.22 (m, 1H), 2.06 (t, *J* = 5.1 Hz, 1H), 1.78 – 1.90 (m, 3H), 1.61 – 1.64 (m, 1H), 1.46 – 1.50 (m, 1H), 1.38 (s, 3H), 1.28 (s, 3H), 1.11 (d, *J* = 10.9 Hz, 1H), 0.93 (t, *J* = 7.3 Hz, 3H), 0.84 (s, 3H). ^{13}C NMR (101 MHz, $CDCl_3$) δ 140.1, 113.9, 85.9, 78.0, 51.6, 39.8, 38.5, 35.9, 29.0, 27.4, 26.8, 24.4, 23.8, 14.0. HRMS (EI^+): *m/z* for $C_{15}H_{25}BO_2$ $[M]^+$ calcd. 248.1948, found: 248.1946.

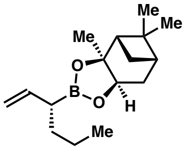

(3a*S*,4*S*,6*S*,7a*R*)-2-((*R*)-hex-1-en-3-yl)-3a,5,5-trimethylhexahydro-4,6-methanobenzo [*d*][1,3,2]dioxaborole (1c) Prepared according to the general procedure. The crude mixture was purified by flash column chromatography (gradient elution with hexane and Et_2O, 50:1 to 10:1) to give compound **1c** as colorless oil in 92% yield (240 mg). ^1H NMR (600 MHz, $CDCl_3$) δ 5.80 (ddd, *J* =17.2, 9.8, 9.8 Hz, 1H), 4.98 (dd, *J* = 17.1, 1.0 Hz, 1H), 4.94 (d, *J* = 10.0, 1H), 4.26 (dd, *J* = 8.7, 1.5 Hz, 1H), 2.31 – 2.35 (m, 1H), 2.19 – 2.21 (m, 1H), 2.05 (t, *J* = 5.6 Hz, 1H), 1.88 – 1.90 (m, 2H), 1.81 – 1.84 (m, 1H), 1.53 – 1.56 (m, 1H), 1.28 – 1.43

(m, 3H), 1.37 (s, 3H), 1.27 (s, 3H), 1.09 (d, *J* = 10.9 Hz, 1H), 0.89 (t, *J* = 7.3 Hz, 3H), 0.83 (s, 3H). ^{13}C NMR (151 MHz, $CDCl_3$) δ 140.2, 113.8, 85.9, 78.0, 51.5, 39.7, 38.5, 35.8, 32.8, 30.1, 28.9, 27.4, 26.7, 24.3, 22.5, 14.5. HRMS (EI^+): *m/z* for $C_{16}H_{27}BO_2$ $[M]^+$ calcd. 262.2104, found: 262.2109.

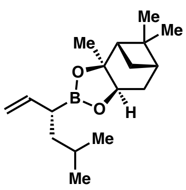

(3a*S*,4*S*,6*S*,7a*R*)-3a,5,5-trimethyl-2-((*R*)-5-methylhex-1-en-3-yl)hexahydro-4,6-methanobenzo[*d*][1,3,2]dioxaborole (1d) Prepared according to the general procedure. The crude mixture was purified by flash column chromatography (gradient elution with hexane and Et_2O, 50:1 to 10:1) to give compound **1d** as colorless oil in 87% yield (241 mg). ^{1}H NMR (600 MHz, $CDCl_3$) δ 5.76 (ddd, *J* = 17.5, 9.6, 9.6 Hz, 1H), 4.98 (d, *J* = 17.1 Hz, 1H), 4.93 (d, *J* = 10.2 Hz, 1H), 4.26 (dd, *J* = 8.8, 1.5 Hz, 1H), 2.30 – 2.34 (m, 1H), 2.19 – 2.21 (m, 1H), 2.05 (t, *J* = 5.5 Hz, 1H), 1.99 (dt, *J* = 8.3, 8.3 Hz, 1H), 1.88 – 1.91 (m, 1H), 1.82 (dt, *J* = 14.6, 2.6 Hz, 1H), 1.56 – 1.62 (m, 1H), 1.37 – 1.39 (m, 2H), 1.36 (s, 3H), 1.27 (s, 3H), 1.08 (d, *J* = 10.9 Hz, 1H), 0.87 (d, *J* = 6.6 Hz, 3H), 0.85 (d, *J* = 6.5 Hz, 3H), 0.83 (s, 3H). ^{13}C NMR (151 MHz, $CDCl_3$) δ 140.2, 113.7, 85.9, 78.0, 51.5, 39.7, 39.5, 38.5, 35.8, 28.9, 28.2, 27.4, 27.0, 26.7, 24.3, 23.4, 22.2. HRMS (EI^+): *m/z* for $C_{17}H_{29}BO_2$ $[M]^+$ calcd. 276.2261, found: 276.2269.

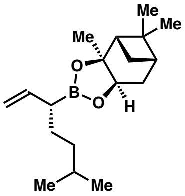

(3a*S*,4*S*,6*S*,7a*R*)-3a,5,5-trimethyl-2-((*R*)-6-methylhept-1-en-3-yl)hexahydro-4,6-methanobenzo[*d*][1,3,2]dioxaborole (1e) Prepared according to the general procedure. The crude mixture was purified by flash column chromatography (gradient elution with hexane and Et_2O, 50:1 to 10:1) to give compound **1e** as colorless oil in 90% yield (262 mg). ^{1}H NMR (600 MHz, $CDCl_3$) δ 5.80 (ddd, *J* = 17.4, 9.6, 9.6 Hz, 1H), 4.99 (d, *J* = 17.1 Hz, 1H), 4.94 (d, *J* = 10.2 Hz, 1H), 4.27 (dd, *J* = 8.8, 1.5 Hz, 1H), 2.31 – 2.35 (m, 1H), 2.19 – 2.21 (m, 1H), 2.05 (t, *J* = 5.5 Hz, 1H), 1.87 – 1.92 (m, 1H), 1.80 – 1.84 (m, 2H), 1.46 – 1.57 (m, 2H), 1.39 – 1.45 (m, 1H), 1.37 (s, 3H), 1.28 (s, 3H), 1.22 – 1.27 (m, 1H), 1.11 – 1.15 (m, 1H), 1.10 (d, *J* = 10.9 Hz, 1H), 0.861 (d, *J* = 6.6, Hz, 3H), 0.858 (d, *J* = 6.6, Hz, 3H), 0.83 (s, 3H). ^{13}C NMR (151 MHz, $CDCl_3$) δ 140.3, 113.7, 85.9, 78.0, 51.5, 39.7, 38.7, 38.5, 35.9, 30.5, 28.9, 28.43, 28.41, 27.4, 26.7, 24.4, 23.03, 22.98. HRMS (EI^+): *m/z* for $C_{18}H_{31}BO_2$ $[M]^+$ calcd. 290.2417, found: 290.2421.

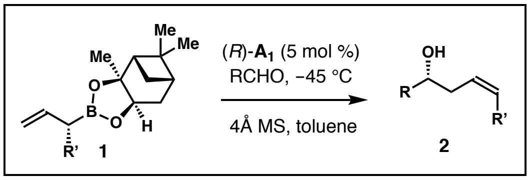

General procedure for the syntheses of (*Z*)-homoallylic alcohols 2: To a reaction flask containing a stirring bar and freshly activated 4 Å MS (50 mg) was added chiral phosphoric acid (*R*)-$\mathbf{A_1}$ (4 mg, 5 mol %). Toluene (0.3 mL) was added to the flask followed by addition of freshly distilled aldehyde (0.1 mmol, 1.0 equiv, if it is a liquid). The mixture was placed in a –45 °C cold bath and stirred for 10 min. Then a solution of allylboronate **1** (0.12 mmol, 1.2 equiv) in toluene (0.2 mL) was added slowly to the reaction mixture *via* a microliter syringe. The mixture was kept stirring at –45 °C for 48 h. After complete consumption of the aldehyde, 3N NaOH (1 mL) was added to the reaction mixture followed by slow addition of 30% H_2O_2 (0.5 mL) at 0 °C. The mixture was stirred vigorously for 2 h. Brine (1 mL) and Et_2O (0.5 mL) were added, the organic layer was separated and the aqueous layer was extracted with Et_2O (3 x 1 mL). The combined organic extracts were dried over anhydrous magnesium sulfate, filtered, and concentrated under reduced pressure. Purification of the crude product was performed by flash column chromatography to provide product **2** or **6**. The enantiopurities of **2** or **6** were determined by Mosher ester analysis.[2]

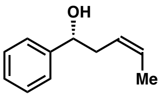

(*R*,*Z*)-1-phenylpent-3-en-1-ol (2a) Prepared according to the general procedure from **1a**.[3] The crude mixture was purified by flash column chromatography (gradient elution with hexane and Et_2O, 20:1 to 4:1) to give compound **2a** as colorless oil in 92% yield (15 mg). The enantiomeric excess was determined to be 98% ee by Mosher ester analysis. $[\alpha]_D^{25}$ = 27.8 (c 0.30, $CHCl_3$). ^1H NMR (600 MHz, $CDCl_3$) δ 7.34 – 7.39 (m, 4H), 7.27 – 7.30 (m, 1H), 5.63 – 5.69 (m, 1H), 5.42 – 5.47 (m, 1H), 4.72 (dd, *J* = 8.0, 5.2 Hz, 1H), 2.57 – 2.59 (m, 1H), 2.46 – 2.48 (m, 1H), 2.01 (brs, 1H), 1.61 (dd, *J* = 6.8, 1.9 Hz, 3H). ^{13}C NMR (151 MHz, $CDCl_3$) δ 144.4, 128.7, 128.2, 127.8, 126.2, 126.0, 74.1, 37.3, 13.4. HRMS (EI^+): *m/z* for $C_{11}H_{12}$ $[M–H_2O]^+$ calcd. 144.0939, found: 144.0938.

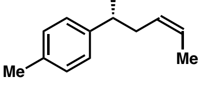

(*R*,*Z*)-1-(*p*-tolyl)pent-3-en-1-ol (2b) Prepared according to the general procedure from **1a**. The crude mixture was purified by flash column chromatography (gradient elution with hexane and Et_2O, 20:1 to 4:1) to give compound **2b** as colorless oil in 91% yield (16 mg). The enantiomeric excess was

determined to be 97% ee by Mosher ester analysis. $[\alpha]_D^{25}$ = 25.5 (c 0.30, $CHCl_3$). ^1H NMR (600 MHz, $CDCl_3$) δ 7.27 (d, J = 8.0 Hz, 2H), 7.17 (d, J = 7.8 Hz, 2H), 5.62 – 5.66 (m, 1H), 5.40 – 5.44 (m, 1H), 4.67 – 4.70 (m, 1H), 2.55 – 2.60 (m, 1H), 2.43 – 2.47 (m, 1H), 2.35 (s, 3H), 1.98 (d, J = 2.8 Hz, 1H), 1.62 (d, J = 5.5 Hz, 3H). ^{13}C NMR (151 MHz, $CDCl_3$) δ 141.4, 137.5, 129.4, 127.9, 126.11, 126.10, 74.0, 37.2, 21.5, 13.4. HRMS (EI^+): *m/z* for $C_{12}H_{14}$ $[M–H_2O]^+$ calcd. 158.1096, found: 158.1091.

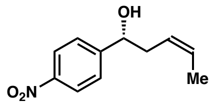

(*R*,*Z*)-1-(4-nitrophenyl)pent-3-en-1-ol (2c) Prepared according to the general procedure from **1a**. The crude mixture was purified by flash column chromatography (gradient elution with hexane and Et_2O, 20:1 to 2:1) to give compound **2c** as colorless oil in 87% yield (18 mg). The enantiomeric excess was determined to be 98% ee by Mosher ester analysis. $[\alpha]_D^{25}$ = 53.0 (c 0.39, $CHCl_3$). ^1H NMR (600 MHz, $CDCl_3$) δ 8.21 (d, J = 8.7 Hz, 2H), 7.55 (d, J = 8.5 Hz, 2H), 5.69 – 5.74 (m, 1H), 5.38 – 5.42 (m, 1H), 4.85 (dd, J = 7.9, 5.2 Hz, 1H), 2.47 – 2.57 (m, 2H), 2.20 (brs, 1H), 1.59 (d, J = 6.8 Hz, 3H). ^{13}C NMR (151 MHz, $CDCl_3$) δ 151.6, 147.5, 129.5, 126.9, 124.7, 124.0, 73.1, 37.4, 13.4. HRMS (EI^+): *m/z* for $C_{11}H_{13}NO_3$ $[M]^+$ calcd. 207.0895, found: 207.0893.

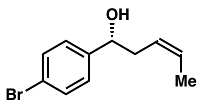

(*R*,*Z*)-1-(4-bromophenyl)pent-3-en-1-ol (2d) Prepared according to the general procedure from **1a**. The crude mixture was purified by flash column chromatography (gradient elution with hexane and Et_2O, 20:1 to 4:1) to give compound **2d** as colorless oil in 79% yield (19 mg). The enantiomeric excess was determined to be 98% ee by Mosher ester analysis. $[\alpha]_D^{25}$ = 60.6 (c 0.60, $CHCl_3$). ^1H NMR (600 MHz, $CDCl_3$) δ 7.47 (d, J = 8.4 Hz, 2H), 7.25 (d, J = 8.9 Hz, 2H), 5.64 – 5.69 (m, 1H), 5.37 – 5.41 (m, 1H), 4.69 (dd, J = 7.7, 5.4 Hz, 1H), 2.51 – 2.56 (m, 1H), 2.41 – 2.45 (m, 1H), 2.03 (brs, 1H), 1.60 (d, J = 6.7 Hz, 3H). ^{13}C NMR (151 MHz, $CDCl_3$) δ 143.3, 131.8, 128.6, 127.9, 125.4, 121.5, 73.4, 37.3, 13.4. HRMS (EI^+): *m/z* for $C_{11}H_{11}Br$ $[M–H_2O]^+$ calcd. 222.0044, found: 222.0036.

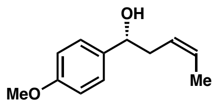

(*R*,*Z*)-1-(4-methoxyphenyl)pent-3-en-1-ol (2e) Prepared according to the general procedure from **1a**. The crude mixture was purified by flash column chromatography (gradient elution with hexane and Et_2O, 20:1 to 2:1) to give compound **2e** as colorless oil in 83% yield (16 mg). The enantiomeric excess was determined to be 98% ee by Mosher ester analysis. $[\alpha]_D^{25}$ = 66.3 (c 0.35, $CHCl_3$). ^1H NMR (600 MHz, $CDCl_3$) δ 7.29 – 7.31 (m, 2H), 6.88 – 6.90 (m, 2H), 5.61 – 5.66 (m, 1H), 5.38 – 5.43 (m, 1H), 4.67 (dd, J = 7.9, 5.4 Hz, 1H), 3.81 (s, 3H), 2.56 –

2.60 (m, 1H), 2.41 – 2.46 (m, 1H), 1.93 (brs, 1H), 1.61 (dd, J = 6.8, 1.7, 3H). ^{13}C NMR (151 MHz, $CDCl_3$) δ 159.3, 136.5, 127.9, 127.4, 126.1, 114.0, 73.8, 55.6, 37.2, 13.4. HRMS (EI^+): m/z for $C_{12}H_{14}O$ $[M–H_2O]^+$ calcd. 174.1045, found: 174.1038.

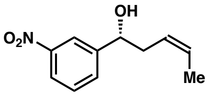

(*R*,*Z*)-1-(3-nitrophenyl)pent-3-en-1-ol (2f) Prepared according to the general procedure from **1a**. The crude mixture was purified by flash column chromatography (gradient elution with hexane and Et_2O, 20:1 to 2:1) to give compound **2f** as colorless oil in 92% yield (19 mg). The enantiomeric excess was determined to be 99% ee by Mosher ester analysis. $[\alpha]_D^{25}$ = 11.2 (c 0.70, $CHCl_3$). ^{1}H NMR (600 MHz, $CDCl_3$) δ 8.26 (s, 1H), 8.12 – 8.14 (m, 1H), 7.71 (d, J = 7.7 Hz, 1H), 7.52 (dd, J = 7.9, 7.9 Hz, 1H), 5.68 – 5.74 (m, 1H), 5.38 – 5.43 (m, 1H), 4.84 (dd, J = 7.7, 5.2 Hz, 1H), 2.54 – 2.59 (m, 1H), 2.48 – 2.52 (m, 1H), 2.23 (brs, 1H), 1.60 (d, J = 6.9 Hz, 3H). ^{13}C NMR (151 MHz, $CDCl_3$) δ 148.5, 146.4, 132.3, 129.6, 129.4, 124.7, 122.8, 121.2, 73.0, 37.4, 13.4. HRMS (EI^+): m/z for $C_{11}H_{11}NO_2$ $[M–H_2O]^+$ calcd. 189.0790, found: 189.0800.

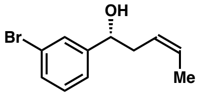

(*R*,*Z*)-1-(3-bromophenyl)pent-3-en-1-ol (2g) Prepared according to the general procedure from **1a**. The crude mixture was purified by flash column chromatography (gradient elution with hexane and Et_2O, 20:1 to 5:1) to give compound **2g** as colorless oil in 83% yield (20 mg). The enantiomeric excess was determined to be 98% ee by Mosher ester analysis. $[\alpha]_D^{25}$ = 45.3 (c 0.35, $CHCl_3$). ^{1}H NMR (600 MHz, $CDCl_3$) δ 7.54 (s, 1H), 7.40 (d, J = 7.8 Hz, 1H), 7.29 (d, J = 7.6 Hz, 1H), 7.22 (dd, J = 7.8, 7.8 Hz, 1H), 5.66 – 5.71 (m, 1H), 5.38 – 5.42 (m, 1H), 4.69 (dd, J = 7.5, 5.3 Hz, 1H), 2.51 – 2.56 (m, 1H), 2.43 – 2.47 (m, 1H), 2.05 (brs, 1H), 1.61 (d, J = 6.8 Hz, 3H). ^{13}C NMR (151 MHz, $CDCl_3$) δ 146.7, 130.8, 130.3, 129.2, 128.8, 125.4, 124.8, 122.8, 73.4, 37.3, 13.4. HRMS (EI^+): m/z for $C_{11}H_{13}BrO$ $[M]^+$ calcd. 240.0150, found: 240.0146.

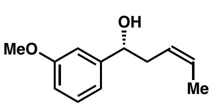

(*R*,*Z*)-1-(3-methoxyphenyl)pent-3-en-1-ol (2h) Prepared according to the general procedure from **1a**. The crude mixture was purified by flash column chromatography (gradient elution with hexane and Et_2O, 20:1 to 2:1) to give **2h** as colorless oil in 94% yield (18 mg). The enantiomeric excess was determined to be 98% ee by Mosher ester analysis. $[\alpha]_D^{25}$ = 44.8 (c 0.46, $CHCl_3$). ^{1}H NMR (600 MHz, $CDCl_3$) δ 7.27 – 7.28 (m, 1H), 6.94 – 6.95 (m, 2H), 6.81 – 6.83 (m, 1H), 5.63 – 5.68 (m, 1H), 5.40 – 5.45 (m, 1H), 4.69 – 4.71 (m, 1H), 3.82 (s, 3H), 2.54 – 2.59 (m, 1H), 2.44 – 2.49 (m, 1H), 2.02 (d, J = 3.0 Hz, 1H), 1.62 (d, J = 6.8 Hz, 3H). ^{13}C

NMR (151 MHz, $CDCl_3$) δ 159.9, 146.1, 129.7, 128.2, 125.9, 118.5, 113.2, 111.6, 74.1, 55.6, 37.2, 13.4. HRMS (EI^+): *m/z* for $C_{12}H_{16}O_2$ $[M]^+$ calcd. 192.1150, found: 192.1151.

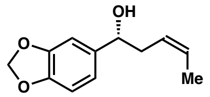

(*R*,*Z*)-1-(benzo[*d*][1,3]dioxol-5-yl)pent-3-en-1-ol (2i) Prepared according to the general procedure from **1a**. The crude mixture was purified by flash column chromatography (gradient elution with hexane and Et_2O, 20:1 to 1:1) to give compound **2i** as colorless oil in 87% yield (18 mg). The enantiomeric excess was determined to be 99% ee by Mosher ester analysis. $[\alpha]_D^{25}$ = 33.9 (c 0.45, $CHCl_3$). ^1H NMR (600 MHz, $CDCl_3$) δ 6.90 (s, 1H), 6.81 – 6.82 (m, 1H), 6.77 (d, *J* = 7.9 Hz, 1H), 5.95 (s, 2H), 5.62 – 5.67 (m, 1H), 5.40 – 5.42 (m, 1H), 4.62 – 4.64 (m, 1H), 2.52 – 2.57 (m, 1H), 2.39 – 2.43 (m, 1H), 1.97 (d, *J* = 2.8 Hz, 1H), 1.62 (d, *J* = 6.9 Hz, 3H). ^{13}C NMR (151 MHz, $CDCl_3$) δ 148.0, 147.2, 138.5, 128.1, 125.9, 119.6, 108.4, 106.7, 101.3, 74.0, 37.2, 13.4. HRMS (EI^+): *m/z* for $C_{12}H_{12}O_2$ $[M–H_2O]^+$ calcd. 188.0837, found: 188.0845.

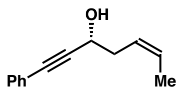

(*R*,*Z*)-1-phenylhept-5-en-1-yn-3-ol (2j) Prepared according to the general procedure from **1a**. The crude mixture was purified by flash column chromatography (gradient elution with hexane and Et_2O, 20:1 to 5:1) to give compound **2j** as colorless oil in 70% yield (13 mg). The enantiomeric excess was determined to be 97% ee by Mosher ester analysis. $[\alpha]_D^{25}$ = 12.6 (c 0.35, $CHCl_3$). ^1H NMR (600 MHz, $CDCl_3$) δ 7.42 – 7.44 (m, 2H), 7.31 – 7.33 (m, 3H), 5.72 – 5.77 (m, 1H), 5.55 – 5.60 (m, 1H), 4.64 (dd, *J* = 6.1, 6.1 Hz, 1H), 2.56 – 2.64 (m, 2H), 2.01 (brs, 1H), 1.70 (d, *J* = 6.8 Hz, 3H). ^{13}C NMR (151 MHz, $CDCl_3$) δ 132.0, 128.83, 128.76, 128.6, 124.6, 122.8, 89.9, 85.2, 62.8, 35.7, 13.6. HRMS (EI^+): *m/z* for $C_{13}H_{14}O$ $[M]^+$ calcd. 186.1045, found: 186.1037.

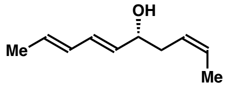

(*R*,2*Z*,6*E*,8*E*)-deca-2,6,8-trien-5-ol (2k) Prepared according to the general procedure from **1a**. The crude mixture was purified by flash column chromatography (gradient elution with hexane and Et_2O, 20:1 to 5:1) to give compound **2k** as colorless oil in 72% yield (11 mg). The enantiomeric excess was determined to be 99% ee by Mosher ester analysis. $[\alpha]_D^{25}$ = 10.0 (c 0.43, $CHCl_3$). ^1H NMR (400 MHz, $CDCl_3$) δ 6.20 (dd, *J* = 15.2, 10.4 Hz, 1H), 6.01 – 6.08 (m, 1H), 5.57 – 5.76 (m, 3H), 5.38 – 5.45 (m, 1H), 4.15 – 4.21 (m, 1H), 2.26 – 2.39 (m, 2H), 1.76 (d, *J* = 6.7 Hz, 3H), 1.64 (d, *J* = 6.7, 3H), 1.59 (d, *J* = 3.9 Hz, 1H). ^{13}C NMR (101 MHz, $CDCl_3$) δ 132.9, 131.2, 131.1, 130.3, 127.7, 125.8, 72.4, 35.3, 18.5, 13.4. HRMS (EI^+): *m/z* for $C_{10}H_{14}$ $[M–H_2O]^+$ calcd. 134.1096, found: 134.1101.

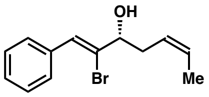

(*R*,1*Z*,5*Z*)-2-bromo-1-phenylhepta-1,5-dien-3-ol (2l) Prepared according to the general procedure from **1a**. The crude mixture was purified by flash column chromatography (gradient elution with hexane and Et_2O, 20:1 to 4:1) to give compound **2l** as colorless oil in 90% yield (24 mg). The enantiomeric excess was determined to be 98% ee by Mosher ester analysis. $[\alpha]_D^{25}$ = 2.5 (c 0.90, $CHCl_3$). ^{1}H NMR (600 MHz, $CDCl_3$) δ 7.60 – 7.62 (m, 2H), 7.34 – 7.38 (m, 2H), 7.31 – 7.33 (m, 1H), 7.06 (s, 1H), 5.67 – 5.70 (m, 1H), 5.41 – 5.43 (m, 1H), 4.30 – 4.32 (m, 1H), 2.56 – 2.60 (m, 2H), 2.16 (d, *J* = 5.4 Hz, 1H), 1.68 (dd, *J* = 6.8, 1.9 Hz, 3H). ^{13}C NMR (151 MHz, $CDCl_3$) δ 135.4, 129.6, 129.5, 128.55, 128.49, 128.47, 128.41, 124.8, 77.3, 33.8, 13.5. HRMS (EI^+): *m/z* for $C_{13}H_{13}Br$ $[M–H_2O]^+$ calcd. 248.0201, found: 248.0208.

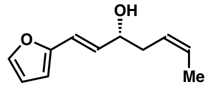

(*R*,1*E*,5*Z*)-1-(furan-2-yl)hepta-1,5-dien-3-ol (2m) Prepared according to the general procedure from **1a**. The crude mixture was purified by flash column chromatography (gradient elution with hexane and Et_2O, 20:1 to 3:1) to give compound **2m** as colorless oil in 84% yield (15 mg). The enantiomeric excess was determined to be 99% ee by Mosher ester analysis. $[\alpha]_D^{25}$ = 39.2 (c 0.25, $CHCl_3$). ^{1}H NMR (600 MHz, $CDCl_3$) δ 7.35 (d, *J* = 1.6 Hz, 1H), 6.44 (dd, *J* = 15.8, 1.3 Hz, 1H), 6.37 (dd, *J* = 3.3, 1.8 Hz, 1H), 6.23 (d, *J* = 3.2 Hz, 1H), 6.20 (dd, *J* = 15.8, 6.0 Hz, 1H), 5.72 – 5.65 (m, 1H), 5.43 – 5.48 (m, 1H), 4.30 – 4.33 (m, 1H), 2.38 – 2.42 (m, 2H), 1.75 (brs, 1H), 1.66 (dd, *J* = 6.8, 1.8 Hz, 3H). ^{13}C NMR (151 MHz, $CDCl_3$) δ 152.7, 142.3, 130.6, 128.2, 125.4, 118.8, 111.6, 108.4, 72.1, 35.3, 13.5. HRMS (EI^+): *m/z* for $C_{11}H_{12}O$ $[M–H_2O]^+$ calcd. 160.0888, found: 160.0892.

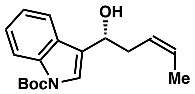

***tert*-butyl (*R*,*Z*)-3-(1-hydroxypent-3-en-1-yl)-1H-indole-1-carboxylate (2n)** Prepared according to the general procedure from **1a**. The crude mixture was purified by flash column chromatography (gradient elution with hexane and Et_2O, 20:1 to 2:1) to give compound **2n** as colorless oil in 93% yield (28 mg). The enantiomeric excess was determined to be 98% ee by Mosher ester analysis. $[\alpha]_D^{25}$ = 29.8 (c 1.20, $CHCl_3$). ^{1}H NMR (600 MHz, $CDCl_3$) δ 8.15 (brs, 1H), 7.69 (d, *J* = 7.8 Hz, 1H), 7.56 (brs, 1H), 7.33 – 7.34 (m, 1H), 7.23 – 7.25 (m, 1H), 5.68 – 5.71 (m, 1H), 5.49 – 5.51 (m, 1H), 5.00 – 5.02 (m, 1H), 2.69 – 2.74 (m, 2H), 2.01 (brs, 1H), 1.66 – 1.68 (m, 12H). ^{13}C NMR (151 MHz, $CDCl_3$) δ 150.0, 136.1, 128.9, 128.1, 125.9, 124.8, 123.7, 122.9, 122.7, 120.1, 115.6, 84.0, 67.9, 35.2, 28.5, 13.5. HRMS (ESI^+): *m/z* for $C_{18}H_{23}NO_3Na$ $[M+Na]^+$ calcd. 324.1576, found: 324.1583.

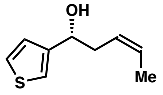

(*R*,*Z*)-1-(thiophen-3-yl)pent-3-en-1-ol (2o) Prepared according to the general procedure from **1a**. The crude mixture was purified by flash column chromatography (gradient elution with hexane and Et_2O, 20:1 to 4:1) to give compound **2o** as colorless oil in 89% yield (15 mg). The enantiomeric excess was determined to be 98% ee by Mosher ester analysis. $[\alpha]_D^{25}$ = 61.3 (c 0.50, $CHCl_3$). ^1H NMR (600 MHz, $CDCl_3$) δ 7.31 (d, *J* = 4.7 Hz, 1H), 7.22 (s, 1H), 7.11 (d, *J* = 4.9 Hz, 1H), 5.64 – 5.69 (m, 1H), 5.40 – 5.45 (m, 1H), 4.81 – 4.83 (m, 1H), 2.57 – 2.62 (m, 1H), 2.51 – 2.55 (m, 1H), 2.00 (brs, 1H), 1.63 (d, *J* = 6.8 Hz, 3H). ^{13}C NMR (151 MHz, $CDCl_3$) δ 145.8, 128.2, 126.4, 126.0, 125.7, 121.0, 70.4, 36.4, 13.4. HRMS (EI^+): *m/z* for $C_9H_{10}S$ $[M–H_2O]^+$ calcd. 150.0503, found: 150.0498.

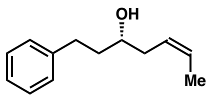

(*S*,*Z*)-1-phenylhept-5-en-3-ol (2p) Prepared according to the general procedure from **1a**. The crude mixture was purified by flash column chromatography (gradient elution with hexane and Et_2O, 20:1 to 5:1) to give compound **2p** as colorless oil in 84% yield (16 mg). The enantiomeric excess was determined to be 99% ee by Mosher ester analysis. $[\alpha]_D^{25}$ = –9.6 (c 0.45, $CHCl_3$). ^1H NMR (600 MHz, $CDCl_3$) δ 7.27 – 7.30 (m, 2H), 7.18 – 7.22 (m, 3H), 5.64 – 5.69 (m, 1H), 5.40 – 5.45 (m, 1H), 3.65 – 3.68 (m, 1H), 2.79 – 2.84 (m, 1H), 2.66 – 2.71 (m, 1H), 2.22 – 2.31 (m, 2H), 1.78 – 1.82 (m, 2H), 1.65 (d, *J* = 6.8 Hz, 3H). ^{13}C NMR (151 MHz, $CDCl_3$) δ 142.4, 128.8, 128.7, 128.0, 126.14, 126.13, 71.0, 38.8, 35.4, 32.5, 13.5. HRMS (EI^+): *m/z* for $C_{13}H_{16}$ $[M–H_2O]^+$ calcd. 172.1252, found: 172.1261.

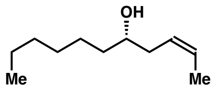

(*S*,*Z*)-undec-2-en-5-ol (2q) Prepared according to the general procedure from **1a**. The crude mixture was purified by flash column chromatography (gradient elution with hexane and Et_2O, 20:1 to 5:1) to give compound **2q** as colorless oil in 82% yield (14 mg). The enantiomeric excess was determined to be 99% ee by Mosher ester analysis. $[\alpha]_D^{25}$ = –3.2 (c 0.37, $CHCl_3$). ^1H NMR (600 MHz, $CDCl_3$) δ 5.63 – 5.68 (m, 1H), 5.41 – 5.46 (m, 1H), 3.60 – 3.65 (m, 1H), 2.21 – 2.23 (m, 2H), 1.64 (d, *J* = 6.8 Hz, 3H), 1.56 (d, *J* = 3.9 Hz, 1H), 1.43 – 1.49 (m, 3H), 1.26 – 1.33 (m, 7H), 0.88 (t, *J* = 6.8 Hz, 3H). ^{13}C NMR (151 MHz, $CDCl_3$) δ 127.8, 126.5, 71.8, 37.2, 35.3, 32.2, 29.7, 26.1, 23.0, 14.5, 13.4. HRMS (EI^+): *m/z* for $C_{11}H_{22}O$ $[M]^+$ calcd. 170.1671, found: 170.1659.

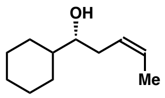

(*R*,*Z*)-1-cyclohexylpent-3-en-1-ol (2r) Prepared according to the general procedure from **1a**. The crude mixture was purified by flash column chromatography (gradient elution with hexane and Et_2O, 20:1 to 5:1) to

give compound **2r** as colorless oil in 77% yield (13 mg). The enantiomeric excess was determined to be 98% ee by Mosher ester analysis. $[\alpha]_D^{25}$ = 5.3 (c 0.65, $CHCl_3$). ^1H NMR (600 MHz, $CDCl_3$) δ 5.64 – 5.67 (m, 1H), 5.43 – 5.45 (m, 1H), 3.36 – 3.38 (m, 1H), 2.22 – 2.24 (m, 2H), 1.86 – 1.88 (m, 1H), 1.74 – 1.77 (m, 2H), 1.65 – 1.69 (m, 2H), 1.64 (d, *J* = 6.8 Hz, 3H), 1.55 (brs, 1H), 1.35 – 1.39 (m, 1H), 1.01 – 1.26 (m, 5H). ^{13}C NMR (151 MHz, $CDCl_3$) δ 127.7, 127.0, 75.9, 43.4, 32.1, 29.5, 28.5, 26.8, 26.6, 26.5, 13.4. HRMS (EI^+): *m/z* for $C_{11}H_{18}$ $[M–H_2O]^+$ calcd. 150.1409, found: 150.1408.

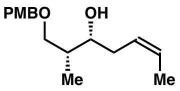

(2*R*,3*R*,*Z*)-1-((4-methoxybenzyl)oxy)-2-methylhept-5-en-3-ol (2s) Prepared according to the general procedure from **1a**. The crude mixture was purified by flash column chromatography (gradient elution with hexane and Et_2O, 20:1 to 2:1) to give compound **2s** as colorless oil in 91% yield (24 mg, dr > 30:1). $[\alpha]_D^{25}$ = 6.0 (c 1.25, $CHCl_3$). ^1H NMR (600 MHz, $CDCl_3$) δ 7.25 (d, *J* = 8.5 Hz, 2H), 6.88 (d, *J* = 8.4 Hz, 2H), 5.56 – 5.61 (m, 1H), 5.41 – 5.45 (m, 1H), 4.42 – 4.46 (m, 2H), 3.80 (s, 3H), 3.78 – 3.80 (m, 1H), 3.50 (d, *J* = 5.2 Hz, 2H), 2.62 (brs, 1H), 2.23 – 2.29 (m, 1H), 2.14 – 2.18 (m, 1H), 1.87 – 1.90 (m, 1H), 1.63 (d, *J* = 6.7 Hz, 3H), 0.96 (d, *J* = 7.1 Hz, 3H). ^{13}C NMR (151 MHz, $CDCl_3$) δ 159.5, 130.4, 129.6, 127.2, 126.7, 114.1, 74.8, 74.1, 73.4, 55.6, 37.6, 32.0, 13.4, 11.1. HRMS (EI^+): *m/z* for $C_{16}H_{24}O_3$ $[M]^+$ calcd. 264.1725, found: 264.1735.

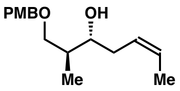

(2*S*,3*R*,*Z*)-1-((4-methoxybenzyl)oxy)-2-methylhept-5-en-3-ol (2t) Prepared according to the general procedure from **1a**. The crude mixture was purified by flash column chromatography (gradient elution with hexane and Et_2O, 20:1 to 2:1) to give compound **2t** as colorless oil in 87% yield (23 mg, dr > 30:1). $[\alpha]_D^{25}$ = 8.2 (c 1.05, $CHCl_3$). ^1H NMR (400 MHz, $CDCl_3$) δ 7.25 (d, *J* = 8.6 Hz, 2H), 6.87 (d, *J* = 8.5 Hz, 2H), 5.56 – 5.64 (m, 1H), 5.46 – 5.53 (m, 1H), 4.45 (s, 2H), 3.80 (s, 3H), 3.55 – 3.60 (m, 2H), 3.46 (dd, *J* = 9.1, 7.2 Hz, 1H), 3.24 (brs, 1H), 2.19 – 2.31 (m, 2H), 1.83 – 1.91 (m, 1H), 1.63 (d, *J* = 6.5 Hz, 3H), 0.92 (d, *J* = 7.0 Hz, 3H). ^{13}C NMR (151 MHz, $CDCl_3$) δ 159.5, 130.1, 129.7, 126.8, 126.5, 114.1, 76.1, 74.9, 73.4, 55.6, 38.1, 32.6, 14.3, 13.4. HRMS (EI^+): *m/z* for $C_{16}H_{24}O_3$ $[M]^+$ calcd. 264.1725, found: 264.1716.

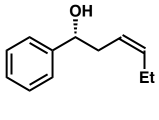

(*R*,*Z*)-1-phenylhex-3-en-1-ol (6a) Prepared according to the general procedure from **1b**. The crude mixture was purified by flash column chromatography (gradient elution with hexane and Et_2O, 20:1 to 5:1) to give compound **6a** as colorless oil in 74% yield (13 mg). The enantiomeric excess was determined to be 98% ee by Mosher ester analysis. $[\alpha]_D^{25}$ = 57.7 (c 0.35, $CHCl_3$). ^1H

NMR (600 MHz, $CDCl_3$) δ 7.34 – 7.38 (m, 4H), 7.26 – 7.29 (m, 1H), 5.55 – 5.59 (m, 1H), 5.35 – 5.38 (m, 1H), 4.69 – 4.72 (m, 1H), 2.54 – 2.57 (m, 1H), 2.45 – 2.48 (m, 1H), 2.02 – 2.06 (m, 3H), 0.92 (t, J = 7.5 Hz, 3H). ^{13}C NMR (151 MHz, $CDCl_3$) δ 144.3, 135.9, 128.7, 127.8, 126.1, 124.3, 74.2, 37.5, 21.0, 14.5. HRMS (EI^+): *m/z* for $C_{12}H_{14}$ $[M–H_2O]^+$ calcd. 158.1096, found: 158.1102.

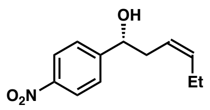

(*R*,*Z*)-1-(4-nitrophenyl)hex-3-en-1-ol (6b) Prepared according to the general procedure from **1b**. The crude mixture was purified by flash column chromatography (gradient elution with hexane and Et_2O, 20:1 to 2:1) to give compound **6b** as colorless oil in 95% yield (21 mg). The enantiomeric excess was determined to be 99% ee by Mosher ester analysis. $[\alpha]_D^{25}$ = 50.2 (c 0.90, $CHCl_3$). ^{1}H NMR (600 MHz, $CDCl_3$) δ 8.21 (d, J = 8.7 Hz, 2H), 7.54 (d, J = 8.5 Hz, 2H), 5.64 – 5.66 (m, 1H), 5.31 – 5.36 (m, 1H), 4.82 – 4.85 (m, 1H), 2.46 – 2.56 (m, 2H), 2.21 (d, J = 2.7 Hz, 1H), 1.97 – 2.03 (m, 2H), 0.91 (t, J = 7.5 Hz, 3H). ^{13}C NMR (151 MHz, $CDCl_3$) δ 151.6, 147.4, 137.2, 126.9, 123.9, 123.0, 73.0, 37.7, 21.0, 14.5. HRMS (ESI^+): *m/z* for $C_{12}H_{16}NO_3$ $[M+H]^+$ calcd. 222.1130, found: 222.1135.

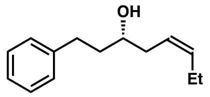

(*S*,*Z*)-1-phenyloct-5-en-3-ol (6c) Prepared according to the general procedure from **1b**. The crude mixture was purified by flash column chromatography (gradient elution with hexane and Et_2O, 20:1 to 5:1) to give compound **6c** as colorless oil in 73% yield (15 mg). The enantiomeric excess was determined to be 94% ee by Mosher ester analysis. $[\alpha]_D^{25}$ = −6.8 (c 0.45, $CHCl_3$). ^{1}H NMR (600 MHz, $CDCl_3$) δ 7.27 – 7.30 (m, 2H), 7.18 – 7.22 (m, 3H), 5.56 – 5.61 (m, 1H), 5.33 – 5.38 (m, 1H), 3.63 – 3.66 (m, 1H), 2.79 – 2.84 (m, 1H), 2.66 – 2.70 (m, 1H), 2.23 – 2.28 (m, 2H), 2.05 – 2.10 (m, 2H), 1.77 – 1.81 (m, 2H), 1.62 (brs, 1H), 0.96 (t, J = 7.5 Hz, 3H). ^{13}C NMR (151 MHz, $CDCl_3$) δ 142.4, 135.9, 128.8, 128.7, 126.1, 124.5, 70.9, 38.7, 35.7, 32.5, 21.1, 14.7. HRMS (EI^+): *m/z* for $C_{14}H_{18}$ $[M–H_2O]^+$ calcd. 186.1409, found: 186.1416.

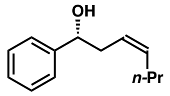

(*R*,*Z*)-1-phenylhept-3-en-1-ol (6d) Prepared according to the general procedure from **1c**. The crude mixture was purified by flash column chromatography (gradient elution with hexane and Et_2O, 20:1 to 5:1) to give compound **6d** as colorless oil in 84% yield (16 mg). The enantiomeric excess was determined to be 97% ee by Mosher ester analysis. $[\alpha]_D^{25}$ = 49.3 (c 0.60, $CHCl_3$). ^{1}H NMR (600 MHz, $CDCl_3$) δ 7.34 – 7.38 (m, 4H), 7.27 – 7.29 (m, 1H), 5.55 – 5.59 (m, 1H), 5.38 – 5.43 (m, 1H), 4.70 – 4.72 (m, 1H), 2.54 – 2.59 (m, 1H), 2.45 – 2.49 (m, 1H), 1.99

– 2.04 (m, 3H), 1.30 – 1.38 (m, 2H), 0.88 (t, J = 7.4 Hz, 3H). ^{13}C NMR (151 MHz, $CDCl_3$) δ 144.3, 134.0, 128.7, 127.8, 126.1, 125.1, 74.2, 37.7, 29.8, 23.0, 14.1. HRMS (EI^+): m/z for $C_{13}H_{16}$ $[M–H_2O]^+$ calcd. 172.1252, found: 172.1260.

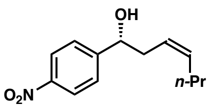

(*R*,*Z*)-1-(4-nitrophenyl)hept-3-en-1-ol (6e) Prepared according to the general procedure from **1c**. The crude mixture was purified by flash column chromatography (gradient elution with hexane and Et_2O, 20:1 to 2:1) to give compound **6e** as colorless oil in 94% yield (22 mg). The enantiomeric excess was determined to be 99% ee by Mosher ester analysis. $[\alpha]_D^{25}$ = 56.9 (c 0.85, $CHCl_3$). ^{1}H NMR (600 MHz, $CDCl_3$) δ 8.21 (d, J = 8.7 Hz, 2H), 7.54 (d, J = 8.7 Hz, 2H), 5.61 – 5.65 (m, 1H), 5.35 – 5.40 (m, 1H), 4.83 (dd, J = 6.3, 6.3 Hz, 1H), 2.46 – 2.56 (m, 2H), 2.20 (s, 1H), 1.93 – 2.02 (m, 2H), 1.29 – 1.36 (m, 2H), 0.87 (t, J = 7.4 Hz, 3H). ^{13}C NMR (151 MHz, $CDCl_3$) δ 151.6, 147.4, 135.3, 126.9, 123.9, 123.8, 73.1, 37.8, 29.7, 23.0 14.1. HRMS (ESI^+): m/z for $C_{13}H_{18}NO_3$ $[M+H]^+$ calcd. 236.1287, found: 236.1295.

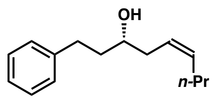

(*S*,*Z*)-1-phenylnon-5-en-3-ol (6f) Prepared according to the general procedure from **1c**. The crude mixture was purified by flash column chromatography (gradient elution with hexane and Et_2O, 20:1 to 5:1) to give compound **6f** as colorless oil in 73% yield (16 mg). The enantiomeric excess was determined to be 95% ee by Mosher ester analysis. $[\alpha]_D^{25}$ = −7.7 (c 0.40, $CHCl_3$). ^{1}H NMR (600 MHz, $CDCl_3$) δ 7.27 – 7.30 (m, 2H), 7.18 – 7.22 (m, 3H), 5.56 – 5.59 (m, 1H), 5.39 – 5.42 (m, 1H), 3.63 – 3.66 (m, 1H), 2.79 – 2.83 (m, 1H), 2.66 – 2.71 (m, 1H), 2.23 – 2.28 (m, 2H), 2.02 – 2.05 (m, 2H), 1.77 – 1.81 (m, 2H), 1.60 (d, J = 4.1 Hz, 1H), 1.36 – 1.39 (m, 2H), 0.90 (t, J = 7.4 Hz, 3H). ^{13}C NMR (151 MHz, $CDCl_3$) δ 142.4, 134.1, 128.8, 128.7, 126.1, 125.3, 71.0, 38.8, 35.8, 32.5, 29.8, 23.2, 14.2. HRMS (EI^+): m/z for $C_{15}H_{20}$ $[M–H_2O]^+$ calcd. 200.1565, found: 200.1576.

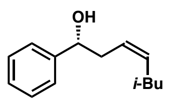

(*R*,*Z*)-6-methyl-1-phenylhept-3-en-1-ol (6g) Prepared according to the general procedure from **1d**. The crude mixture was purified by flash column chromatography (gradient elution with hexane and Et_2O, 20:1 to 5:1) to give compound **6g** as colorless oil in 83% yield (17 mg). The enantiomeric excess was determined to be 98% ee by Mosher ester analysis. $[\alpha]_D^{25}$ = 51.7 (c 0.40, $CHCl_3$). ^{1}H NMR (400 MHz, $CDCl_3$) δ 7.33 – 7.39 (m, 4H), 7.28 – 7.31 (m, 1H), 5.54 – 5.61 (m, 1H), 5.41 – 5.48 (m, 1H), 4.69 – 4.73 (m, 1H), 2.53 – 2.59 (m, 1H), 2.45 – 2.50 (m, 1H), 1.98 (d, J = 3.1 Hz, 1H), 1.93 (dd, J = 7.0, 7.0 Hz, 2H), 1.55 – 1.62 (m, 1H), 0.88 (d, J = 7.6 Hz, 3H), 0.86 (d, J = 6.8 Hz, 3H). ^{13}C NMR (101 MHz, $CDCl_3$) δ 144.4, 132.8, 128.7,

127.8, 126.2, 125.7, 74.3, 37.7, 36.8, 28.9, 22.7, 22.6. HRMS (EI^+): *m/z* for $C_{14}H_{18}$ [M–H_2O]$^+$ calcd. 186.1409, found: 186.1412.

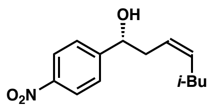

(*R*,*Z*)-6-methyl-1-(4-nitrophenyl)hept-3-en-1-ol (6h) Prepared according to the general procedure from **1d**. The crude mixture was purified by flash column chromatography (gradient elution with hexane and Et_2O, 20:1 to 2:1) to give compound **6h** as colorless oil in 88% yield (22 mg). The enantiomeric excess was determined to be 99% ee by Mosher ester analysis. $[\alpha]_D^{25}$ = 51.3 (c 0.50, $CHCl_3$). ^{1}H NMR (400 MHz, $CDCl_3$) δ 8.21 (d, *J* = 8.7 Hz, 2H), 7.54 (d, *J* = 8.6 Hz, 2H), 5.61 – 5.68 (m, 1H), 5.38 – 5.45 (m, 1H), 4.82 – 4.86 (m, 1H), 2.47 – 2.56 (m, 2H), 2.14 (d, *J* = 3.2 Hz, 1H), 1.90 (dd, *J* = 6.9, 6.9 Hz, 2H), 1.56 – 1.62 (m, 1H), 0.88 (d, *J* = 6.9 Hz, 3H), 0.86 (d, *J* = 6.8 Hz, 3H). ^{13}C NMR (101 MHz, $CDCl_3$) δ 151.7, 147.5, 134.1, 126.9, 124.4, 123.9, 73.2, 37.8, 36.8, 28.9, 22.7, 22.6. HRMS (EI^+): *m/z* for $C_{14}H_{19}NO_3$ [M]$^+$ calcd. 249.1365, found: 249.1371.

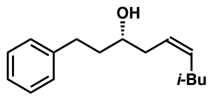

(*S*,*Z*)-8-methyl-1-phenylnon-5-en-3-ol (6i) Prepared according to the general procedure from **1d**. The crude mixture was purified by flash column chromatography (gradient elution with hexane and Et_2O, 20:1 to 5:1) to give compound **6i** as colorless oil in 77% yield (18 mg). The enantiomeric excess was determined to be 94% ee by Mosher ester analysis. $[\alpha]_D^{25}$ = −8.3 (c 0.40, $CHCl_3$). ^{1}H NMR (600 MHz, $CDCl_3$) δ 7.27 – 7.30 (m, 2H), 7.18 – 7.22 (m, 3H), 5.57 – 5.60 (m, 1H), 5.43 – 5.46 (m, 1H), 3.63 – 3.66 (m, 1H), 2.79 – 2.83 (m, 1H), 2.66 – 2.71 (m, 1H), 2.22 – 2.26 (m, 2H), 1.95 (dd, *J* = 7.1, 7.1 Hz, 2H), 1.77 – 1.81 (m, 2H), 1.60 – 1.64 (m, 1H), 0.89 (d, *J* = 6.5 Hz, 3H), 0.88 (d, *J* = 6.5 Hz, 3H). ^{13}C NMR (151 MHz, $CDCl_3$) δ 142.4, 133.0, 128.8, 128.7, 126.1, 125.8, 71.1, 38.8, 36.8, 35.9, 32.5, 29.0, 22.8, 22.7. HRMS (EI^+): *m/z* for $C_{16}H_{22}$ [M–H_2O]$^+$ calcd. 214.1722, found: 214.1728.

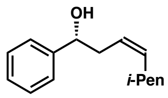

(*R*,*Z*)-7-methyl-1-phenyloct-3-en-1-ol (6j). Prepared according to the general procedure from **1e**. The crude mixture was purified by flash column chromatography (gradient elution with hexane and Et_2O, 20:1 to 5:1) to give compound **6j** as colorless oil in 87% yield (19 mg). The enantiomeric excess was determined to be 98% ee by Mosher ester analysis. $[\alpha]_D^{25}$ = 55.1 (c 0.65, $CHCl_3$). ^{1}H NMR (400 MHz, $CDCl_3$) δ 7.33 – 7.38 (m, 4H), 7.28 – 7.30 (m, 1H), 5.52 – 5.58 (m, 1H), 5.35 – 5.41 (m, 1H), 4.69 – 4.73 (m, 1H), 2.51 – 2.61 (m, 1H), 2.45 – 2.50 (m, 1H), 1.99 – 2.04 (m, 3H), 1.49 – 1.56 (m, 1H), 1.14 – 1.21 (m, 2H), 0.87 (d, *J* = 6.6 Hz, 6H). ^{13}C NMR (101 MHz, $CDCl_3$) δ 144.4, 134.3, 128.7, 127.8, 126.2, 124.7, 74.3, 39.1, 37.6,

28.0, 25.6, 22.84, 22.83. HRMS (EI$^+$): *m/z* for $C_{15}H_{20}$ [M–H_2O]$^+$ calcd. 200.1565, found: 200.1575.

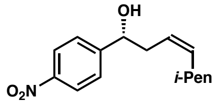

(*R*,*Z*)-7-methyl-1-(4-nitrophenyl)oct-3-en-1-ol (6k) Prepared according to the general procedure from **1e**. The crude mixture was purified by flash column chromatography (gradient elution with hexane and Et_2O, 20:1 to 2:1) to give compound **6k** as colorless oil in 95% yield (25 mg). The enantiomeric excess was determined to be 99% ee by Mosher ester analysis. $[\alpha]_D^{25}$ = 54.2 (c 1.00, $CHCl_3$). ^{1}H NMR (400 MHz, $CDCl_3$) δ 8.21 (d, *J* = 8.6 Hz, 2H), 7.54 (d, *J* = 8.6 Hz, 2H), 5.58 – 5.64 (m, 1H), 5.32 – 5.38 (m, 1H), 4.82 – 4.85 (m, 1H), 2.46 – 2.58 (m, 2H), 2.18 (d, *J* = 3.2 Hz, 1H), 1.95 – 2.01 (m, 2H), 1.45 – 1.55 (m, 1H), 1.11 – 1.21 (m, 2H), 0.85 (d, *J* = 6.6 Hz, 6H). ^{13}C NMR (101 MHz, $CDCl_3$) δ 151.7, 147.5, 135.5, 126.9, 123.9, 123.4, 73.1, 39.0, 37.7, 27.9, 25.6, 22.79, 22.77. HRMS (ESI$^+$): *m/z* for $C_{15}H_{22}NO_3$ [M+H]$^+$ calcd. 264.1600, found: 264.1609.

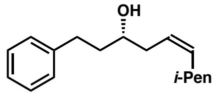

(*S*,*Z*)-9-methyl-1-phenyldec-5-en-3-ol (6l) Prepared according to the general procedure from **1e**. The crude mixture was purified by flash column chromatography (gradient elution with hexane and Et_2O, 20:1 to 5:1) to give compound **6l** as colorless oil in 81% yield (20 mg). The enantiomeric excess was determined to be 95% ee by Mosher ester analysis. $[\alpha]_D^{25}$ = –5.9 (c 0.45, $CHCl_3$). ^{1}H NMR (600 MHz, $CDCl_3$) δ 7.27 – 7.30 (m, 2H), 7.18 – 7.22 (m, 3H), 5.56 – 5.58 (m, 1H), 5.36 – 5.38 (m, 1H), 3.64 – 3.66 (m, 1H), 2.79 – 2.82 (m, 1H), 2.67 – 2.71 (m, 1H), 2.23 – 2.27 (m, 2H), 2.03 – 2.07 (m, 2H), 1.77 – 1.82 (m, 2H), 1.52 – 1.57 (m, 1H), 1.20 – 1.24 (m, 2H), 0.87 (d, *J* = 6.6 Hz, 6H). ^{13}C NMR (151 MHz, $CDCl_3$) δ 142.4, 134.4, 128.8, 128.7, 126.1, 124.9, 71.0, 39.2, 38.7, 35.8, 32.5, 28.0, 25.7, 22.9, 22.8. HRMS (EI$^+$): *m/z* for $C_{17}H_{24}$ [M–H_2O]$^+$ calcd. 228.1878 found: 228.1869.

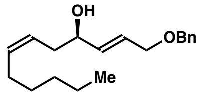

(*R*,2*E*,6*Z*)-1-(benzyloxy)dodeca-2,6-dien-4-ol (8) Prepared according to the general procedure from **1f**. The crude mixture was purified by flash column chromatography (gradient elution with hexane and Et_2O, 20:1 to 2:1) to give compound **8** as colorless oil in 73% yield (21 mg). The enantiomeric excess was determined to be 99% ee by Mosher ester analysis. $[\alpha]_D^{25}$ = 7.4 (c 0.65, $CHCl_3$). ^{1}H NMR (600 MHz, $CDCl_3$) δ 7.34 – 7.37 (m, 4H), 7.28 – 7.30 (m, 1H), 5.78 – 5.86 (m, 2H), 5.55 – 5.60 (m, 1H), 5.36 – 5.40 (m, 1H), 4.52 (s, 2H), 4.16 – 4.20 (m, 1H), 4.03 (d, *J* = 4.3 Hz, 2H), 2.28 – 2.36 (m, 2H), 2.02 – 2.06 (m, 2H), 1.67 (d, *J* = 3.3 Hz, 1H), 1.24 – 1.36 (m, 6H), 0.87 (t, *J* = 6.8 Hz, 3H). ^{13}C NMR (151 MHz, $CDCl_3$) δ 138.4,

135.6, 134.3, 128.7, 128.1, 128.0, 127.5, 124.5, 72.6, 72.0, 70.4, 35.4, 31.8, 29.6, 27.7, 22.9, 14.5. HRMS (EI^+): *m/z* for $C_{19}H_{26}O$ $[M–H_2O]^+$ calcd. 270.1984, found: 270.1991.

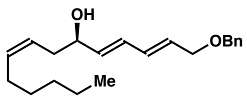

(*R*,2*E*,4*E*,8*Z*)-1-(benzyloxy)tetradeca-2,4,8-trien-6-ol (10) Prepared according to the general procedure from **1f**. The crude mixture was purified by flash column chromatography (gradient elution with hexane and Et_2O, 20:1 to 2:1) to give compound **10** as colorless oil in 70% yield (22 mg). The enantiomeric excess was determined to be 95% ee by Mosher ester analysis. $[\alpha]_D^{25}$ = 2.2 (c 0.30, $CHCl_3$). ^1H NMR (600 MHz, $CDCl_3$) δ 7.34 – 7.35 (m, 4H), 7.29 – 7.30 (s, 1H), 6.24 – 6.29 (m, 2H), 5.80 – 5.83 (m, 1H), 5.72 – 5.75 (m, 1H), 5.55 – 5.58 (m, 1H), 5.36 – 5.39 (m, 1H), 4.52 (s, 2H), 4.19 – 4.20 (m, 1H), 4.06 (d, *J* = 5.8 Hz, 2H), 2.29 – 2.36 (m, 2H), 2.02 – 2.06 (m, 2H), 1.67 (brs, 1H), 1.24 – 1.36 (m, 6H), 0.88 (t, *J* = 6.8 Hz, 3H). ^{13}C NMR (151 MHz, $CDCl_3$) δ 138.4, 136.1, 134.3, 132.4, 130.2, 130.0, 128.7, 128.1, 128.0, 124.4, 72.4, 72.2, 70.6, 35.6, 31.8, 29.6, 27.7, 22.9, 14.5. HRMS (EI^+): *m/z* for $C_{21}H_{30}O_2$ $[M]^+$ calcd. 314.2246, found: 314.2255.

References:

1. (a) Matteson, D. S.; Sadhu, K. M.; Peterson, M. L. *J. Am. Chem. Soc.* **1986**, *108*, 810. (b) Maurer, K. W.; Armstrong, R. W. *J. Org. Chem.* **1996**, *61*, 3106.

2. (a) Dale, J. A.; Mosher, H. S. *J. Am. Chem. Soc.* **1973**, *95*, 512. (b) Ohtani, I.; Kusumi, T.; Kashman, Y.; Kakisawa, H. *J. Am. Chem. Soc.* **1991**, *113*, 4092. (c) Hoye, T. R.; Jeffrey, C. S.; Shao, F. *Nat. Protoc.* **2007**, *2,* 2451.

3. Chen, M.; Roush, W. R. *Org. Lett.* **2010**, *12*, 2706.

Assignment of the absolute configuration of the secondary alcohol group of homoallylic alcohols 2, 3, 6, 8, and 10 using Mosher ester analysis: [2]

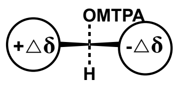

$\triangle\delta = \delta_{S\text{-ester}} - \delta_{R\text{-ester}}$

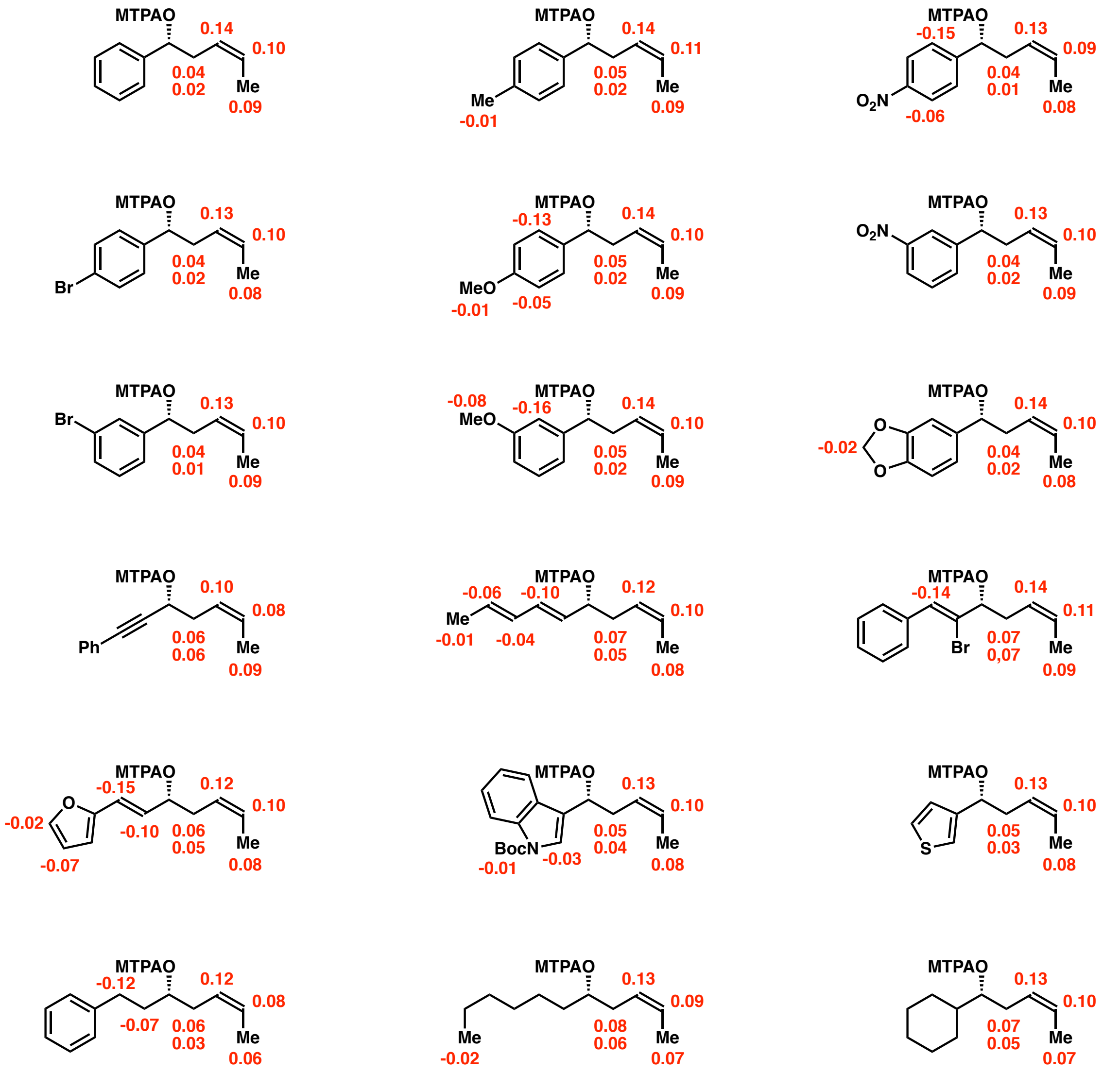

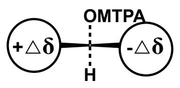

$\Delta\delta = \delta_{S\text{-ester}} - \delta_{R\text{-ester}}$

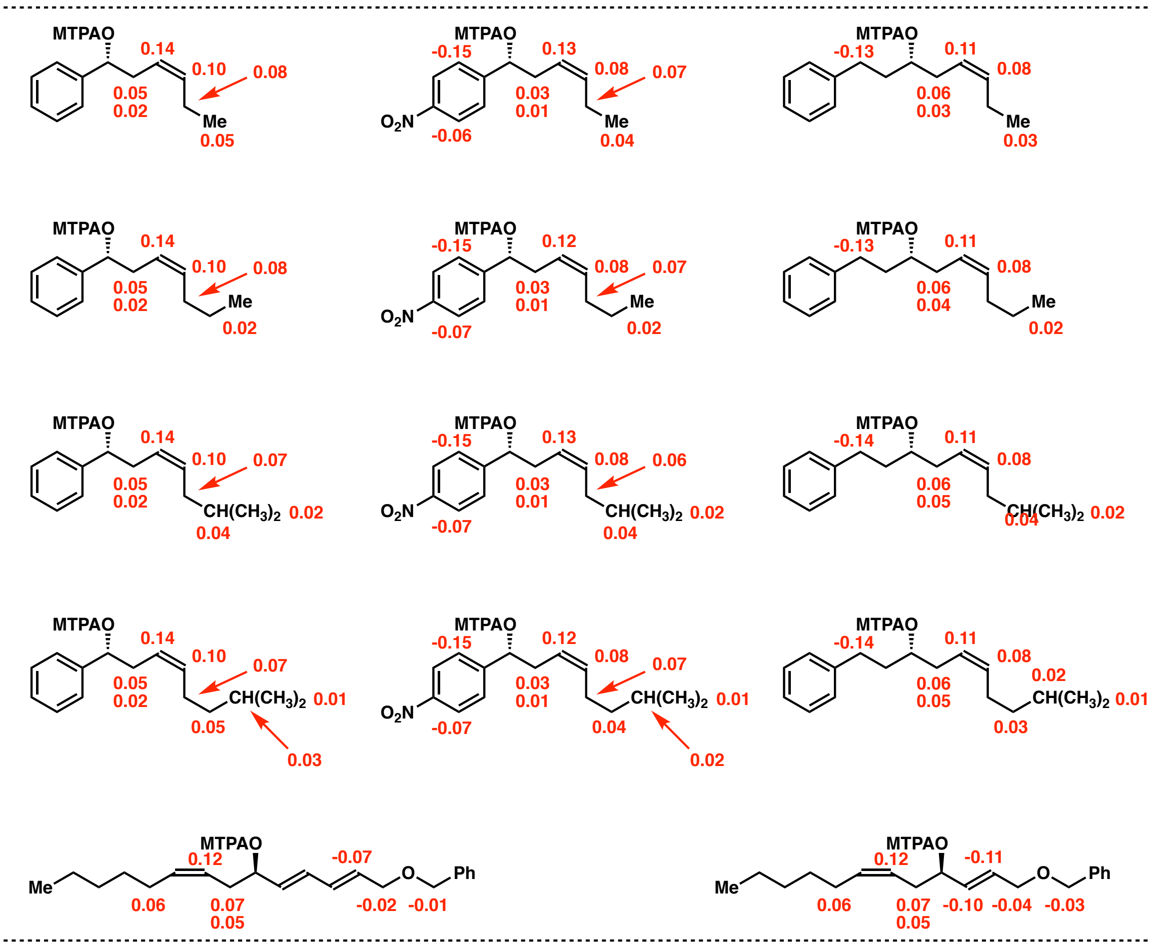

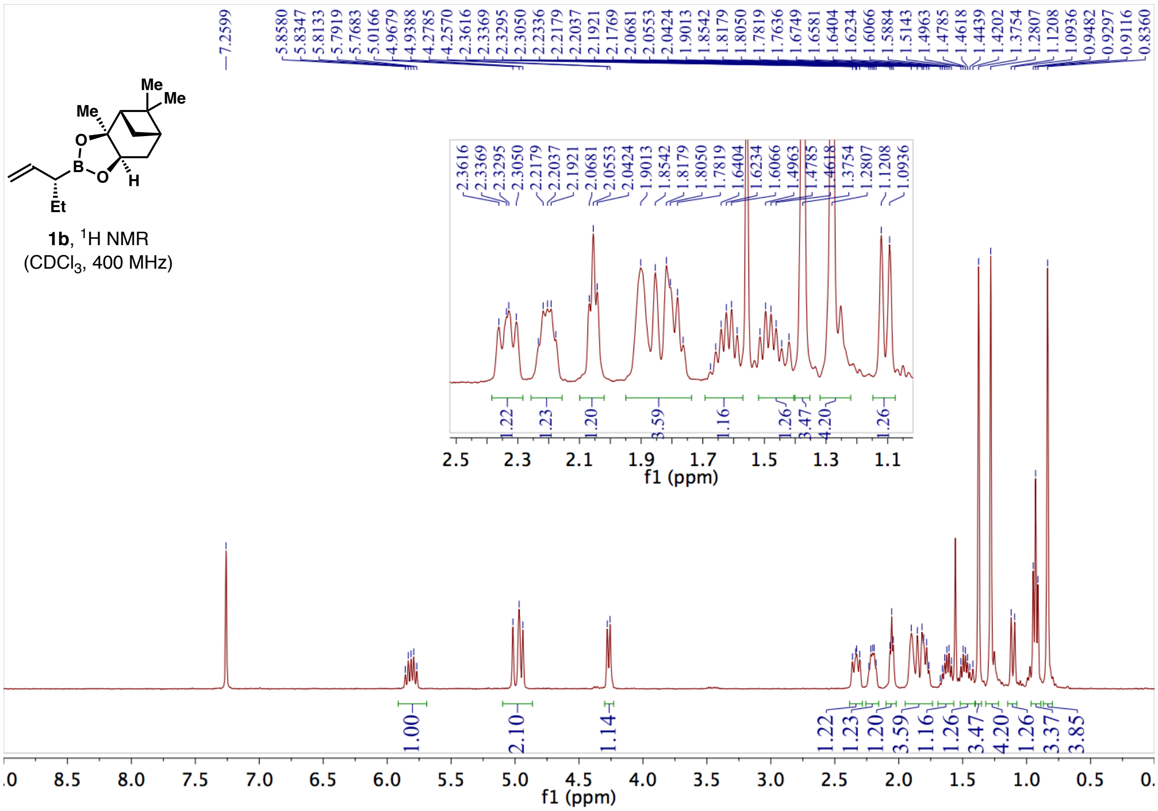
1b, ¹H NMR
(CDCl3, 400 MHz)
f1 (ppm)

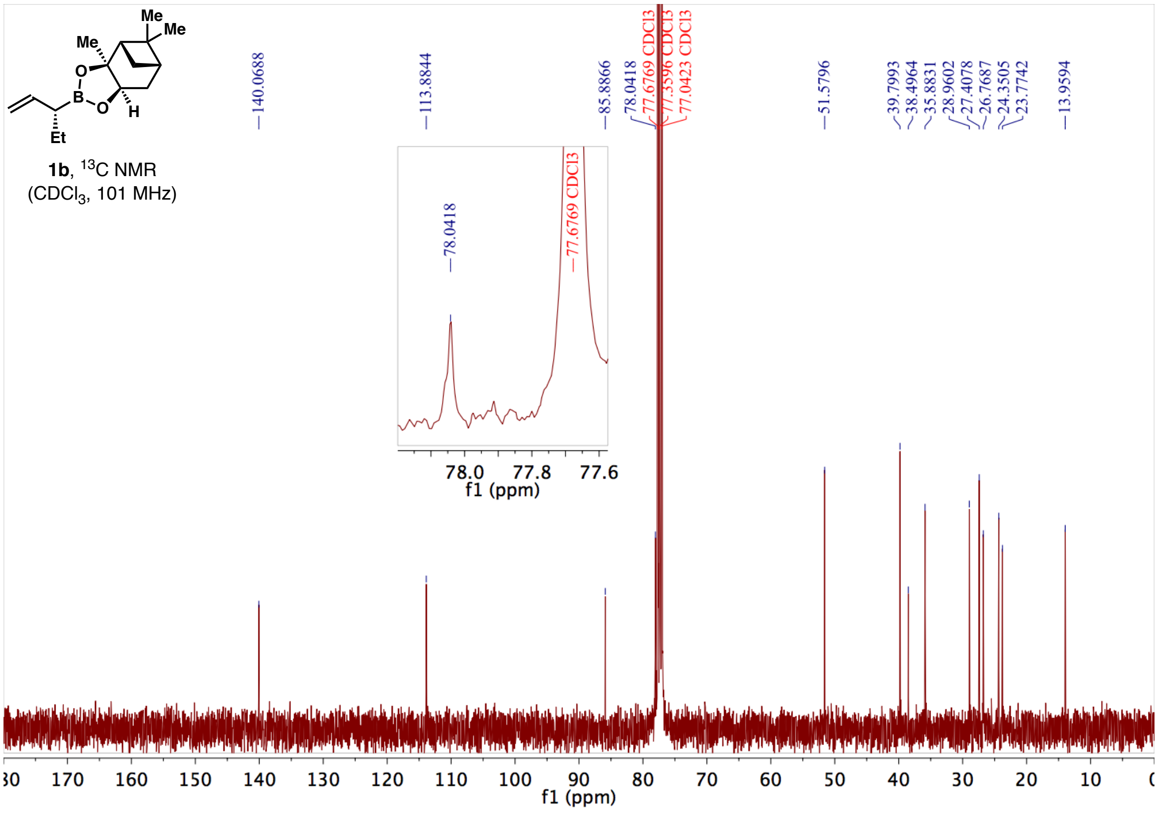
1b, ¹³C NMR
(CDCl3, 101 MHz)
f1 (ppm)

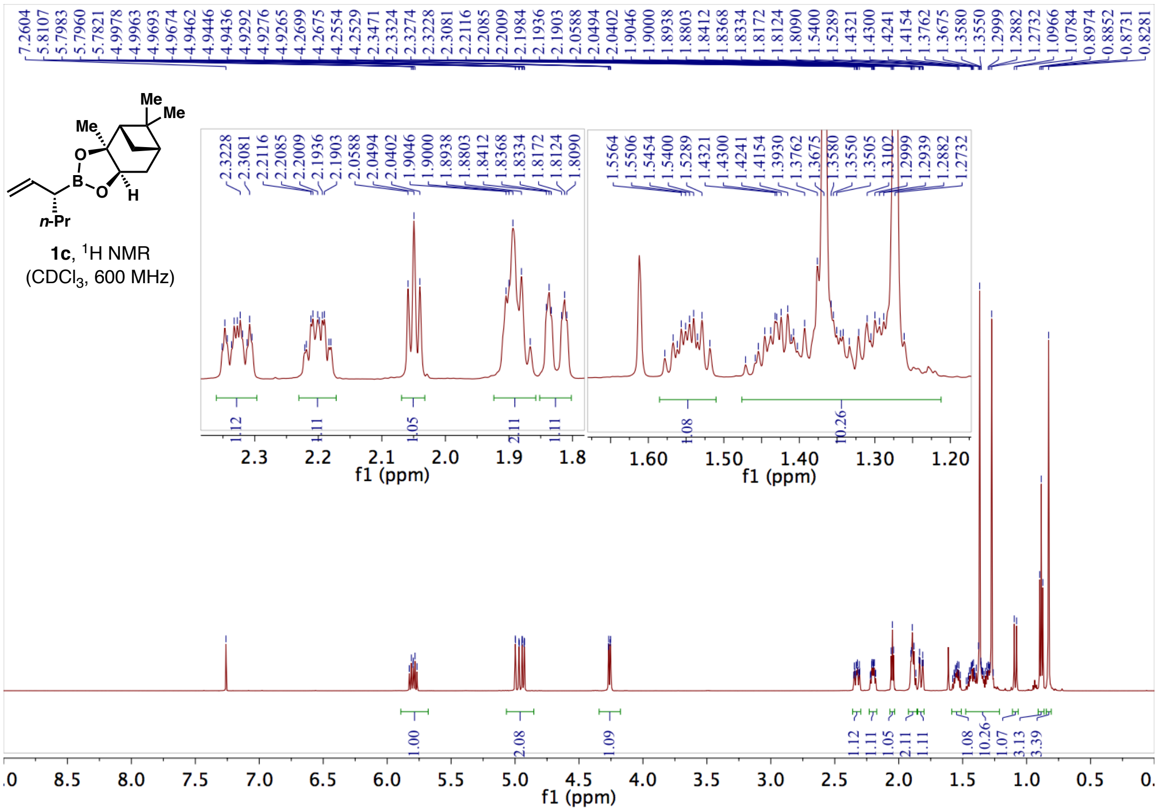

1c, ¹H NMR
(CDCl3, 600 MHz)
f1 (ppm)

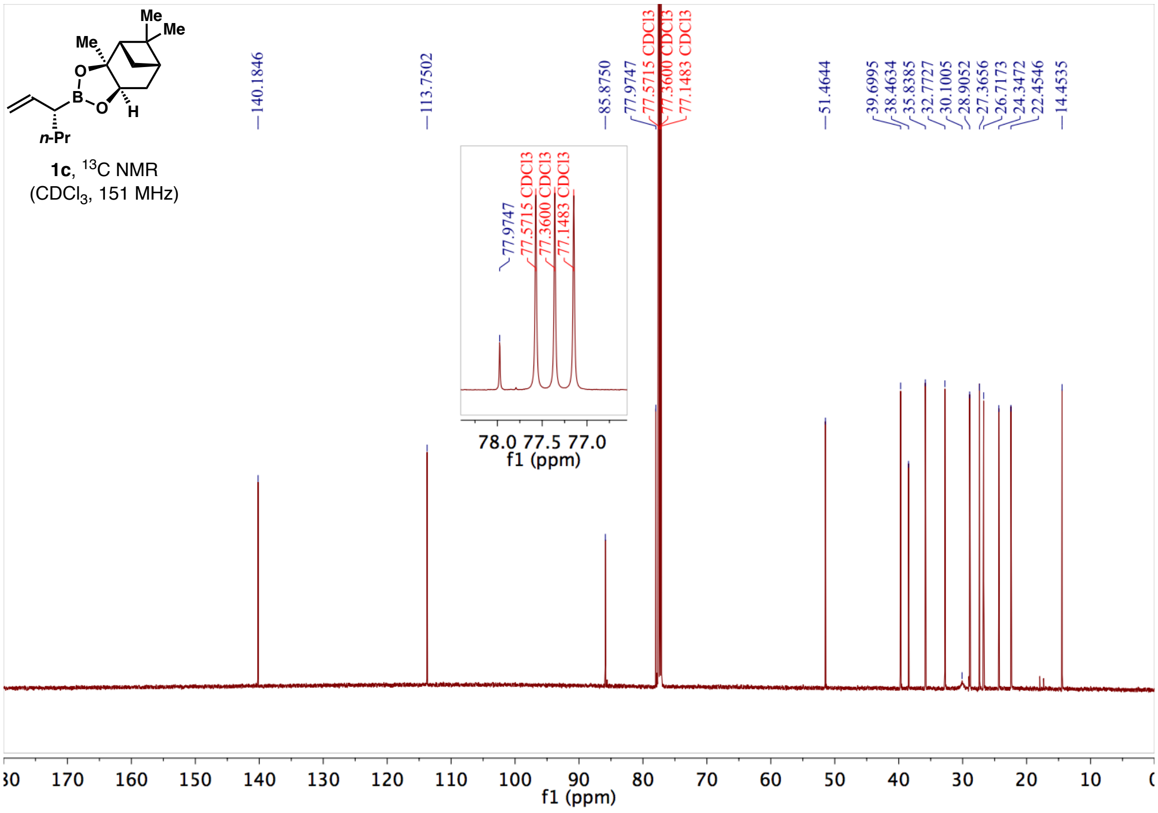

1c, ¹³C NMR
(CDCl3, 151 MHz)
140.1846
113.7502
85.8750
77.9747
77.5715 CDCl3
77.3600 CDCl3
77.1483 CDCl3
51.4644
39.6995
38.4634
35.8385
32.7727
30.1005
28.9052
27.3656
26.7173
24.3472
22.4546
14.4535
f1 (ppm)

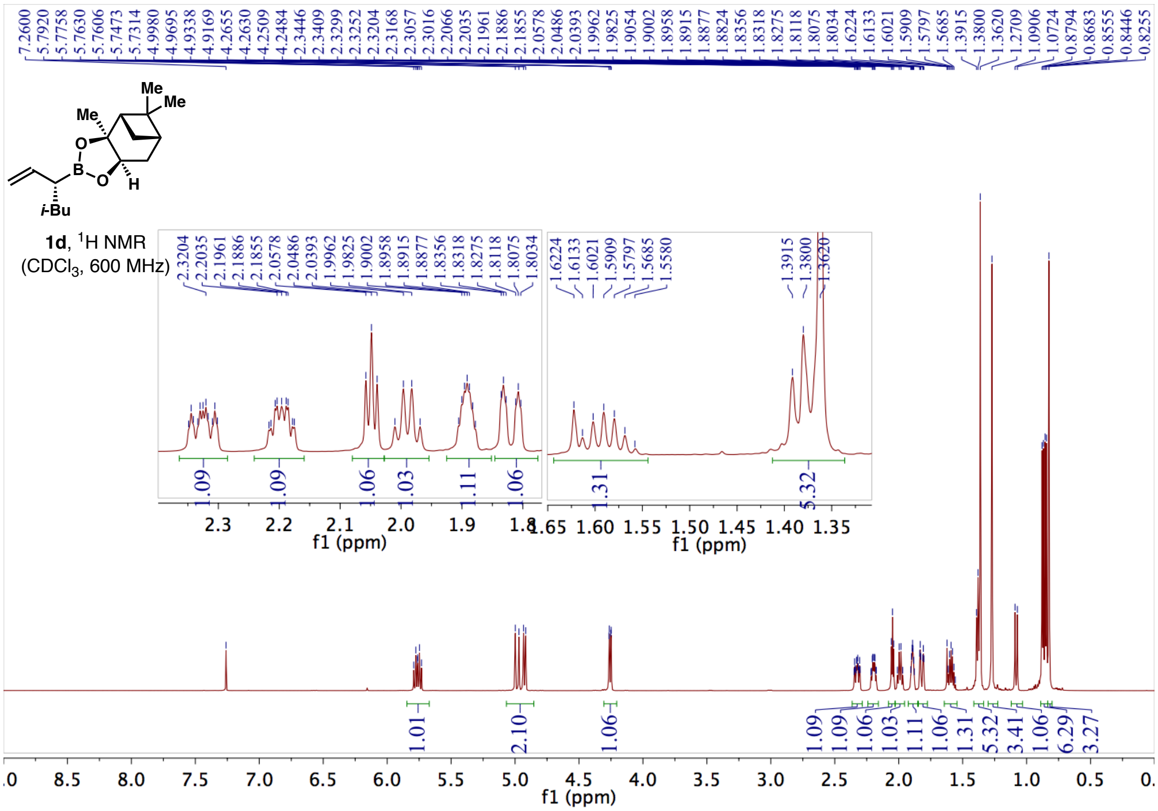
Me
Me
Me
O
B
O
H
i-Bu
1d, ^{1}H NMR
(CDCl$_3$, 600 MHz)
f1 (ppm)

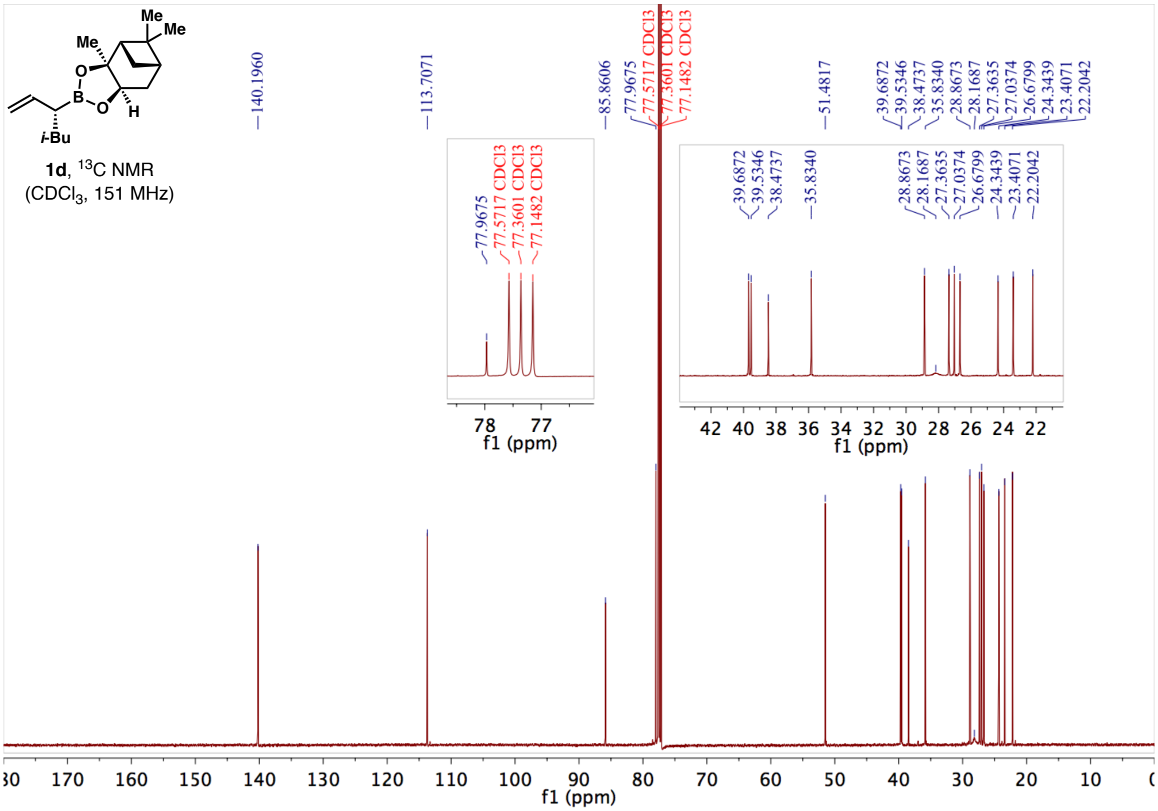
Me
Me
Me
O
B
O
H
i-Bu
1d, ^{13}C NMR
(CDCl$_3$, 151 MHz)
140.1960
113.7071
85.8606
77.9675
77.5717 CDCl3
77.3601 CDCl3
77.1482 CDCl3
51.4817
39.6872
39.5346
38.4737
35.8340
28.8673
28.1687
27.3635
27.0374
26.6799
24.3439
23.4071
22.2042
f1 (ppm)

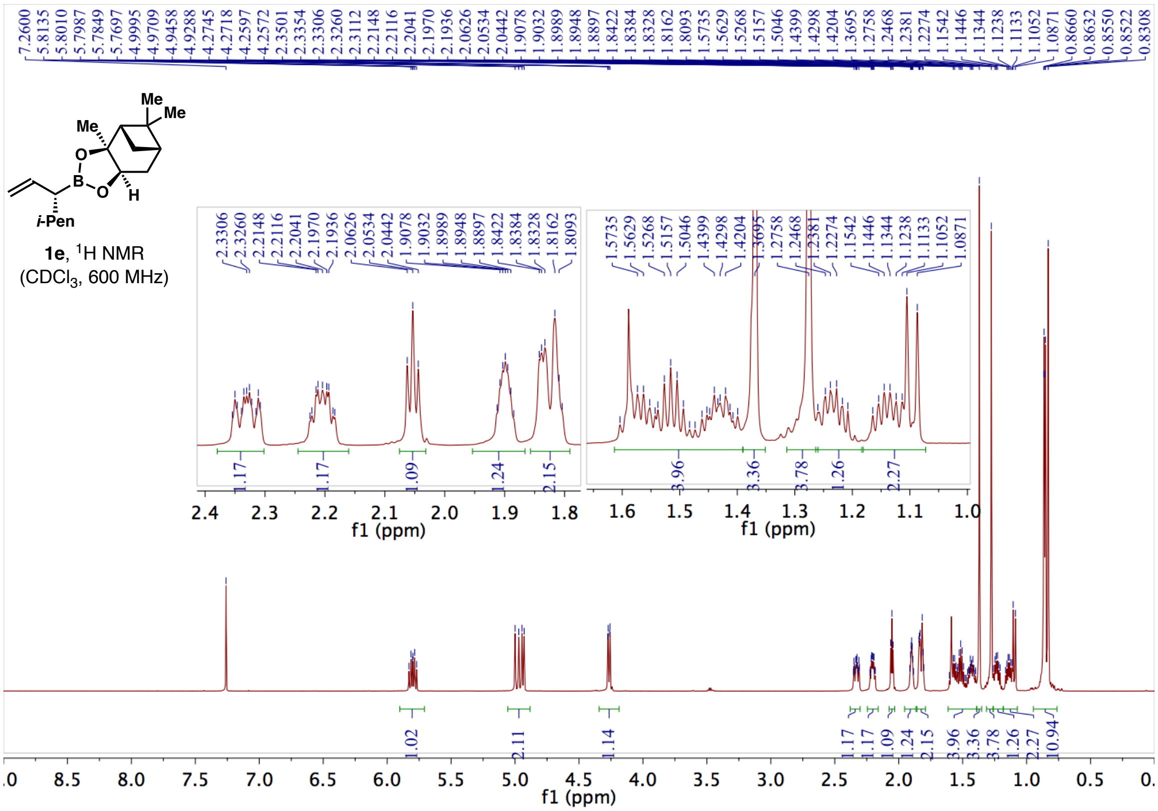
Me
Me
Me
O
B
O
H
i-Pen
1e, ¹H NMR
(CDCl3, 600 MHz)
f1 (ppm)

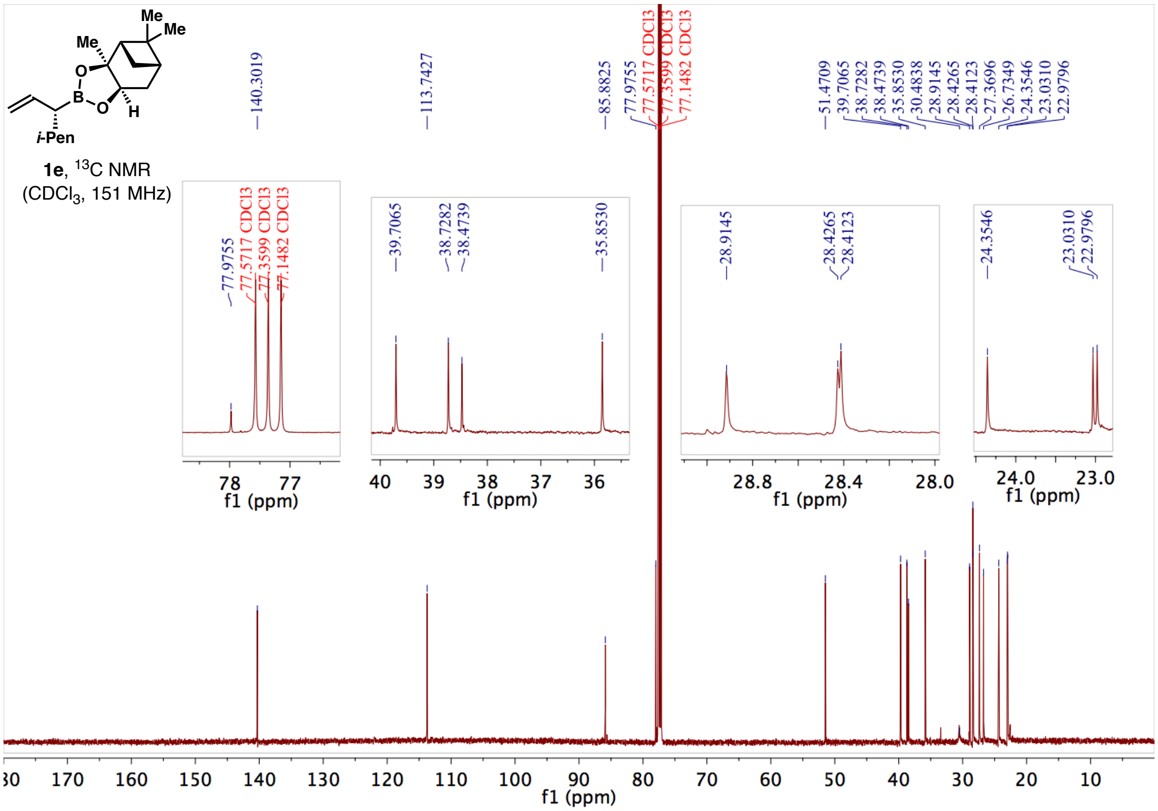
Me
Me
Me
O
B
O
H
i-Pen
1e, ¹³C NMR
(CDCl3, 151 MHz)
f1 (ppm)

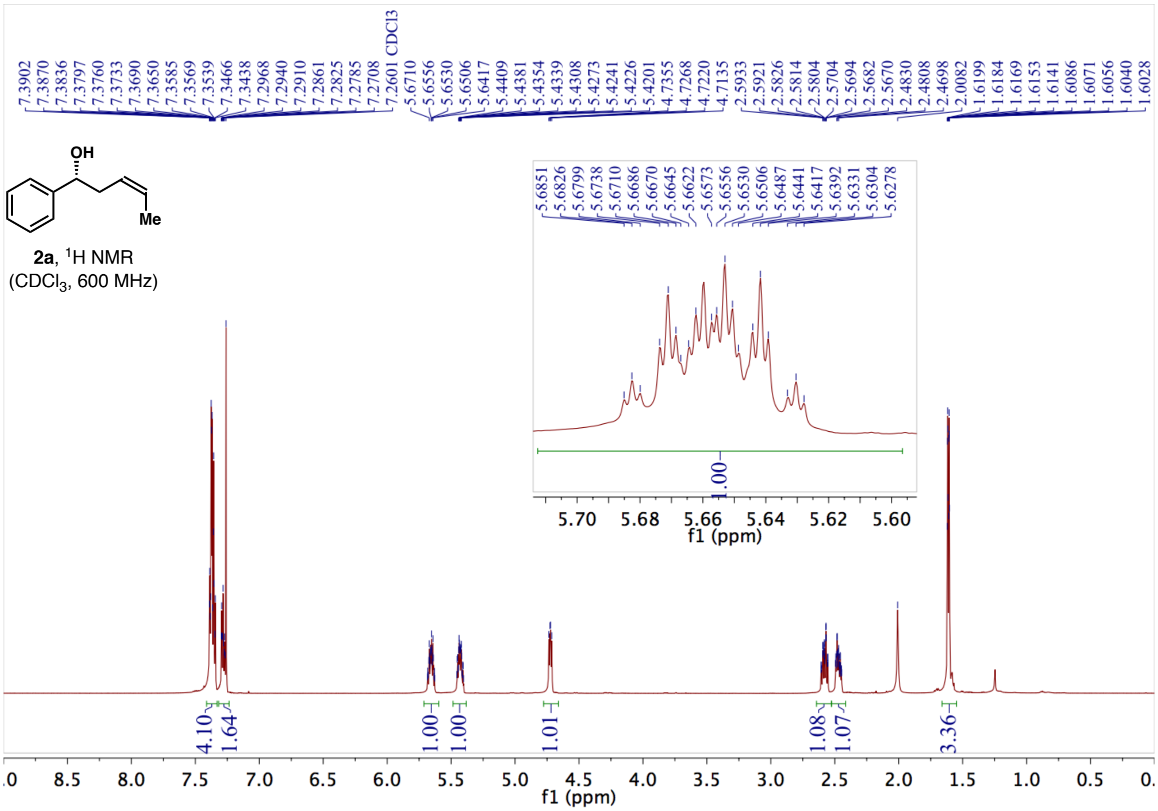

OH
Me
2a, ^{1}H NMR
(CDCl$_3$, 600 MHz)
f1 (ppm)

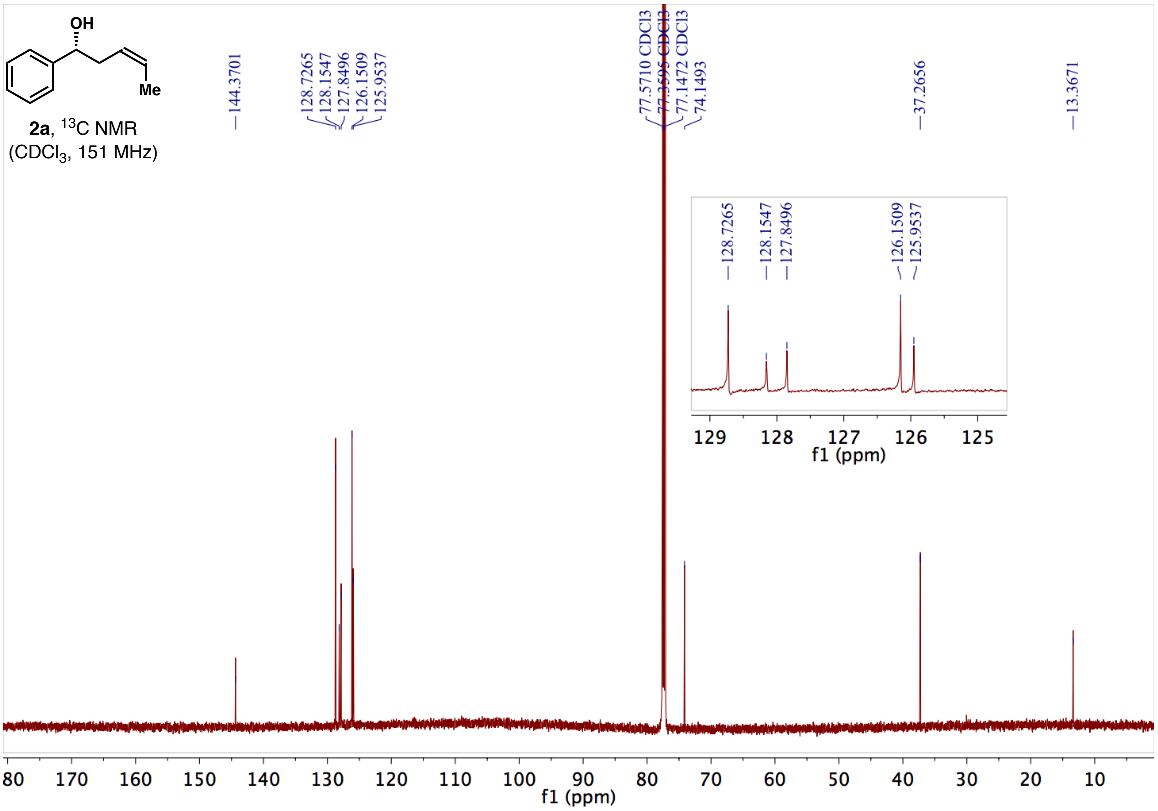

OH
Me
2a, ^{13}C NMR
(CDCl$_3$, 151 MHz)
f1 (ppm)

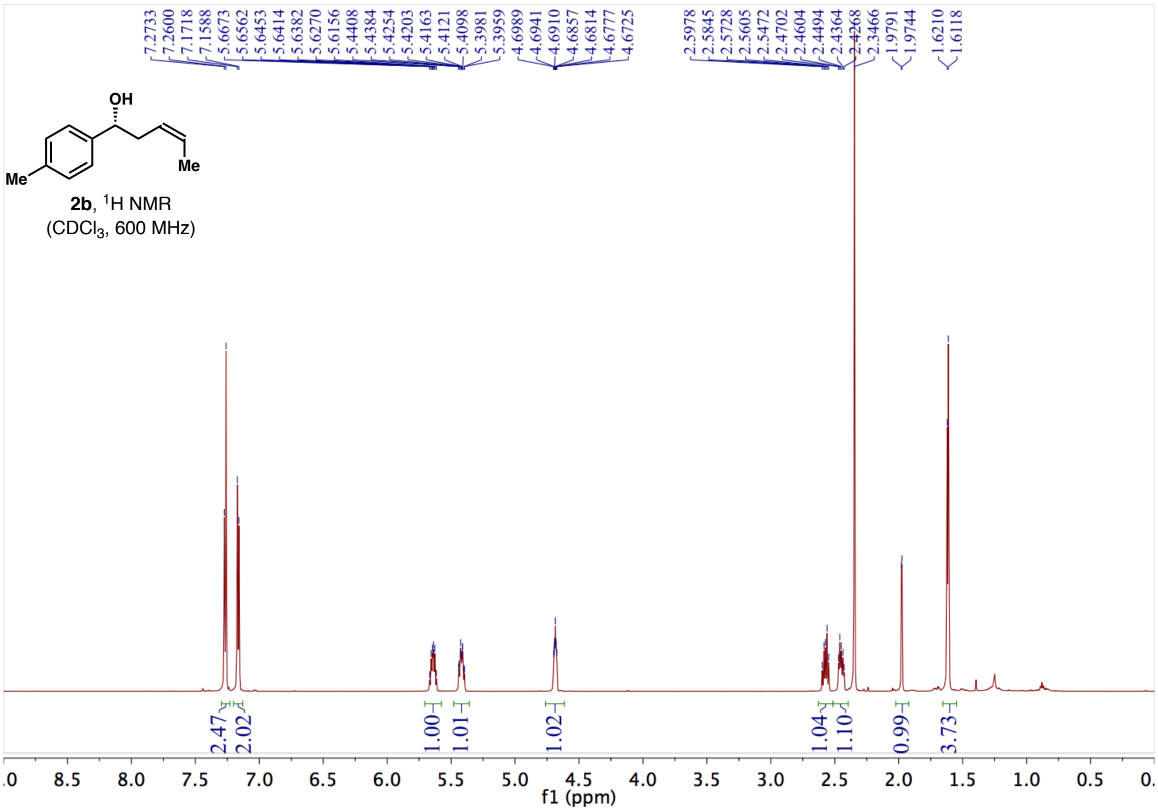
OH
Me
Me
2b, ¹H NMR
(CDCl₃, 600 MHz)
f1 (ppm)

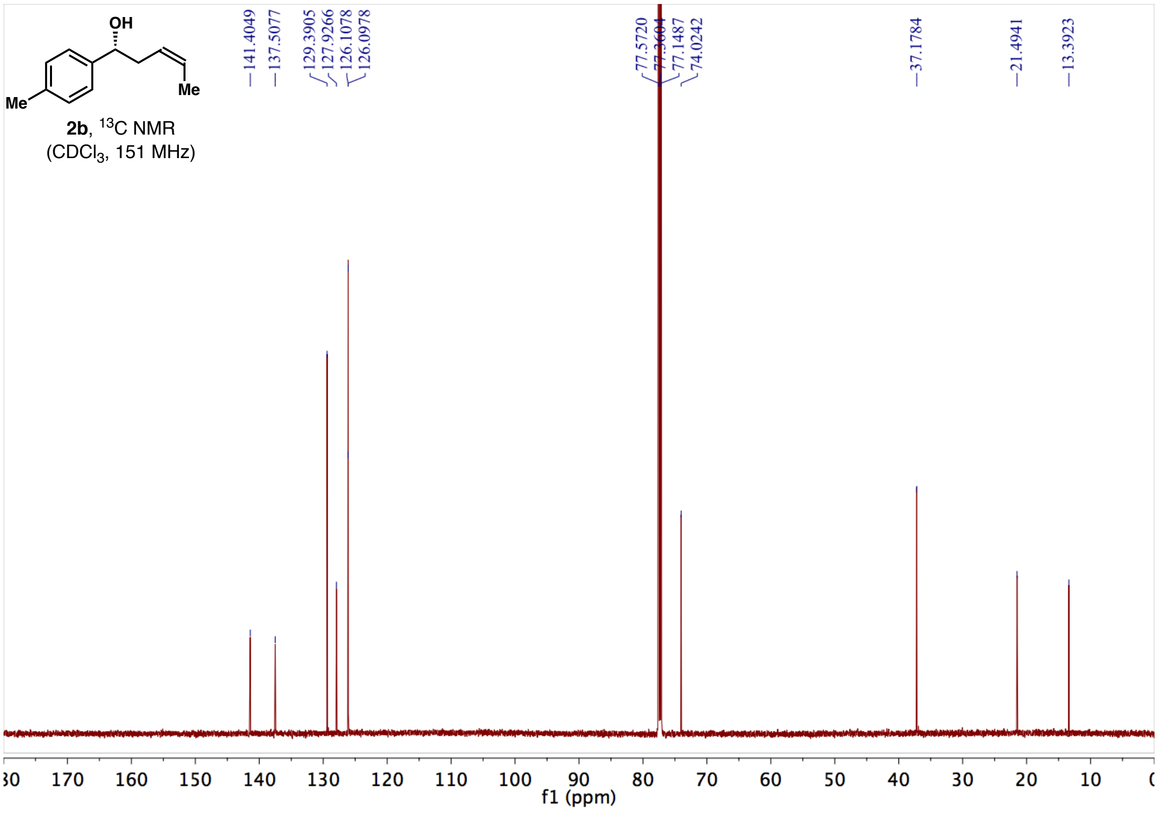
OH
Me
Me
2b, ¹³C NMR
(CDCl₃, 151 MHz)
f1 (ppm)

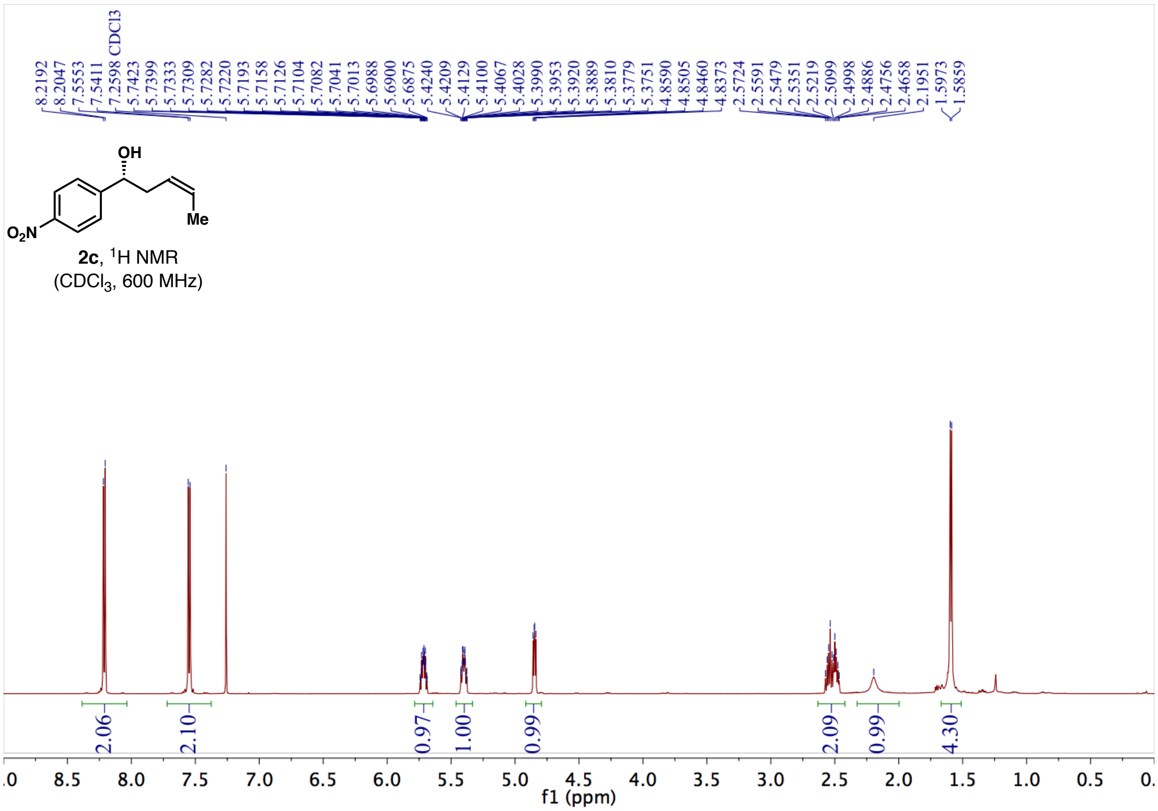
2c, ¹H NMR
(CDCl3, 600 MHz)
f1 (ppm)

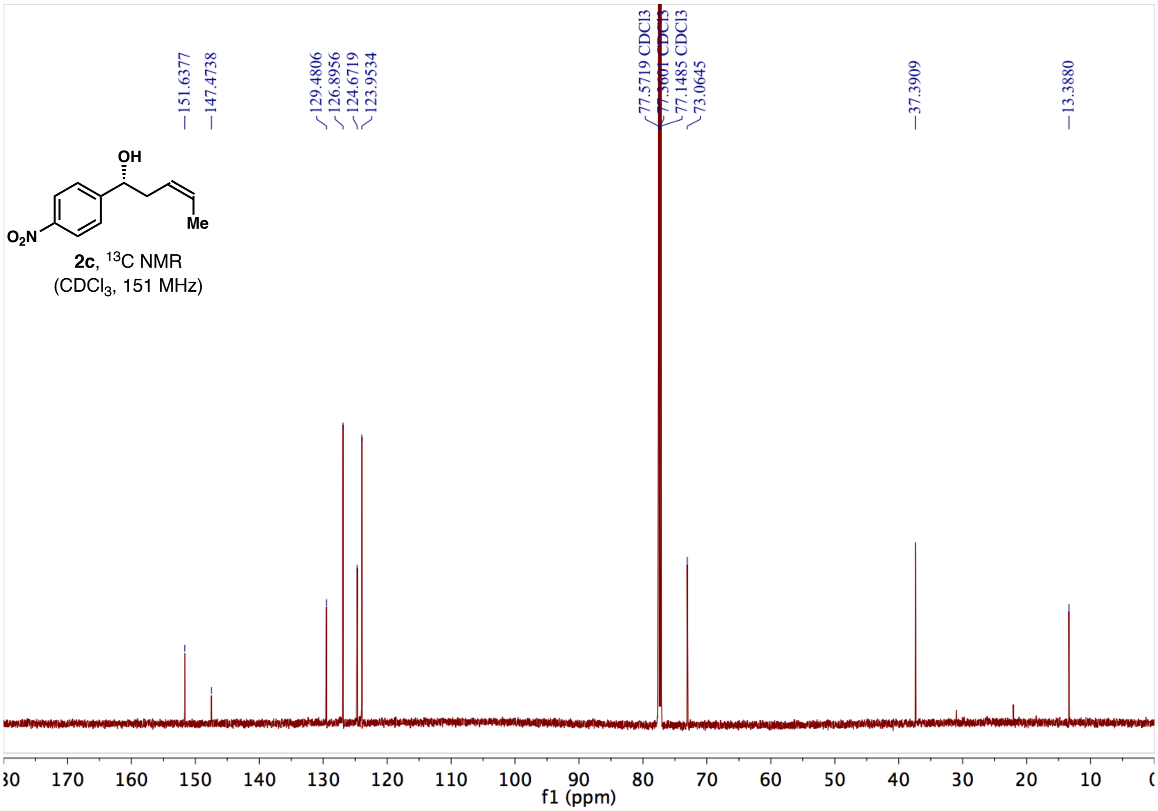
2c, ¹³C NMR
(CDCl3, 151 MHz)
f1 (ppm)

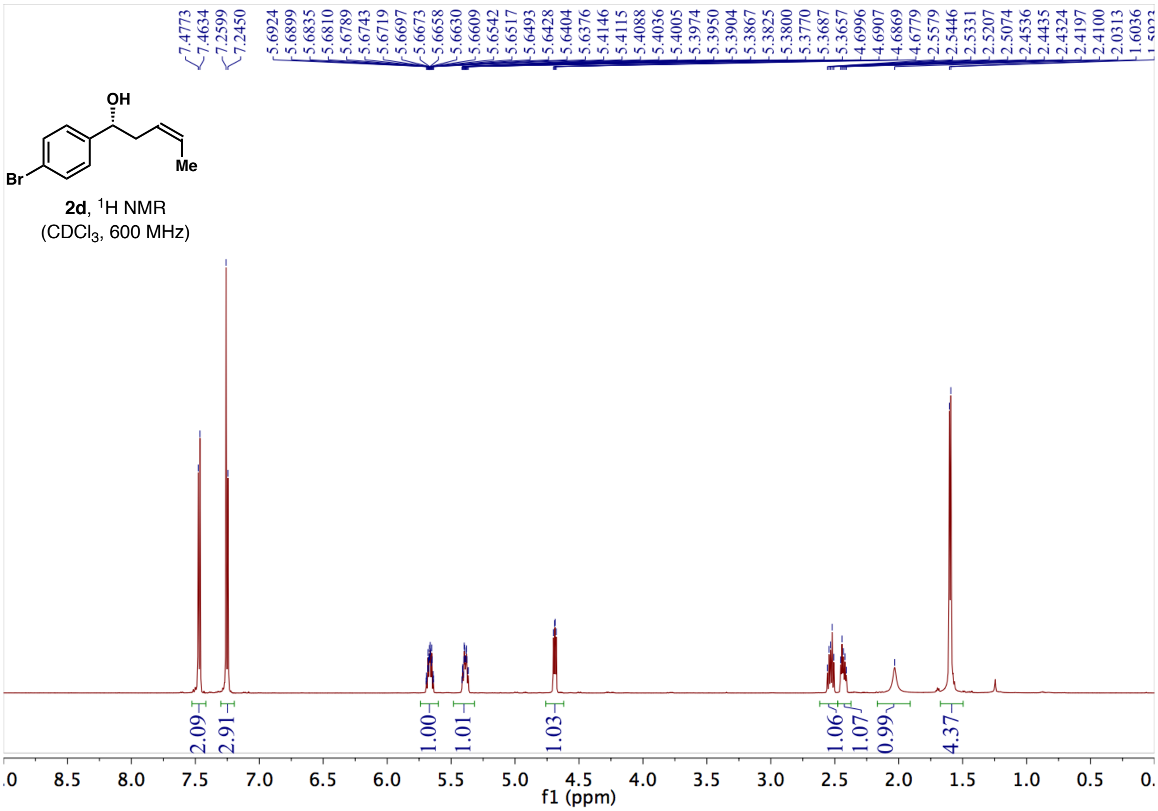
OH
Br
Me
2d, ¹H NMR
(CDCl3, 600 MHz)
f1 (ppm)

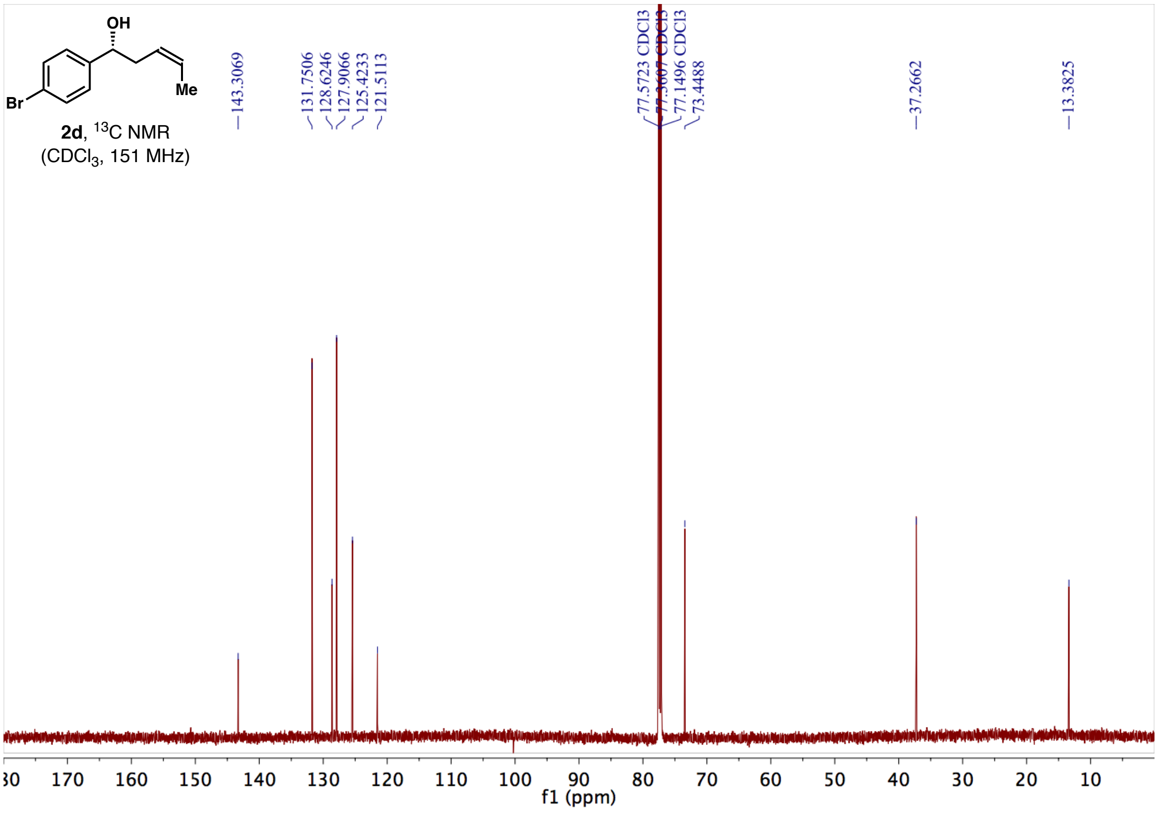
OH
Br
Me
2d, ¹³C NMR
(CDCl3, 151 MHz)
f1 (ppm)

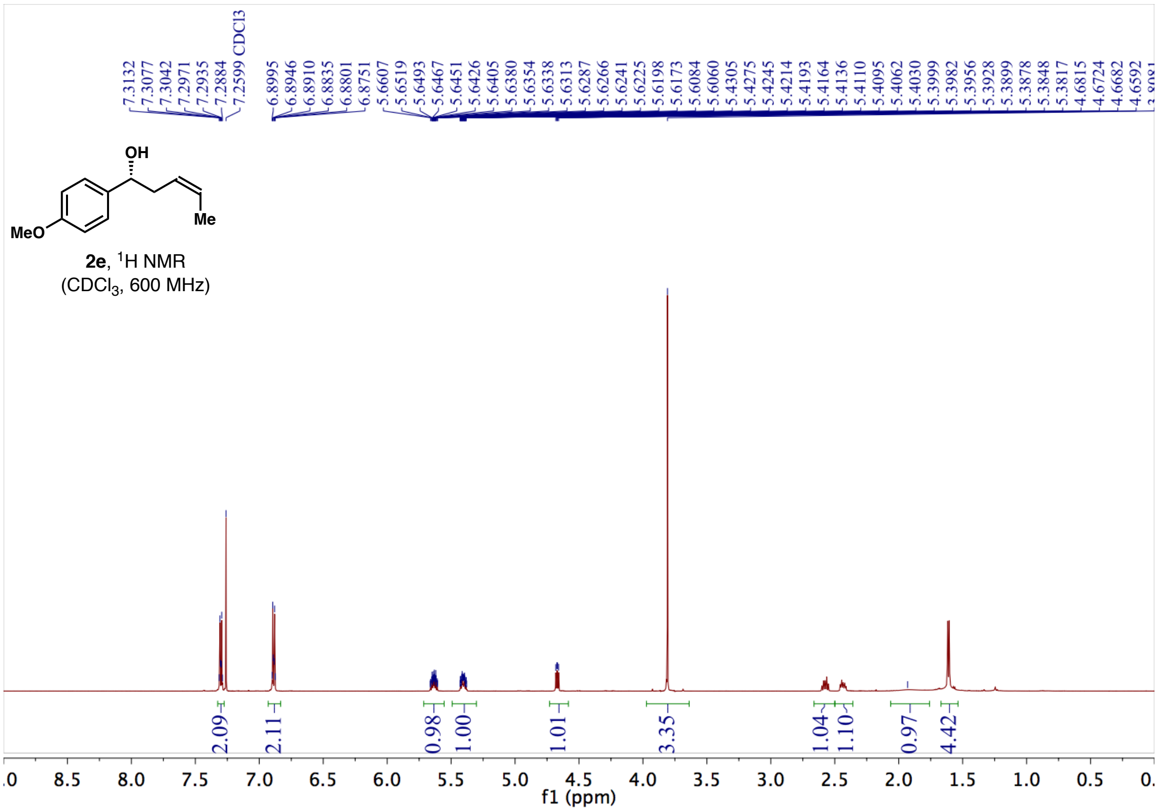
7.3132
7.3077
7.3042
7.2971
7.2935
7.2884
7.2599 CDCl3
6.8995
6.8946
6.8910
6.8835
6.8801
6.8751
5.6607
5.6519
5.6493
5.6467
5.6451
5.6426
5.6405
5.6380
5.6354
5.6338
5.6313
5.6287
5.6266
5.6241
5.6225
5.6198
5.6173
5.6084
5.6060
5.4305
5.4275
5.4245
5.4214
5.4193
5.4164
5.4136
5.4110
5.4095
5.4062
5.4030
5.3999
5.3982
5.3956
5.3928
5.3899
5.3878
5.3848
5.3817
4.6815
4.6724
4.6682
4.6592
OH
MeO
Me
2e, 1H NMR
(CDCl3, 600 MHz)
2.09
2.11
0.98
1.00
1.01
3.35
1.04
1.10
0.97
4.42
8.5
8.0
7.5
7.0
6.5
6.0
5.5
5.0
4.5
4.0
3.5
3.0
2.5
2.0
1.5
1.0
0.5
f1 (ppm)

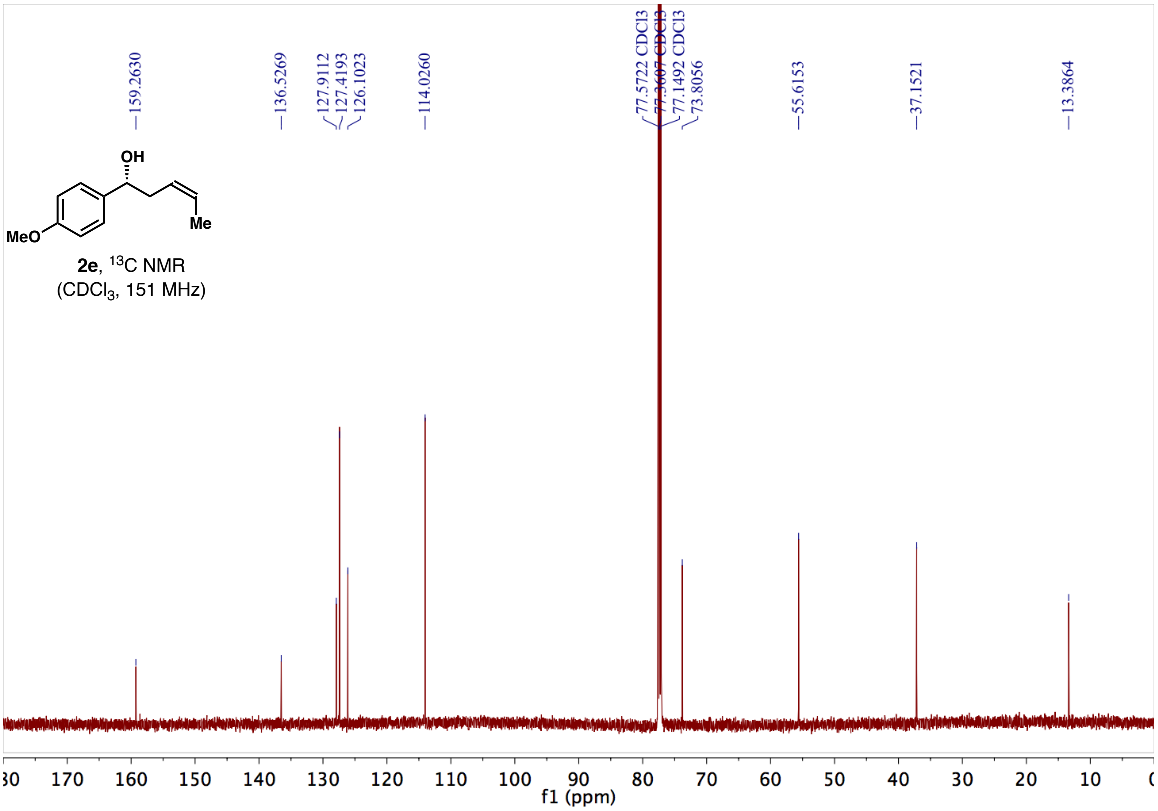
159.2630
136.5269
127.9112
127.4193
126.1023
114.0260
77.5722 CDCl3
77.3607 CDCl3
77.1492 CDCl3
73.8056
55.6153
37.1521
13.3864
OH
MeO
Me
2e, 13C NMR
(CDCl3, 151 MHz)
170
160
150
140
130
120
110
100
90
80
70
60
50
40
30
20
10
f1 (ppm)

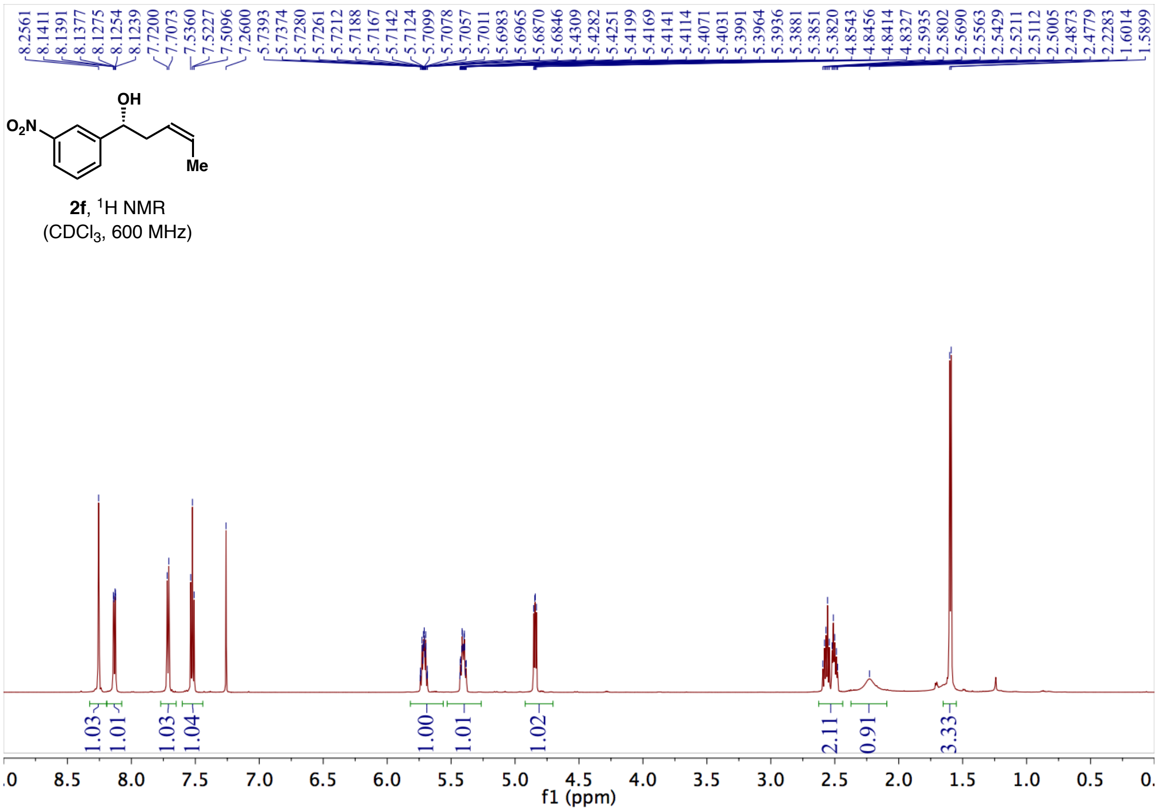
OH
O2N
Me
2f, 1H NMR
(CDCl3, 600 MHz)
8.2561 8.1411 8.1391 8.1377 8.1275 8.1254 8.1239 7.7200 7.7073 7.5360 7.5227 7.5096 7.2600 5.7393 5.7374 5.7280 5.7261 5.7212 5.7188 5.7167 5.7142 5.7124 5.7099 5.7078 5.7057 5.7011 5.6983 5.6965 5.6870 5.6846 5.4309 5.4282 5.4251 5.4199 5.4169 5.4141 5.4114 5.4071 5.4031 5.3991 5.3964 5.3936 5.3881 5.3851 5.3820 4.8543 4.8456 4.8414 4.8327 2.5935 2.5802 2.5690 2.5563 2.5429 2.5211 2.5112 2.5005 2.4873 2.4779 2.2283 1.6014 1.5899
1.03 1.01 1.03 1.04 1.00 1.01 1.02 2.11 0.91 3.33
f1 (ppm)

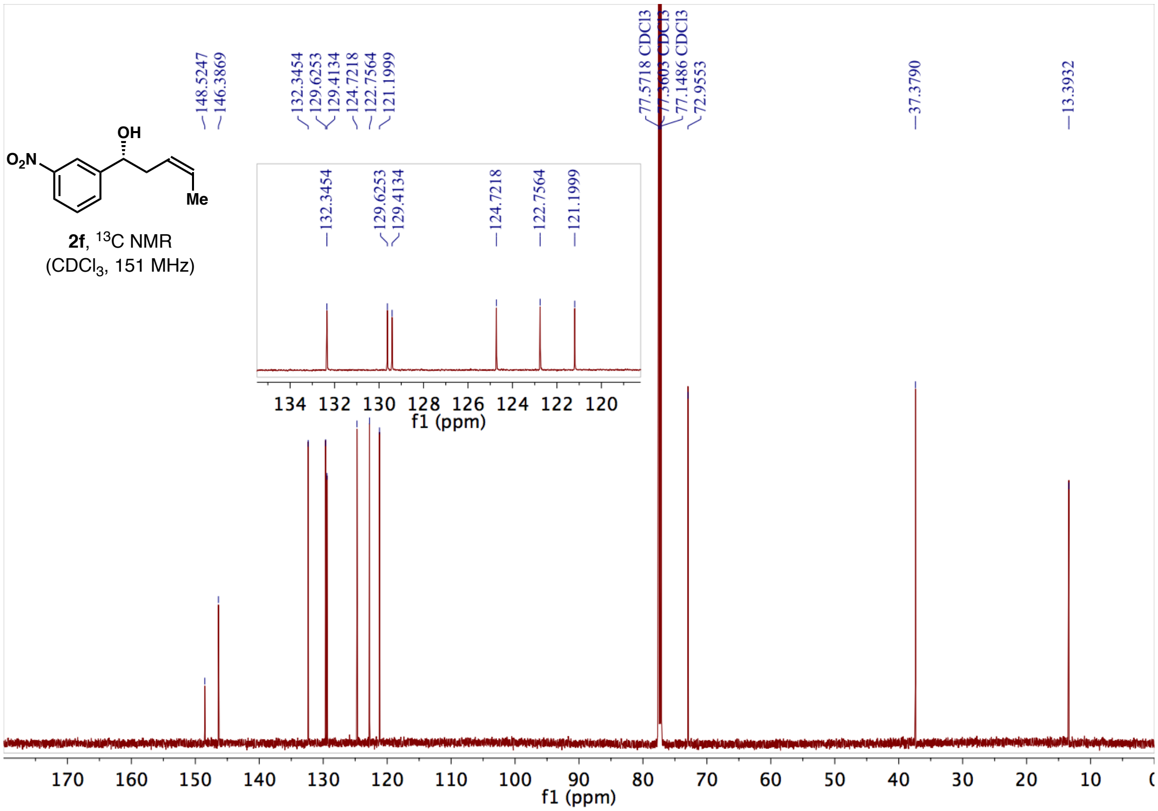
OH
O2N
Me
2f, 13C NMR
(CDCl3, 151 MHz)
148.5247 146.3869 132.3454 129.6253 129.4134 124.7218 122.7564 121.1999 77.5718 CDCl3 77.3603 CDCl3 77.1486 CDCl3 72.9553 37.3790 13.3932
f1 (ppm)

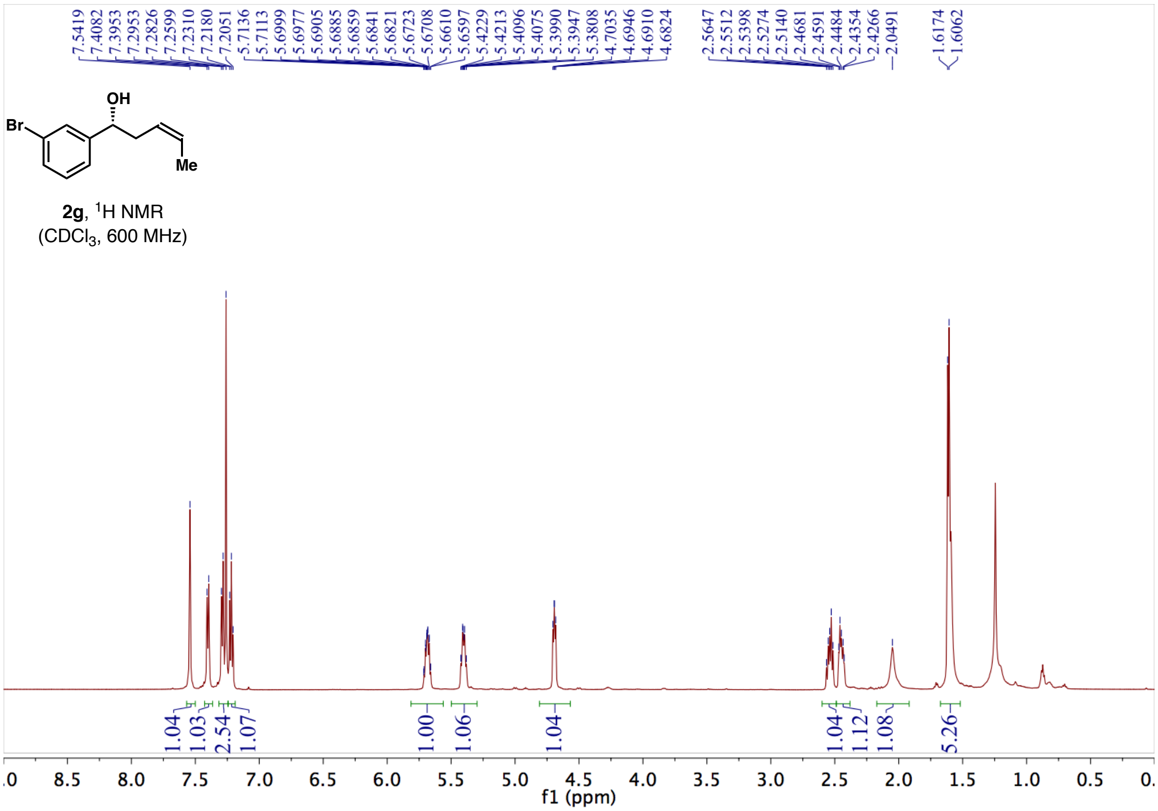
7.5419
7.4082
7.3953
7.2953
7.2826
7.2599
7.2310
7.2180
7.2051
5.7136
5.7113
5.6999
5.6977
5.6905
5.6885
5.6859
5.6841
5.6821
5.6723
5.6708
5.6610
5.6597
5.4229
5.4213
5.4096
5.4075
5.3990
5.3947
5.3808
4.7035
4.6946
4.6910
4.6824
2.5647
2.5512
2.5398
2.5274
2.5140
2.4681
2.4591
2.4484
2.4354
2.4266
2.0491
1.6174
1.6062
OH
Br
Me
2g, 1H NMR
(CDCl3, 600 MHz)
1.04
1.03
2.54
1.07
1.00
1.06
1.04
1.04
1.12
1.08
5.26
f1 (ppm)

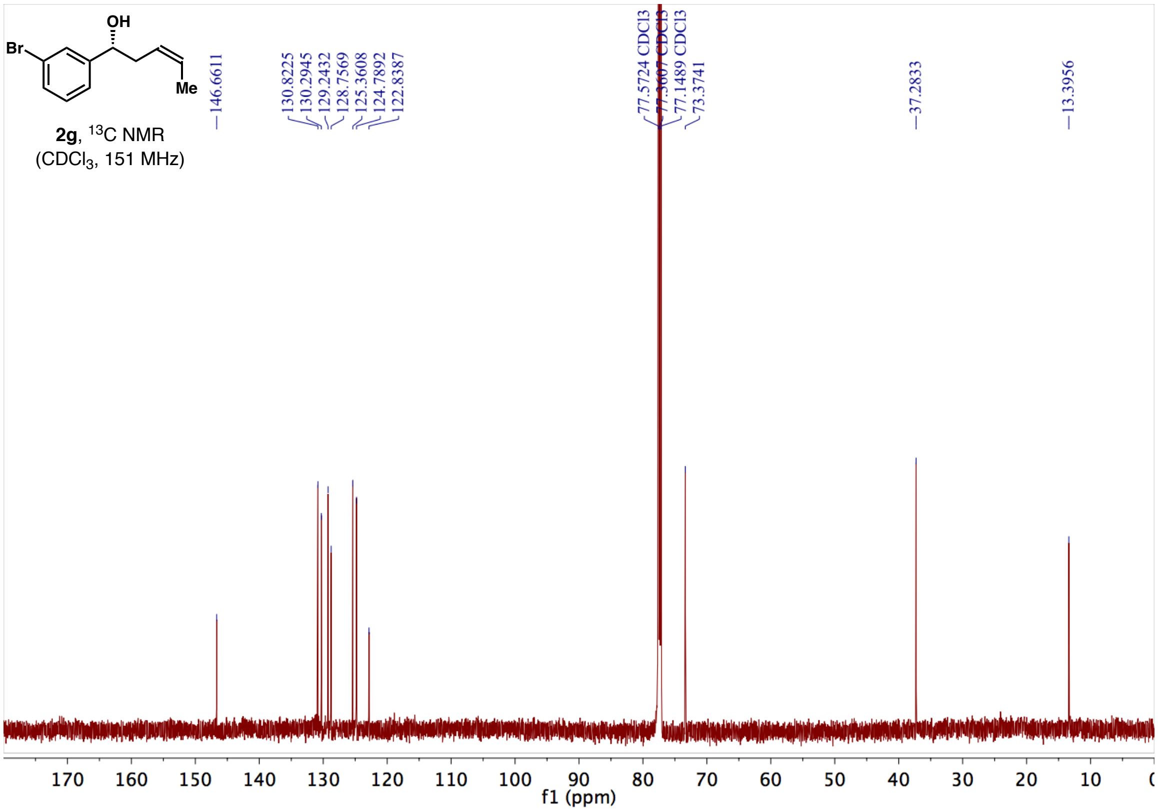
OH
Br
Me
2g, 13C NMR
(CDCl3, 151 MHz)
146.6611
130.8225
130.2945
129.2432
128.7569
125.3608
124.7892
122.8387
77.5724 CDCl3
77.3607 CDCl3
77.1489 CDCl3
73.3741
37.2833
13.3956
f1 (ppm)

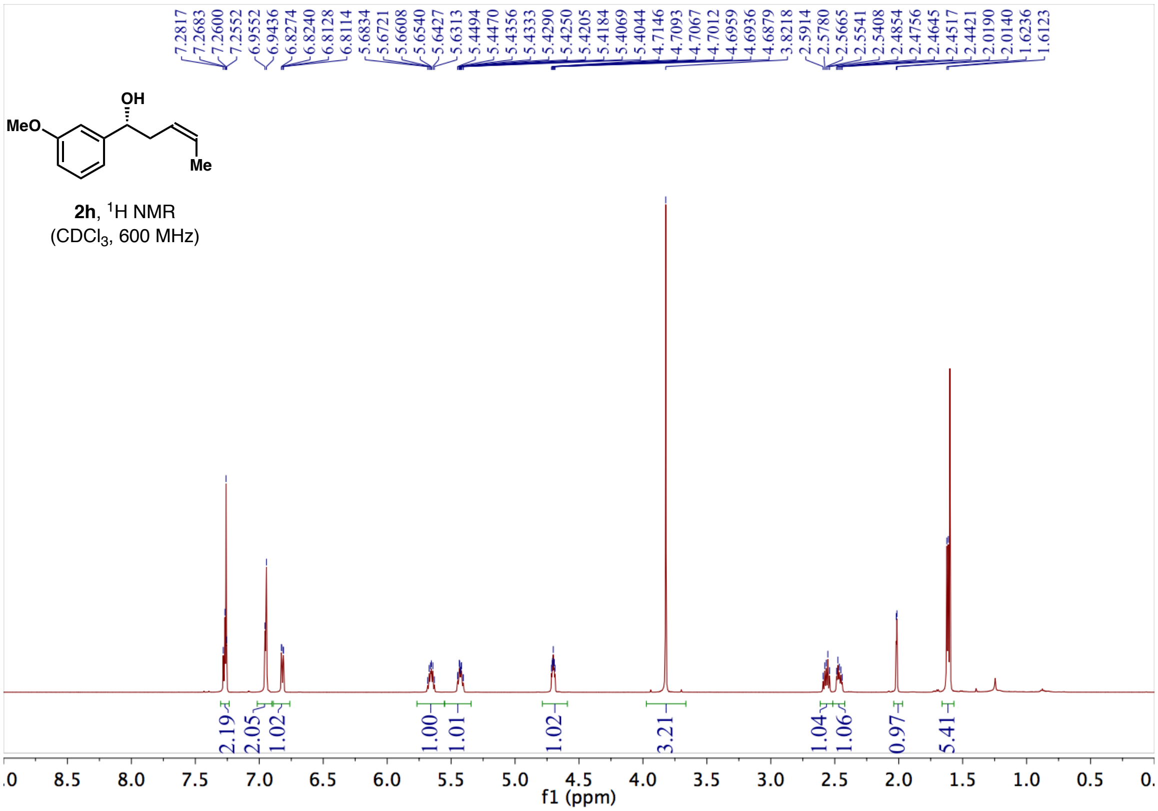
OH
MeO
Me
2h, ¹H NMR
(CDCl₃, 600 MHz)
f1 (ppm)

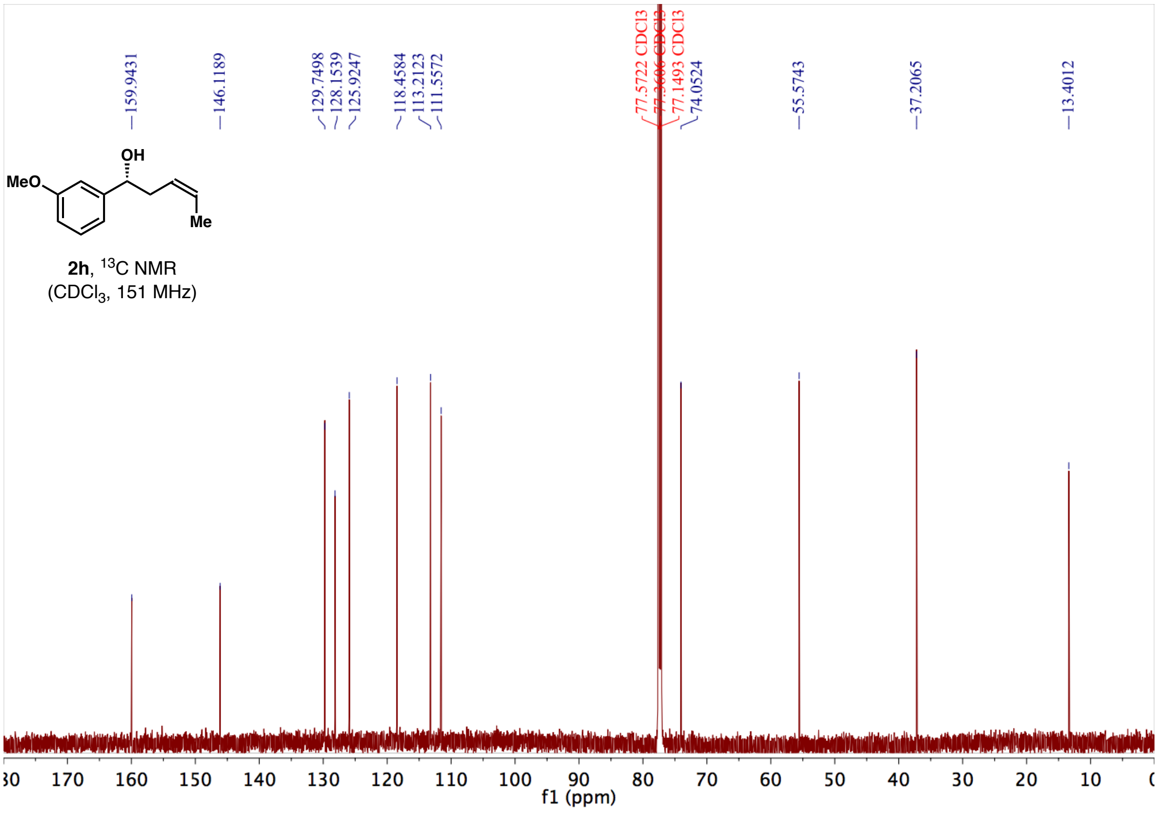
OH
MeO
Me
2h, ¹³C NMR
(CDCl₃, 151 MHz)
f1 (ppm)

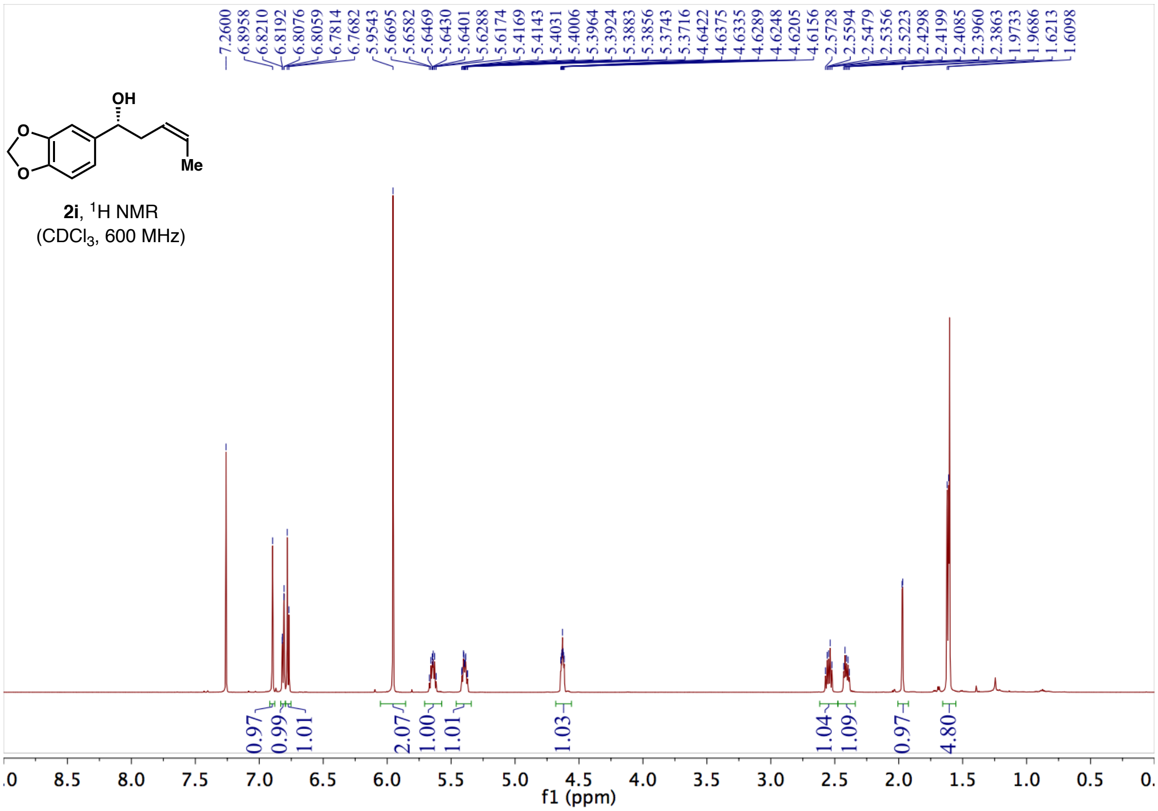
2i, ¹H NMR
(CDCl3, 600 MHz)
f1 (ppm)

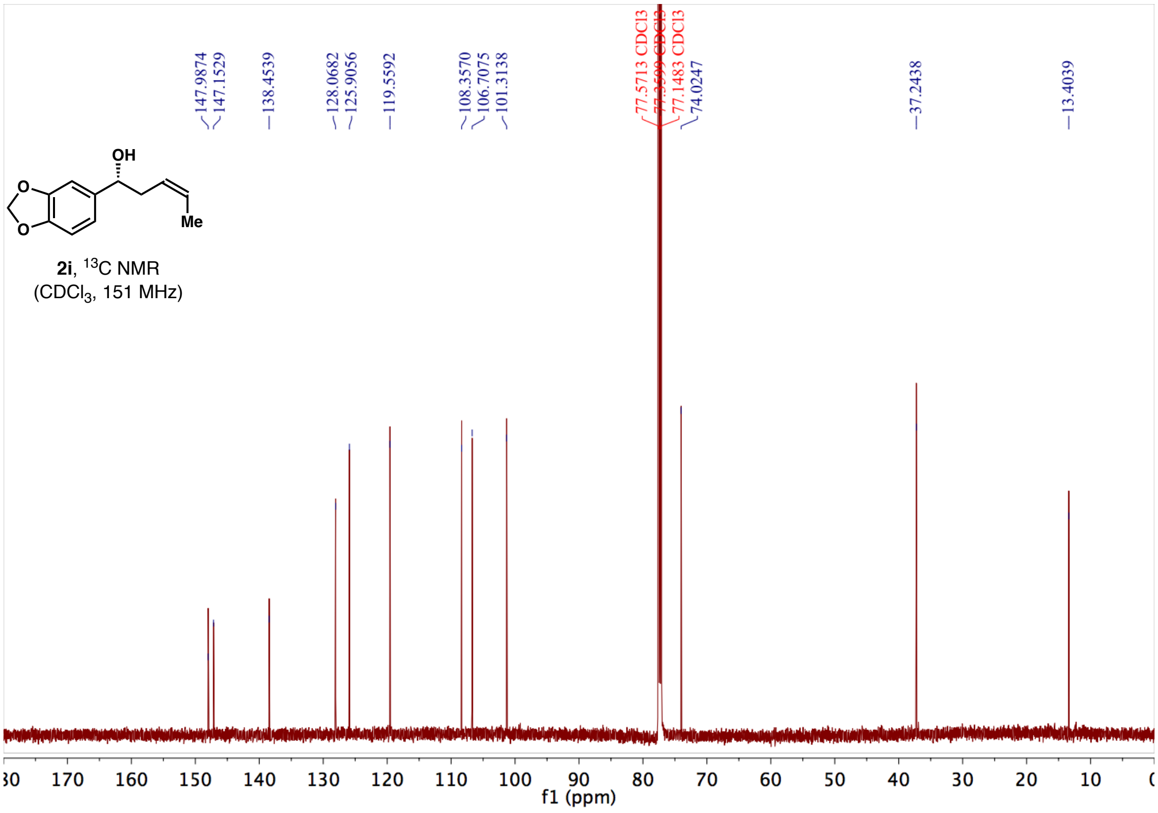
2i, ¹³C NMR
(CDCl3, 151 MHz)
f1 (ppm)

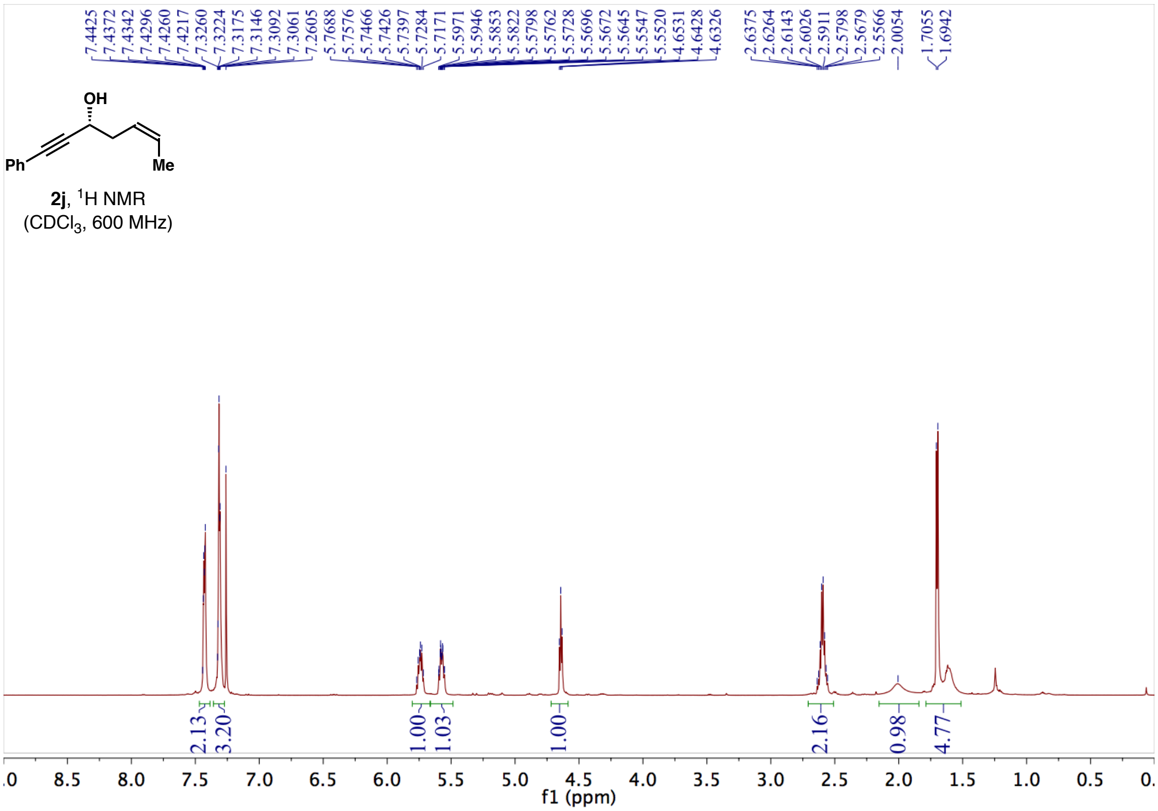
OH
Ph
Me
2j, 1H NMR
(CDCl3, 600 MHz)
7.4425 7.4372 7.4342 7.4296 7.4260 7.4217 7.3260 7.3224 7.3175 7.3146 7.3092 7.3061 7.2605 5.7688 5.7576 5.7466 5.7426 5.7397 5.7284 5.7171 5.5971 5.5946 5.5853 5.5822 5.5798 5.5762 5.5728 5.5696 5.5672 5.5645 5.5547 5.5520 4.6531 4.6428 4.6326 2.6375 2.6264 2.6143 2.6026 2.5911 2.5798 2.5679 2.5566 2.0054 1.7055 1.6942
2.13 3.20 1.00 1.03 1.00 2.16 0.98 4.77
f1 (ppm)

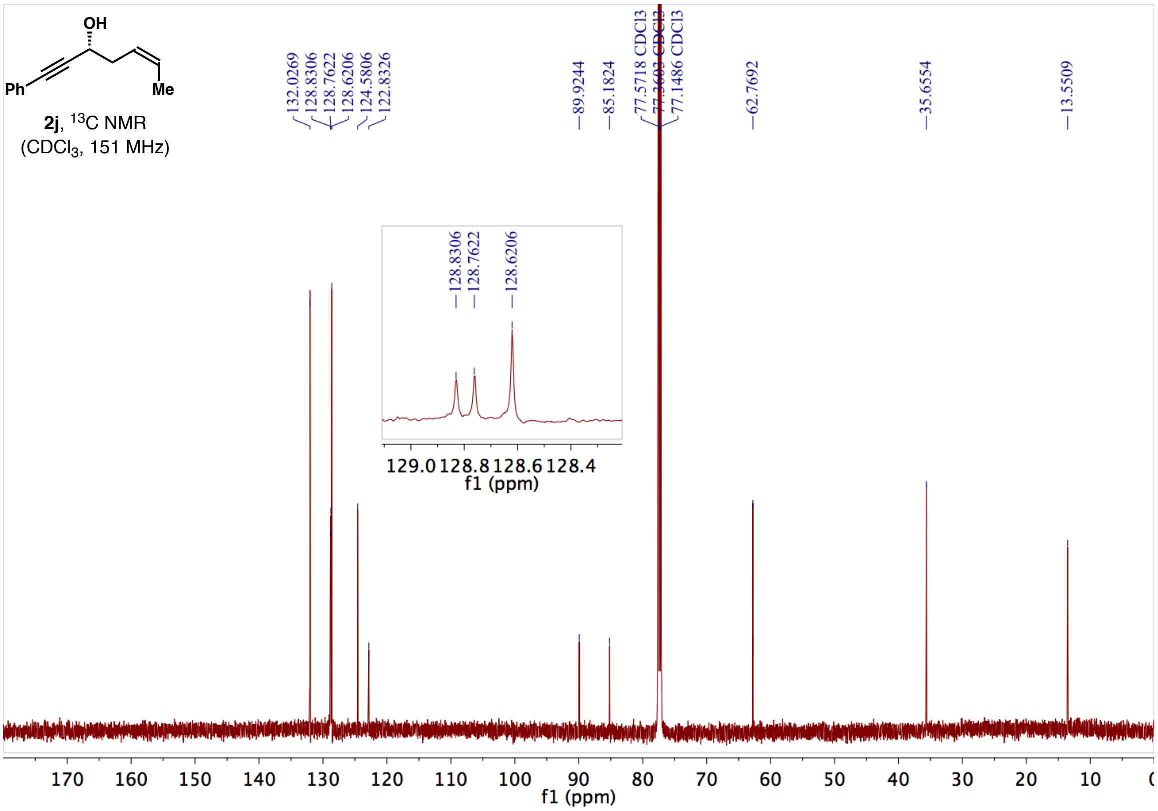
OH
Ph
Me
2j, 13C NMR
(CDCl3, 151 MHz)
132.0269 128.8306 128.7622 128.6206 124.5806 122.8326 89.9244 85.1824 77.5718 CDCl3 77.3603 CDCl3 77.1486 CDCl3 62.7692 35.6554 13.5509
128.8306 128.7622 128.6206
f1 (ppm)

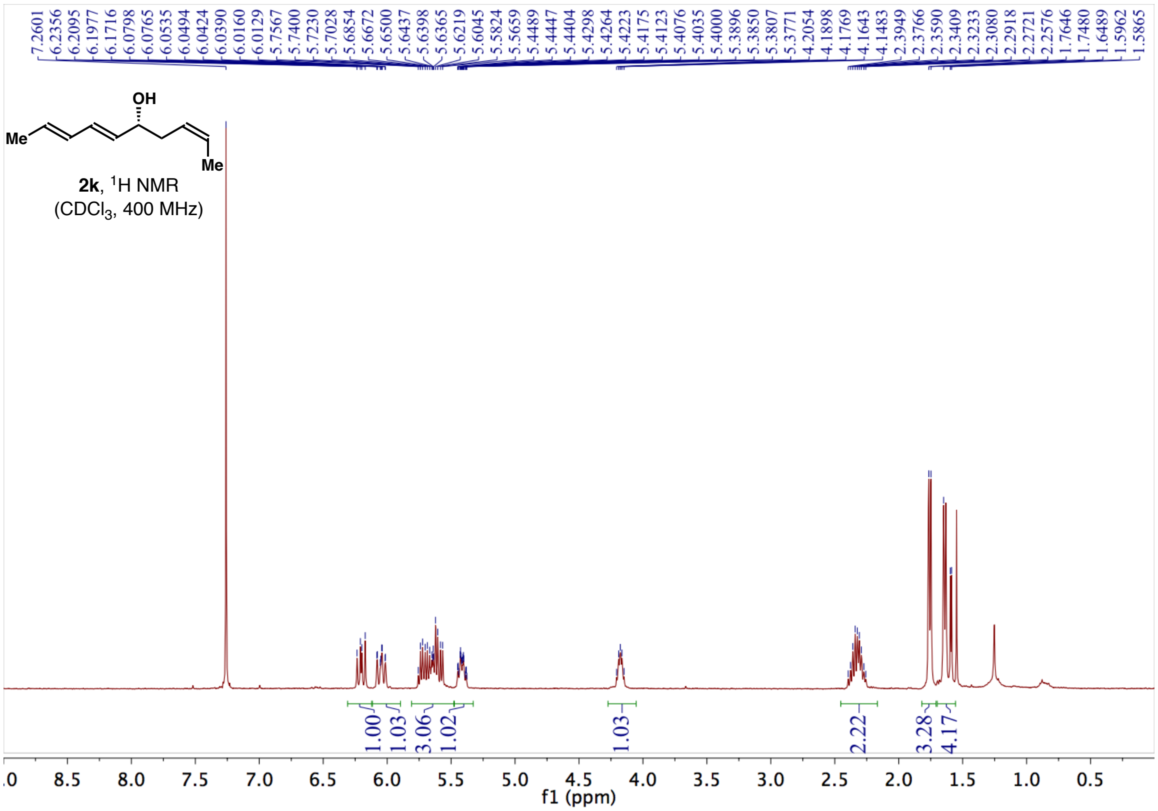
7.2601
6.2356
6.2095
6.1977
6.1716
6.0798
6.0765
6.0535
6.0494
6.0424
6.0390
6.0160
6.0129
5.7567
5.7400
5.7230
5.7028
5.6854
5.6672
5.6500
5.6437
5.6398
5.6365
5.6219
5.6045
5.5824
5.5659
5.4489
5.4447
5.4404
5.4298
5.4264
5.4223
5.4175
5.4123
5.4076
5.4035
5.4000
5.3896
5.3850
5.3807
5.3771
4.2054
4.1898
4.1769
4.1643
4.1483
2.3949
2.3766
2.3590
2.3409
2.3233
2.3080
2.2918
2.2721
2.2576
1.7646
1.7480
1.6489
1.5962
1.5865
OH
Me
Me
2k, 1H NMR
(CDCl3, 400 MHz)
1.00
1.03
3.06
1.02
1.03
2.22
3.28
4.17
f1 (ppm)
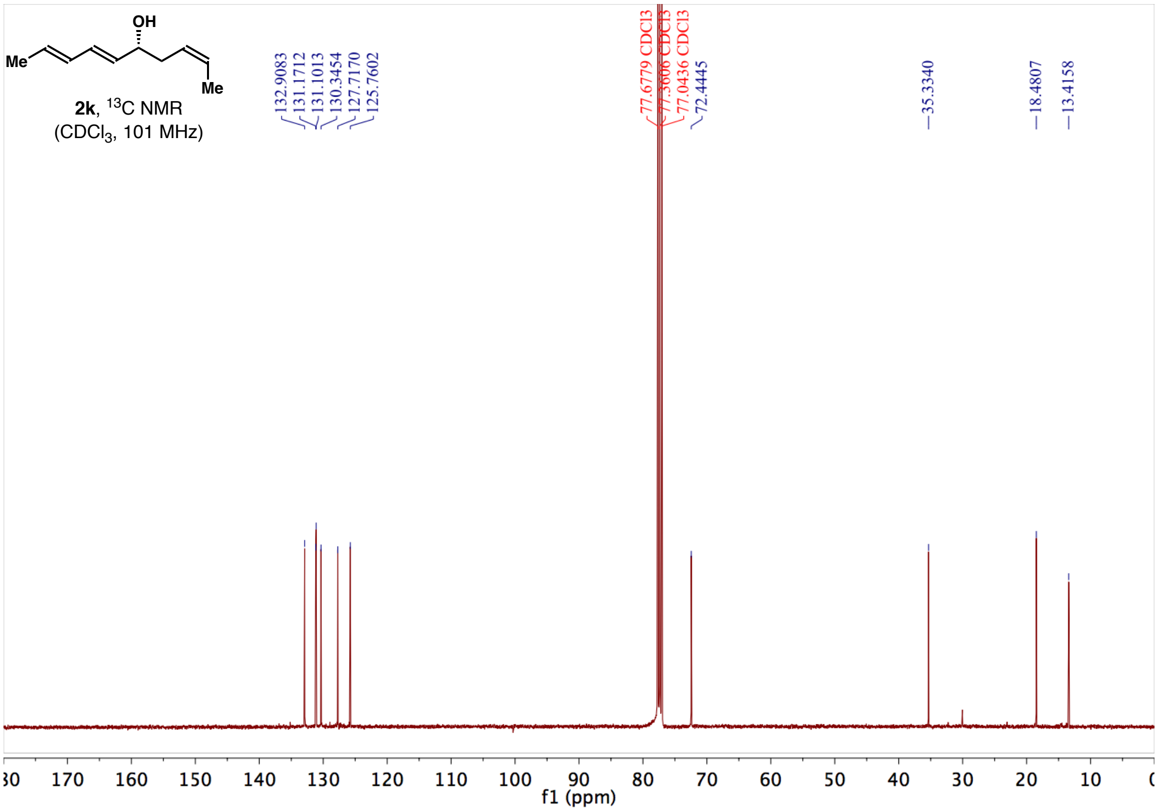
OH
Me
Me
2k, 13C NMR
(CDCl3, 101 MHz)
132.9083
131.1712
131.1013
130.3454
127.7170
125.7602
77.6779 CDCl3
77.3606 CDCl3
77.0436 CDCl3
72.4445
35.3340
18.4807
13.4158
f1 (ppm)

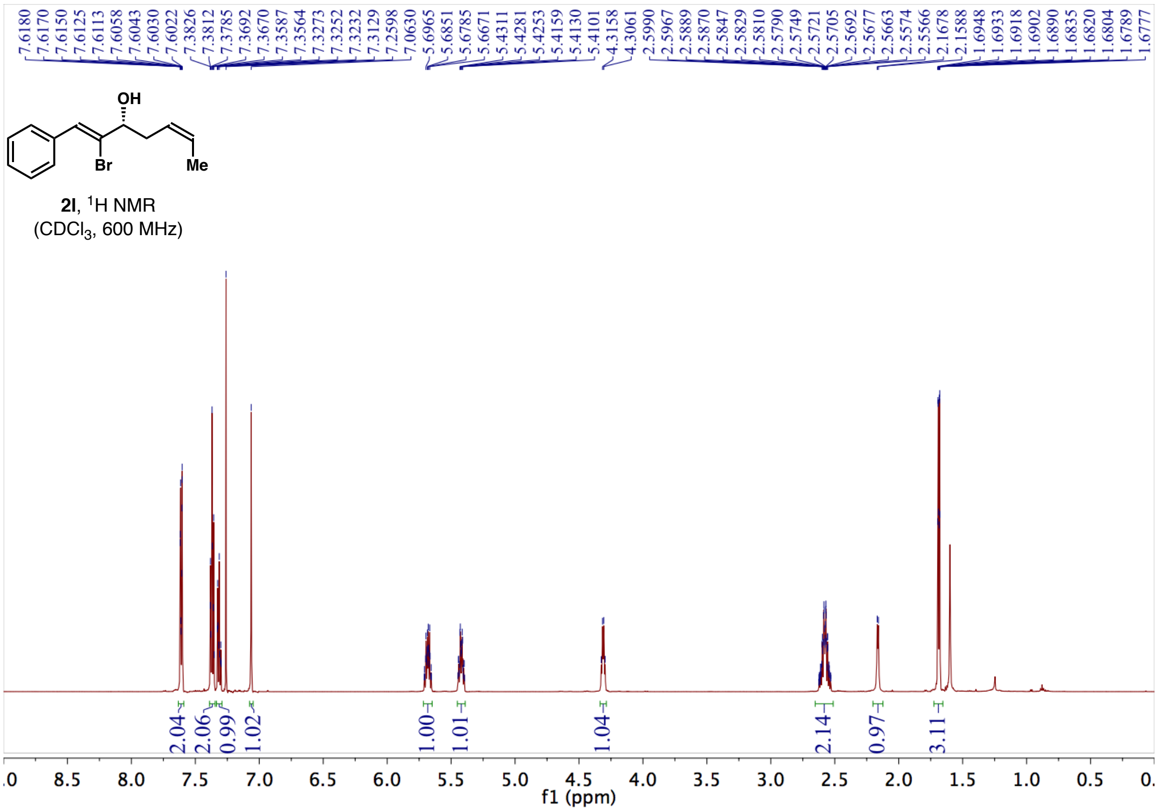

OH
Br
Me
2l, ¹H NMR
(CDCl3, 600 MHz)
f1 (ppm)

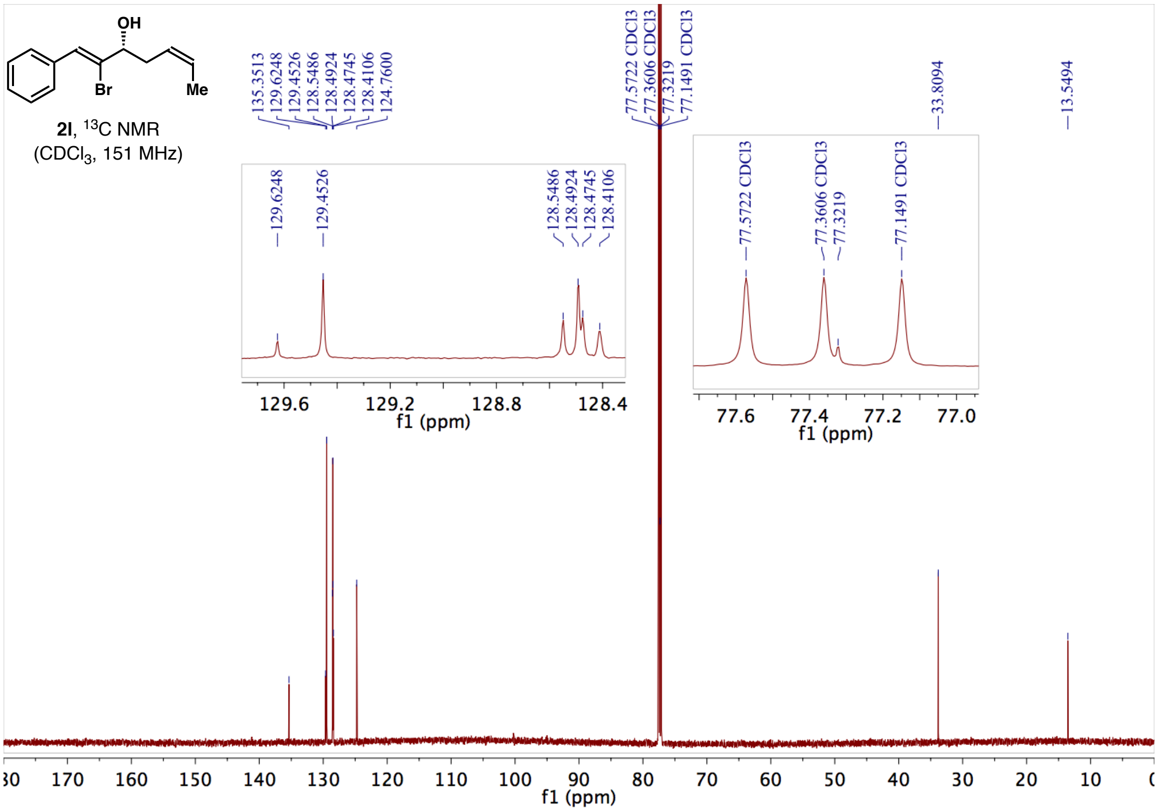

OH
Br
Me
2l, ¹³C NMR
(CDCl3, 151 MHz)
135.3513
129.6248
129.4526
128.5486
128.4924
128.4745
128.4106
124.7600
77.5722 CDCl3
77.3606 CDCl3
77.3219
77.1491 CDCl3
33.8094
13.5494
f1 (ppm)

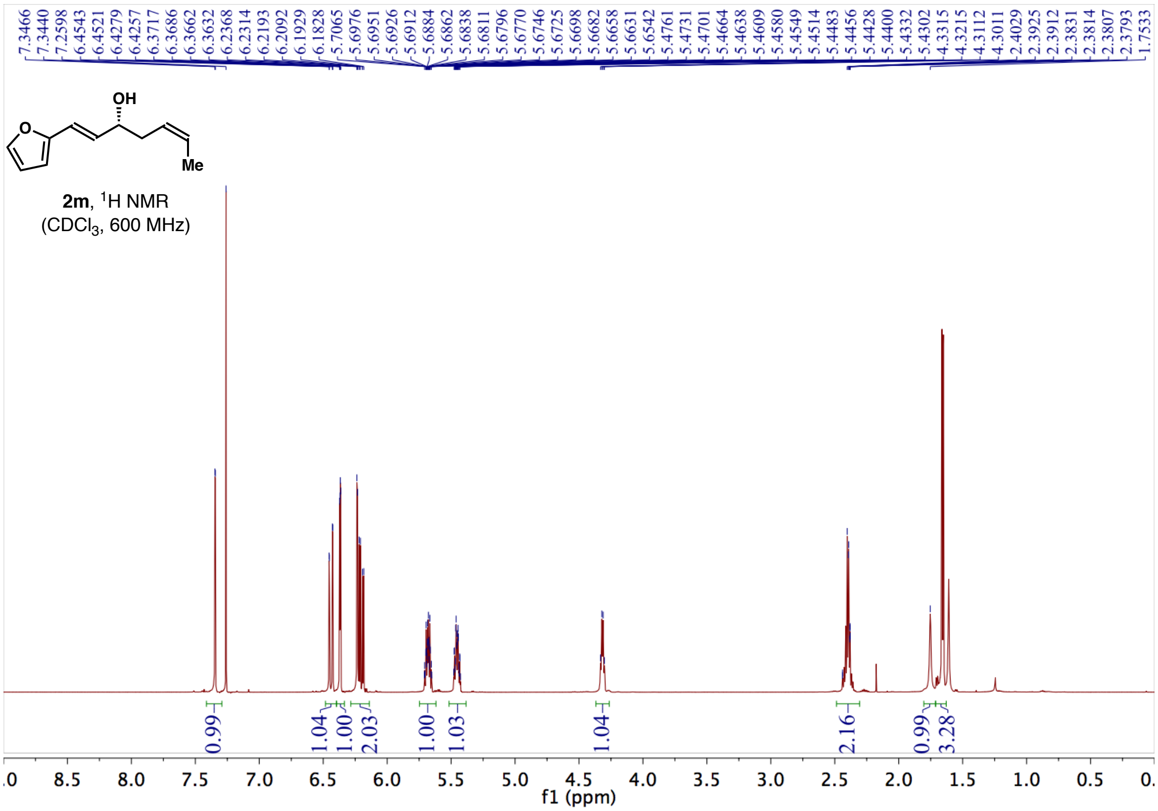

7.3466
7.3440
7.2598
6.4543
6.4521
6.4279
6.4257
6.3717
6.3686
6.3662
6.3632
6.2368
6.2314
6.2193
6.2092
6.1929
6.1828
5.7065
5.6976
5.6951
5.6926
5.6912
5.6884
5.6862
5.6838
5.6811
5.6796
5.6770
5.6746
5.6725
5.6698
5.6682
5.6658
5.6631
5.6542
5.4761
5.4731
5.4701
5.4664
5.4638
5.4609
5.4580
5.4549
5.4514
5.4483
5.4456
5.4428
5.4400
5.4332
5.4302
4.3315
4.3215
4.3112
4.3011
2.4029
2.3925
2.3912
2.3831
2.3814
2.3807
2.3793
1.7533
OH
O
Me
2m, 1H NMR
(CDCl3, 600 MHz)
0.99
1.04
1.00
2.03
1.00
1.03
1.04
2.16
0.99
3.28
.0 8.5 8.0 7.5 7.0 6.5 6.0 5.5 5.0 4.5 4.0 3.5 3.0 2.5 2.0 1.5 1.0 0.5 0.
f1 (ppm)

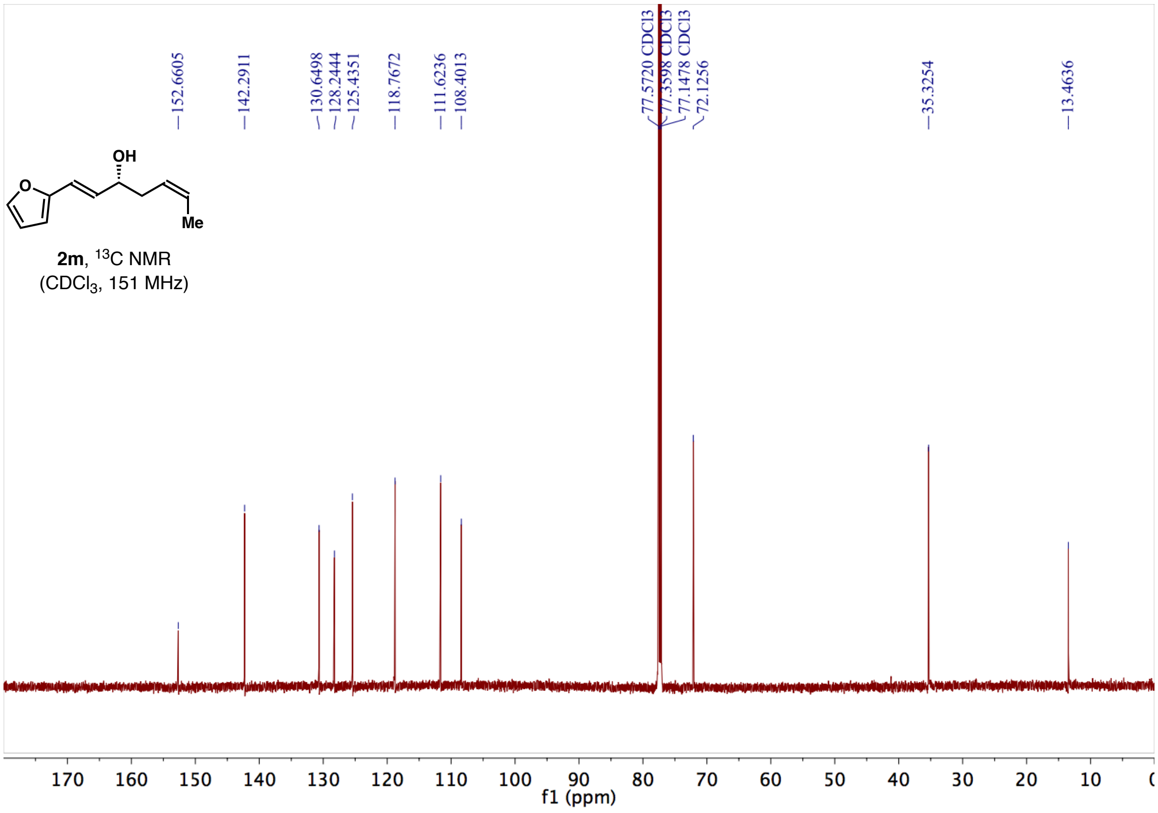

152.6605
142.2911
130.6498
128.2444
125.4351
118.7672
111.6236
108.4013
77.5720 CDCl3
77.3598 CDCl3
77.1478 CDCl3
72.1256
35.3254
13.4636
OH
O
Me
2m, 13C NMR
(CDCl3, 151 MHz)
170 160 150 140 130 120 110 100 90 80 70 60 50 40 30 20 10
f1 (ppm)

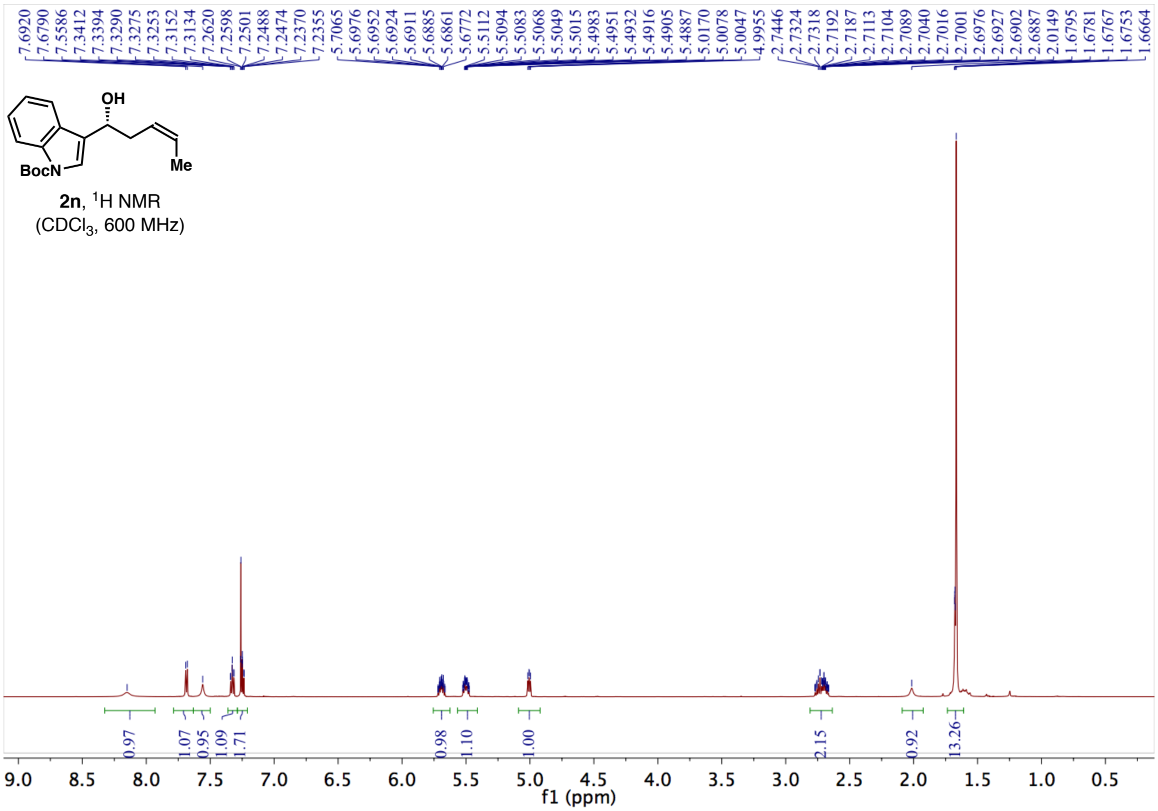
7.6920
7.6790
7.5586
7.3412
7.3394
7.3290
7.3275
7.3253
7.3152
7.3134
7.2620
7.2598
7.2501
7.2488
7.2474
7.2370
7.2355
5.7065
5.6976
5.6952
5.6924
5.6911
5.6885
5.6861
5.6772
5.5112
5.5094
5.5083
5.5068
5.5049
5.5015
5.4983
5.4951
5.4932
5.4916
5.4905
5.4887
5.0170
5.0078
5.0047
4.9955
2.7446
2.7324
2.7318
2.7192
2.7187
2.7113
2.7104
2.7089
2.7040
2.7016
2.7001
2.6976
2.6927
2.6902
2.6887
2.0149
1.6795
1.6781
1.6767
1.6753
1.6664
OH
BocN
Me
2n, 1H NMR
(CDCl3, 600 MHz)
0.97
1.07
0.95
1.09
1.71
0.98
1.10
1.00
2.15
0.92
13.26
9.0 8.5 8.0 7.5 7.0 6.5 6.0 5.5 5.0 4.5 4.0 3.5 3.0 2.5 2.0 1.5 1.0 0.5
f1 (ppm)

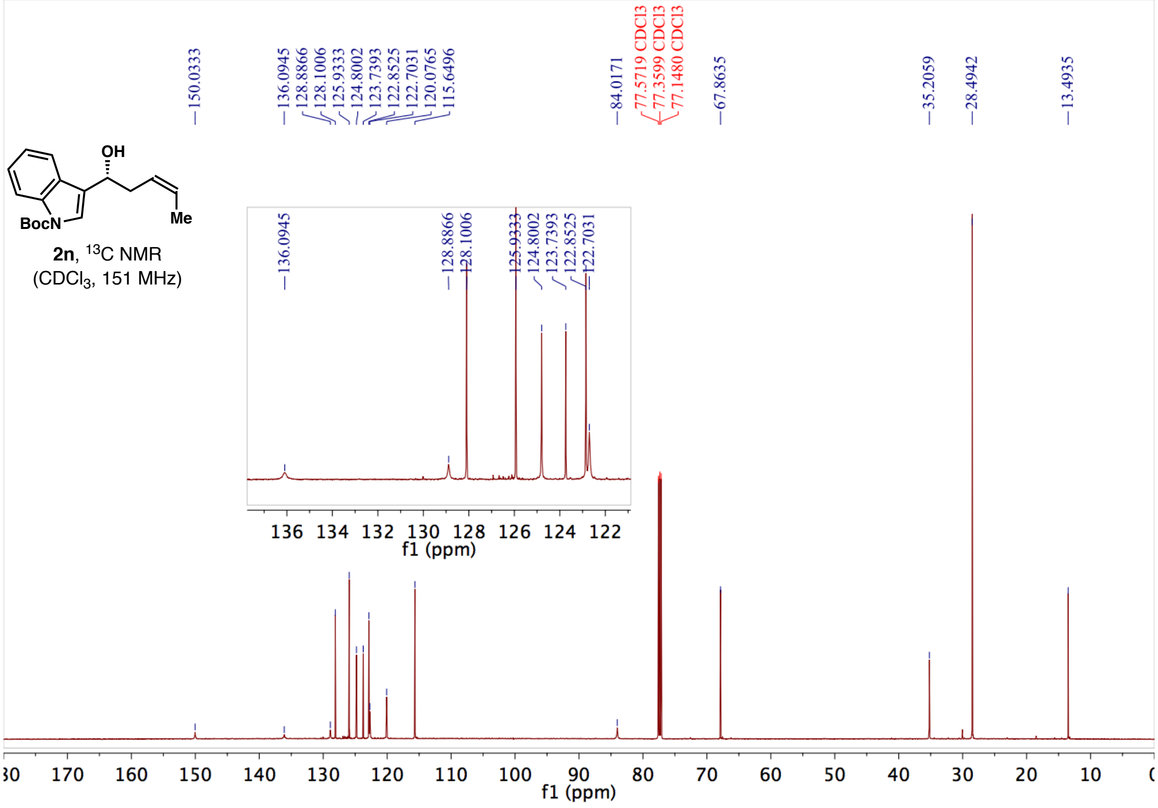
150.0333
136.0945
128.8866
128.1006
125.9333
124.8002
123.7393
122.8525
122.7031
120.0765
115.6496
84.0171
77.5719 CDCl3
77.3599 CDCl3
77.1480 CDCl3
67.8635
35.2059
28.4942
13.4935
OH
BocN
Me
2n, 13C NMR
(CDCl3, 151 MHz)
136.0945
128.8866
128.1006
125.9333
124.8002
123.7393
122.8525
122.7031
136 134 132 130 128 126 124 122
f1 (ppm)
170 160 150 140 130 120 110 100 90 80 70 60 50 40 30 20 10
f1 (ppm)

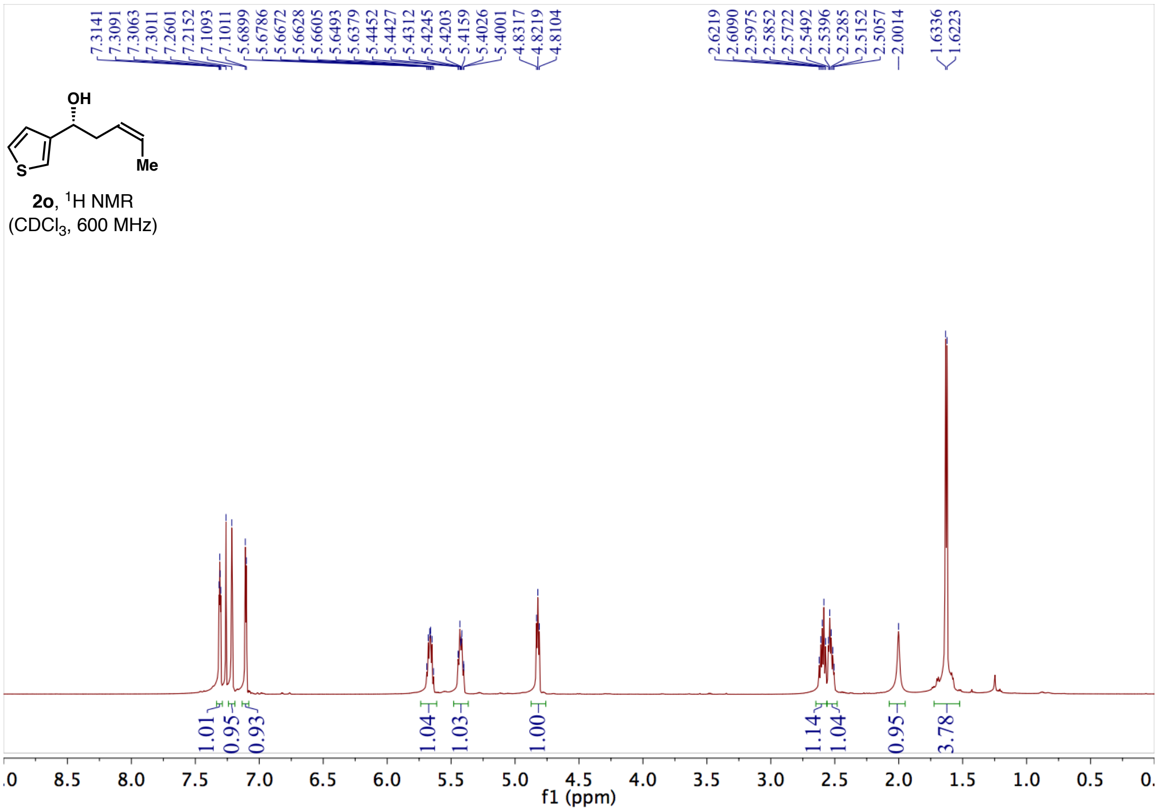
7.3141 7.3091 7.3063 7.3011 7.2601 7.2152 7.1093 7.1011 5.6899 5.6786 5.6672 5.6628 5.6605 5.6493 5.6379 5.4452 5.4427 5.4312 5.4245 5.4203 5.4159 5.4026 5.4001 4.8317 4.8219 4.8104
2.6219 2.6090 2.5975 2.5852 2.5722 2.5492 2.5396 2.5285 2.5152 2.5057 2.0014 1.6336 1.6223
OH
S
Me
2o, 1H NMR
(CDCl3, 600 MHz)
1.01 0.95 0.93 1.04 1.03 1.00 1.14 1.04 0.95 3.78
f1 (ppm)

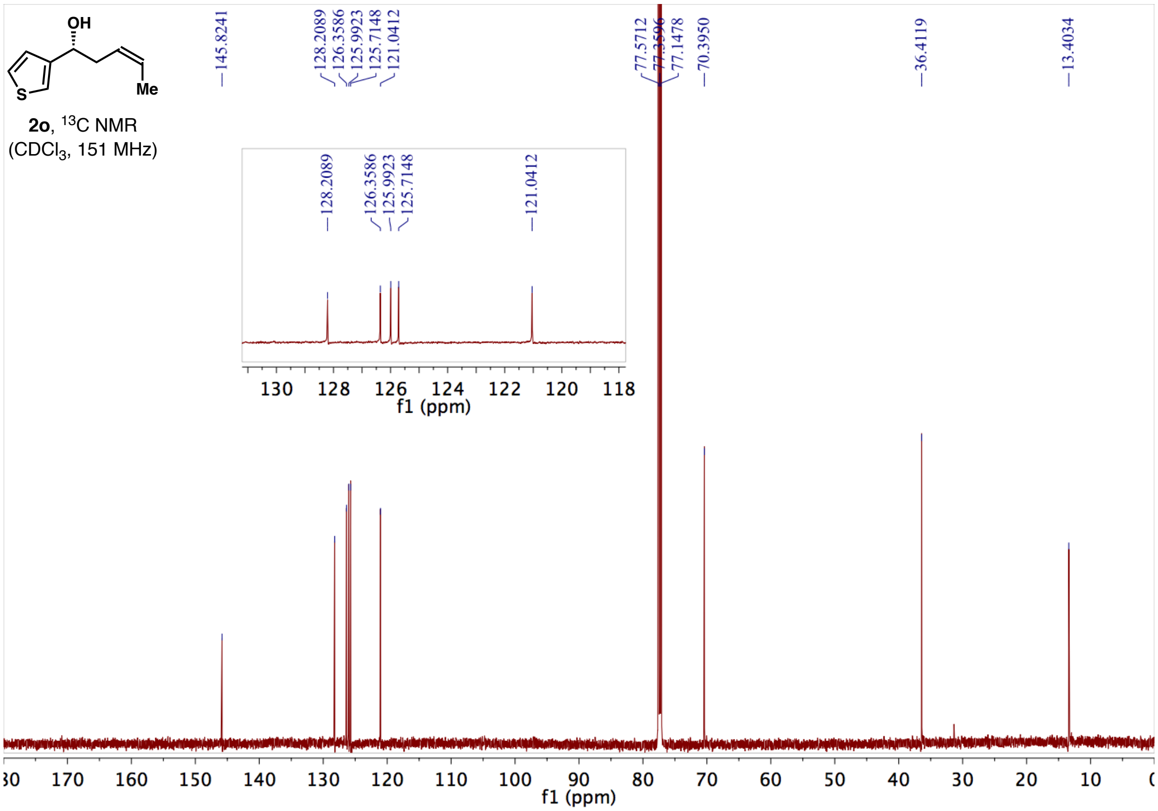
145.8241
128.2089 126.3586 125.9923 125.7148 121.0412
77.5712 77.3596 77.1478 70.3950
36.4119
13.4034
OH
S
Me
2o, 13C NMR
(CDCl3, 151 MHz)
128.2089 126.3586 125.9923 125.7148 121.0412
f1 (ppm)

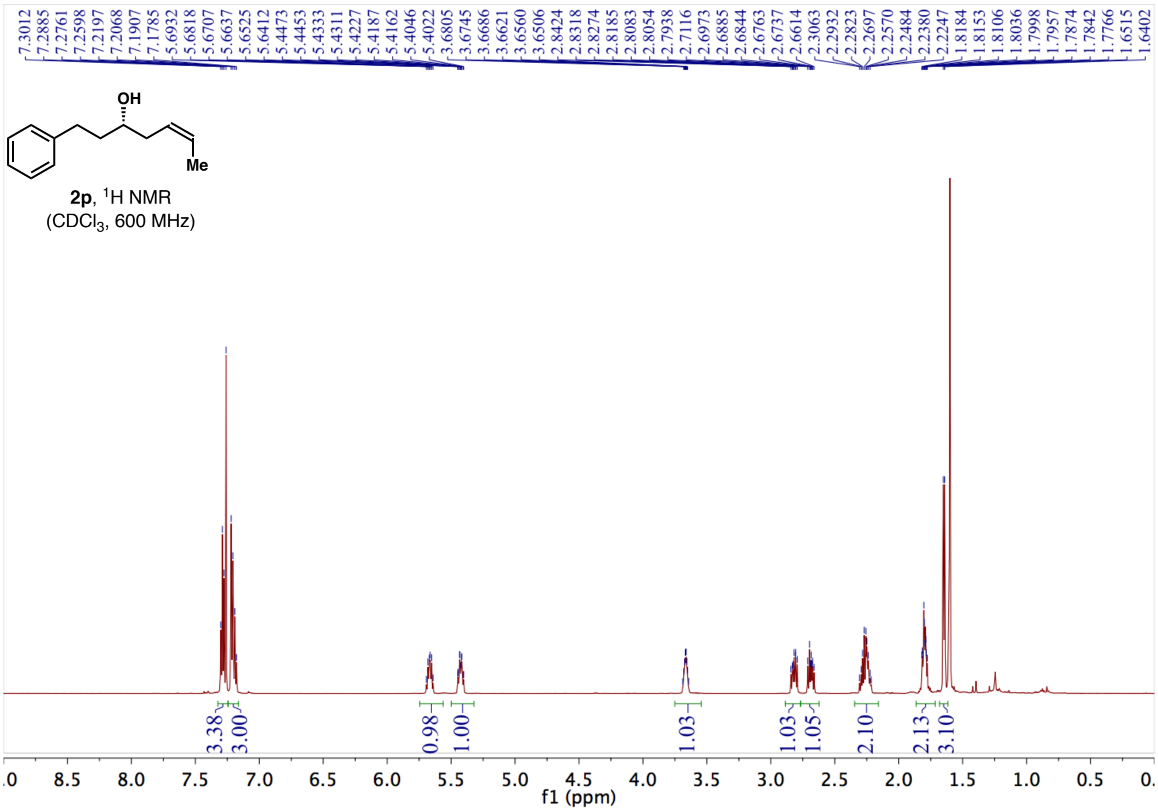
OH
Me
2p, ¹H NMR
(CDCl3, 600 MHz)
f1 (ppm)

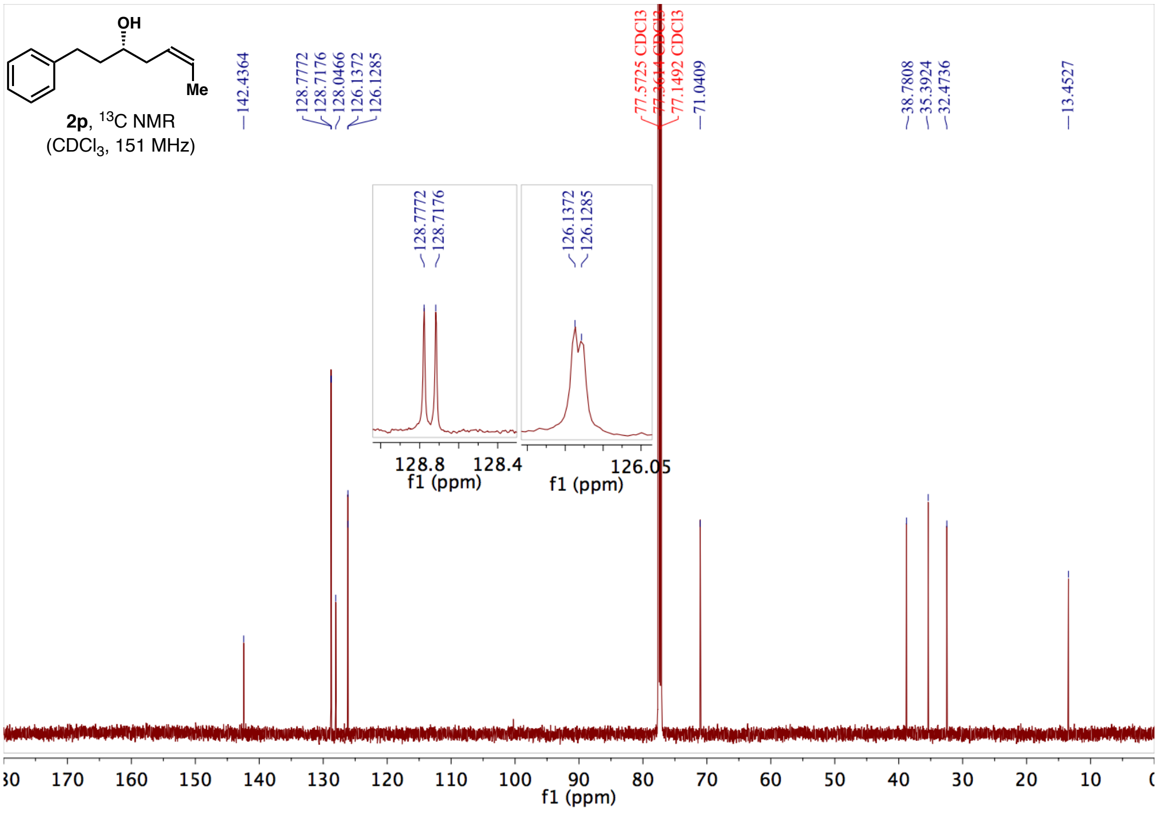
OH
Me
2p, ¹³C NMR
(CDCl3, 151 MHz)
142.4364
128.7772
128.7176
128.0466
126.1372
126.1285
77.5725 CDCl3
77.1492 CDCl3
71.0409
38.7808
35.3924
32.4736
13.4527
f1 (ppm)

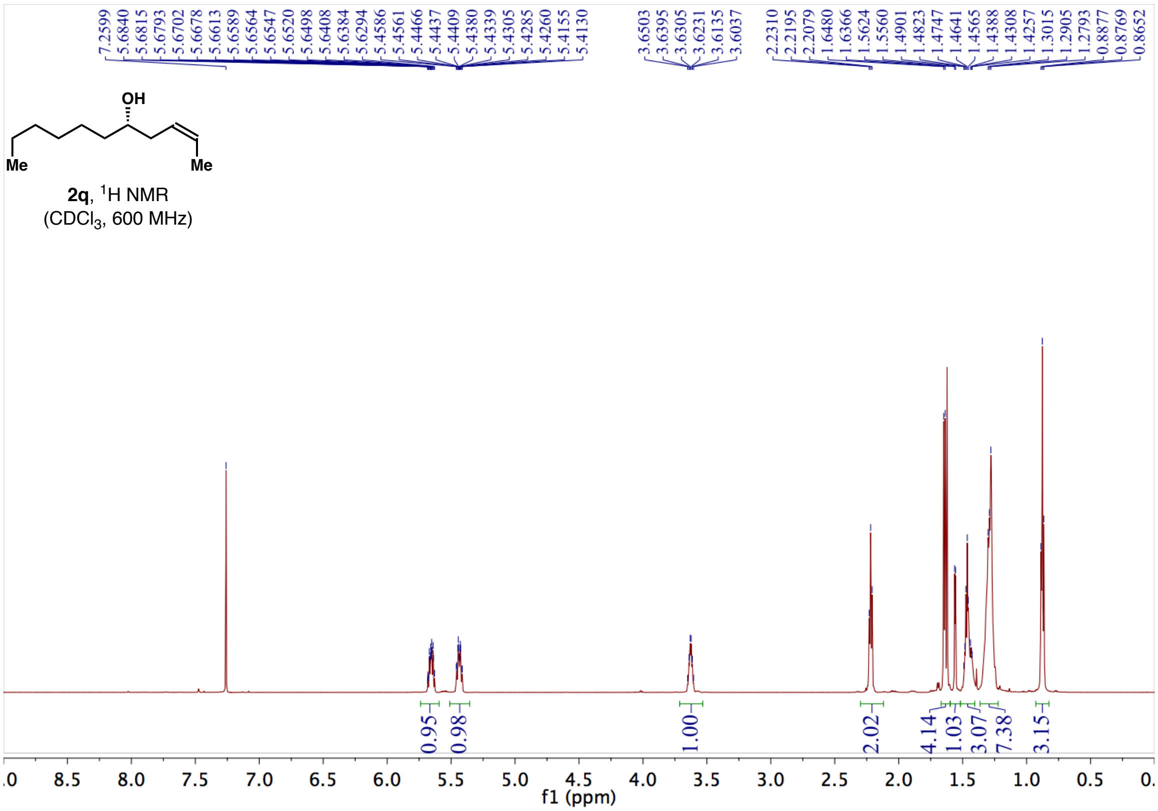
2q, ^{1}H NMR
(CDCl$_3$, 600 MHz)
OH
Me
Me
f1 (ppm)

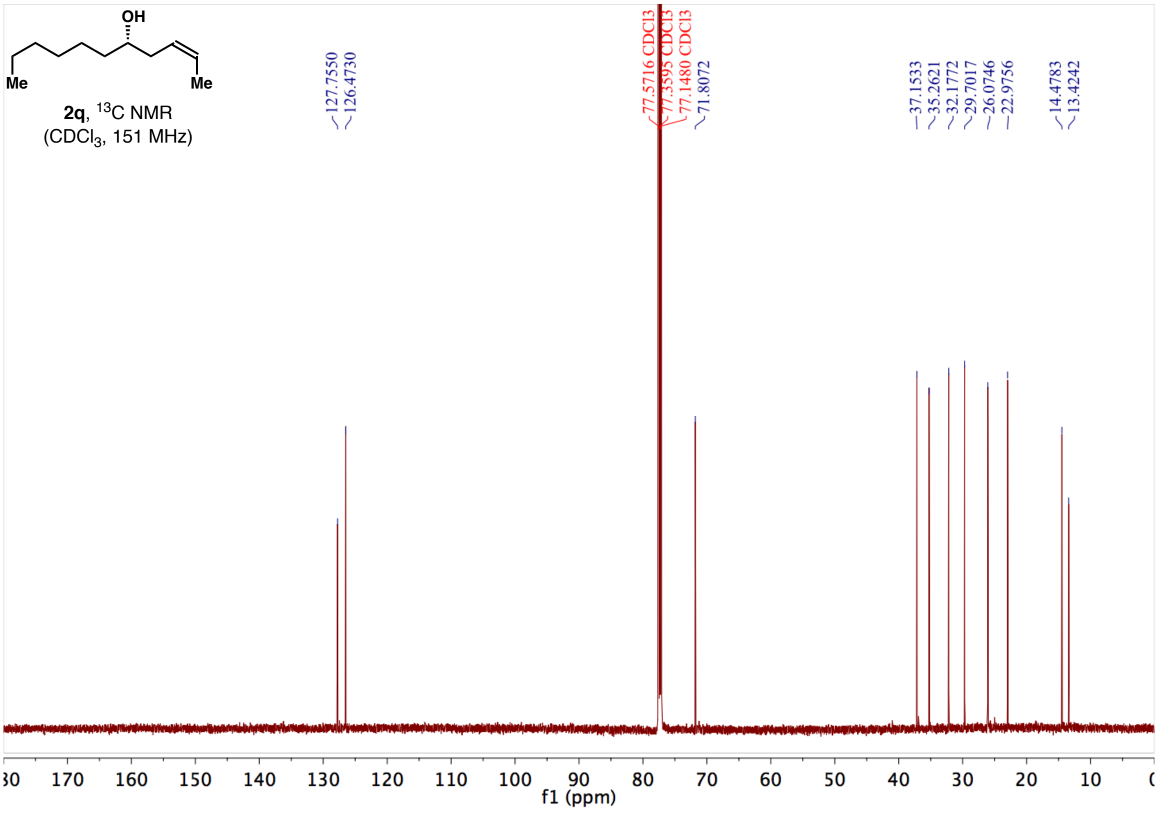
2q, ^{13}C NMR
(CDCl$_3$, 151 MHz)
OH
Me
Me
f1 (ppm)

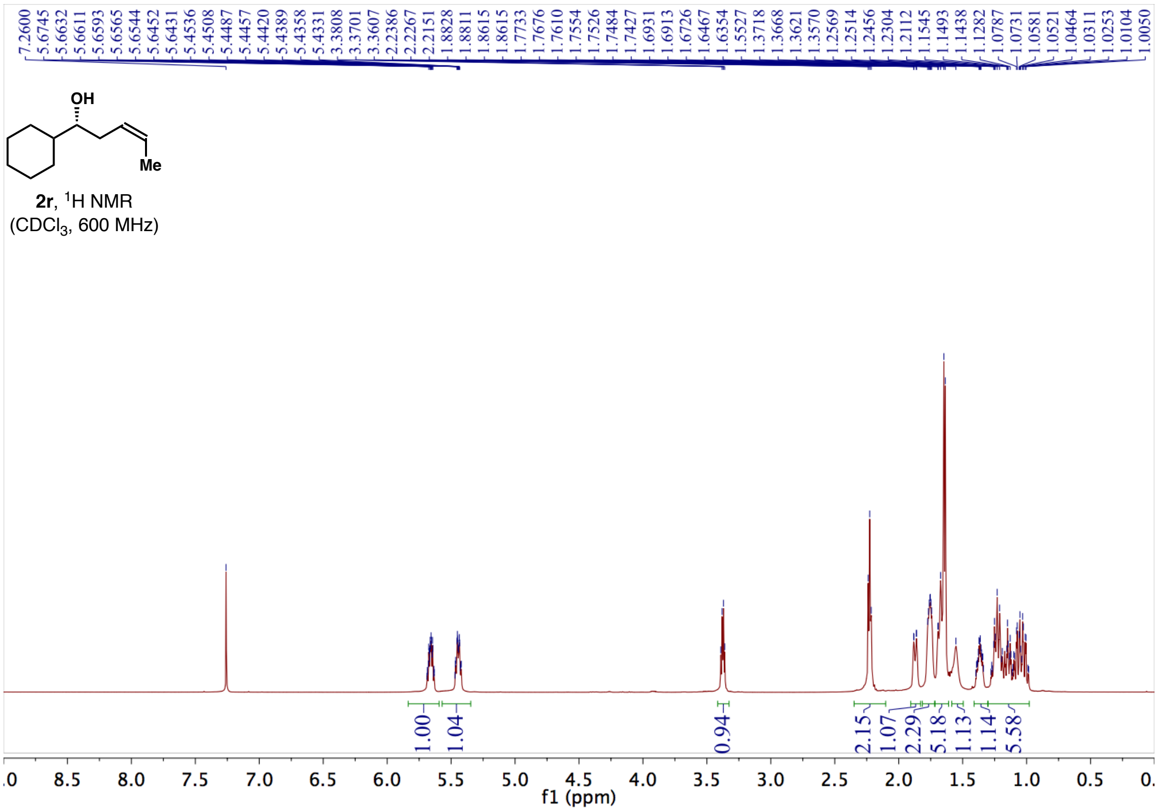

2r, ^{1}H NMR
($CDCl_3$, 600 MHz)

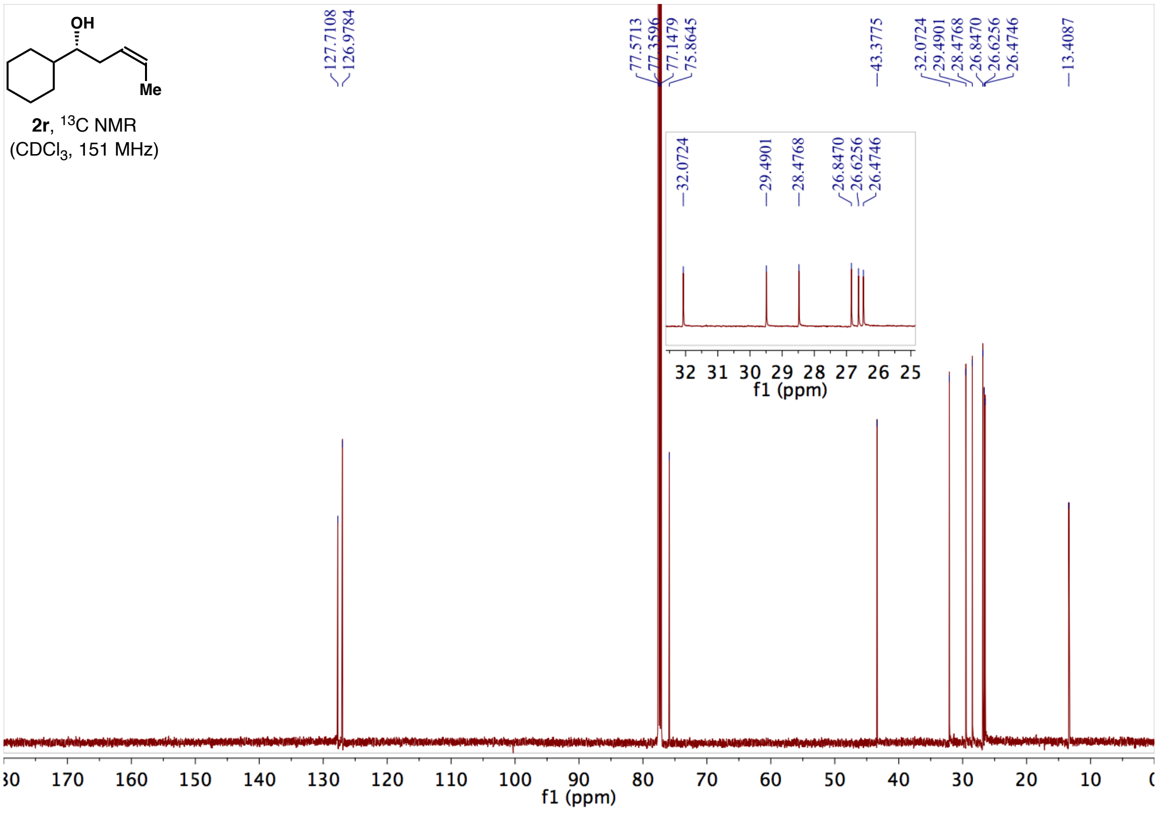

2r, ^{13}C NMR
($CDCl_3$, 151 MHz)

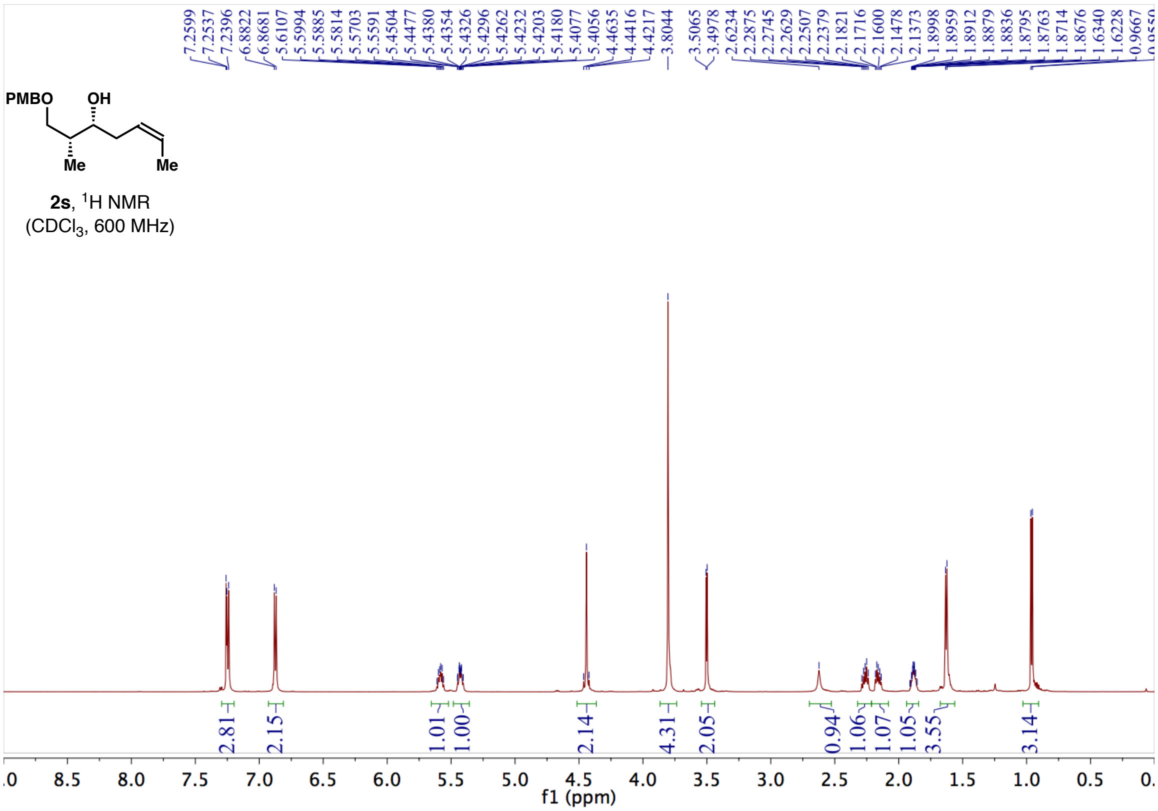
PMBO
OH
Me
Me
2s, ¹H NMR
(CDCl₃, 600 MHz)
f1 (ppm)

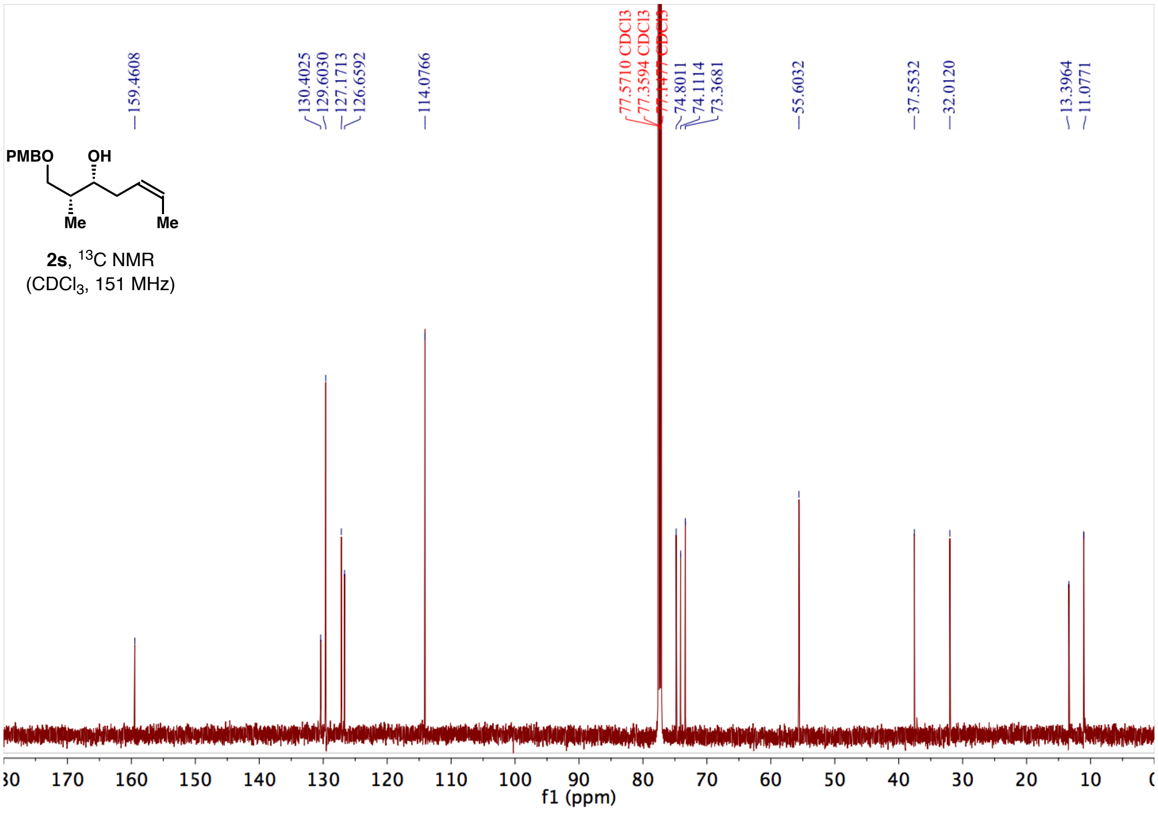
PMBO
OH
Me
Me
2s, ¹³C NMR
(CDCl₃, 151 MHz)
f1 (ppm)

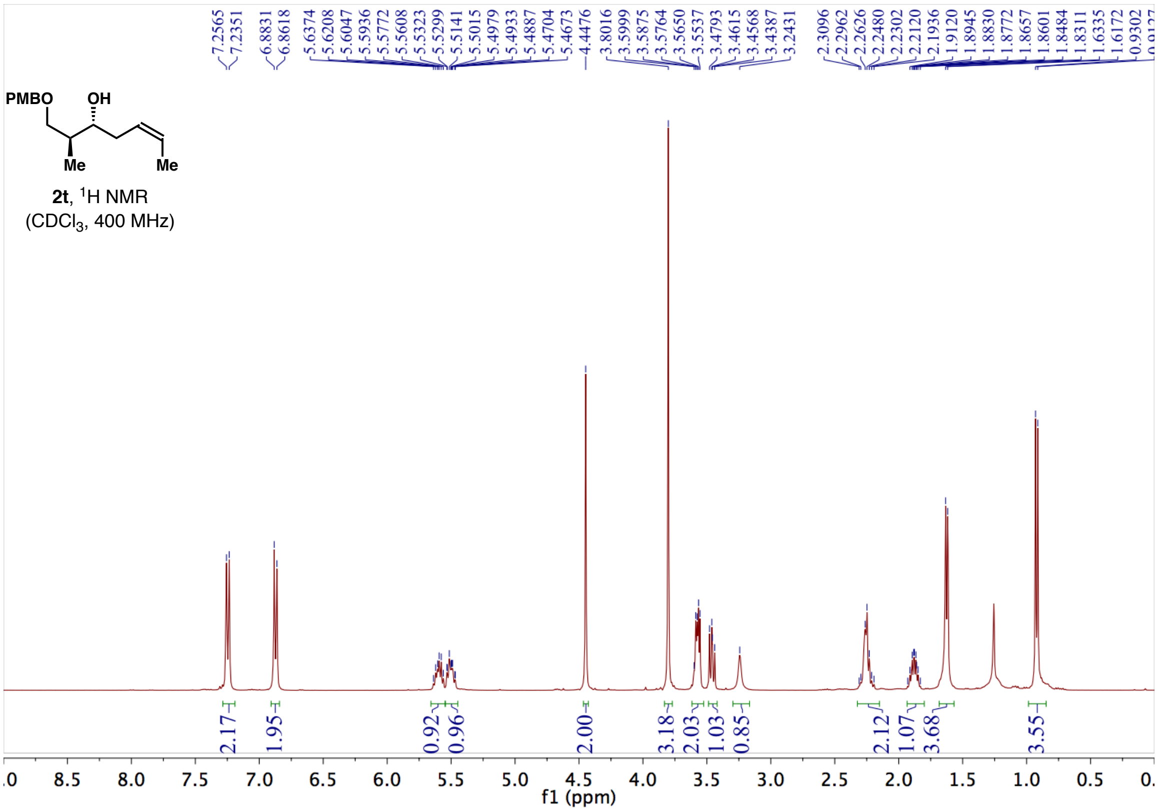

PMBO
OH
Me
Me
2t, ¹H NMR
(CDCl₃, 400 MHz)
7.2565
7.2351
6.8831
6.8618
5.6374
5.6208
5.6047
5.5936
5.5772
5.5608
5.5323
5.5299
5.5141
5.5015
5.4979
5.4933
5.4887
5.4704
5.4673
4.4476
3.8016
3.5999
3.5875
3.5764
3.5650
3.5537
3.4793
3.4615
3.4568
3.4387
3.2431
2.3096
2.2962
2.2626
2.2480
2.2302
2.2120
2.1936
1.9120
1.8945
1.8830
1.8772
1.8657
1.8601
1.8484
1.8311
1.6335
1.6172
0.9302
2.17
1.95
0.92
0.96
2.00
3.18
2.03
1.03
0.85
2.12
1.07
3.68
3.55
f1 (ppm)

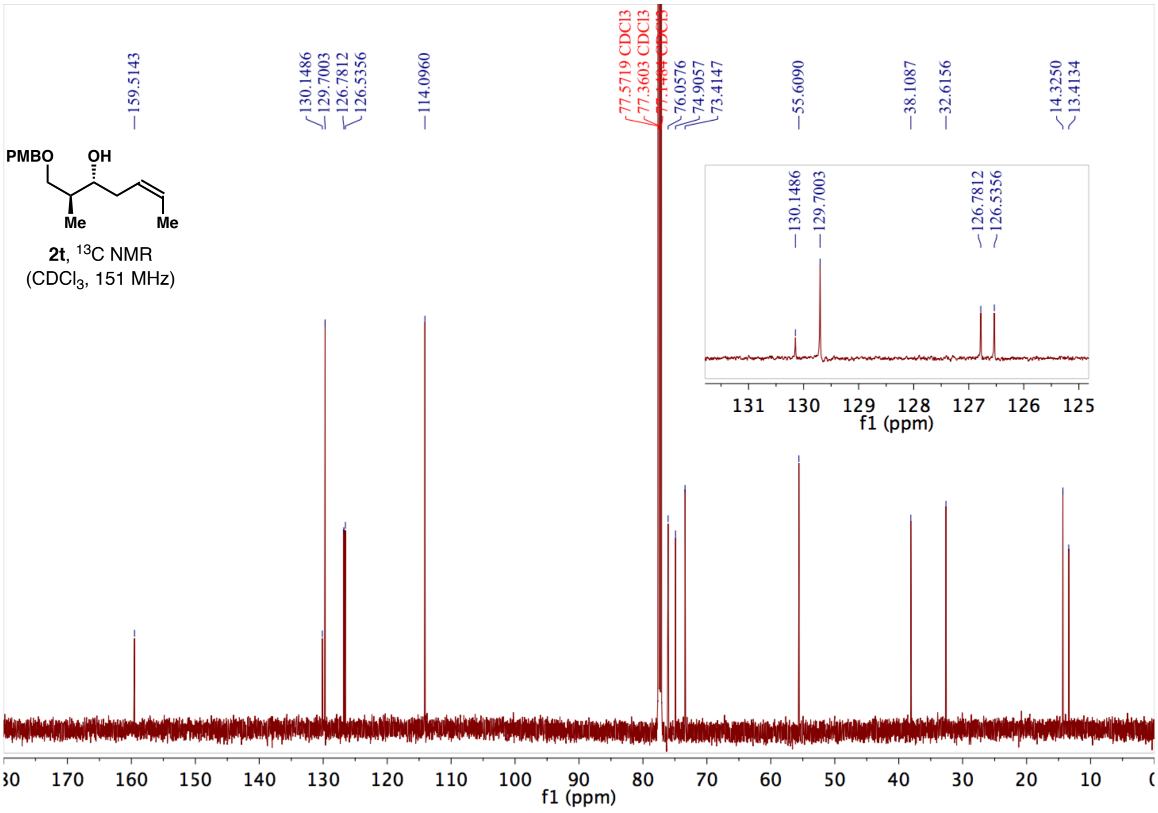

PMBO
OH
Me
Me
2t, ¹³C NMR
(CDCl₃, 151 MHz)
159.5143
130.1486
129.7003
126.7812
126.5356
114.0960
77.5719 CDCl3
77.3603 CDCl3
76.0576
74.9057
73.4147
55.6090
38.1087
32.6156
14.3250
13.4134
f1 (ppm)

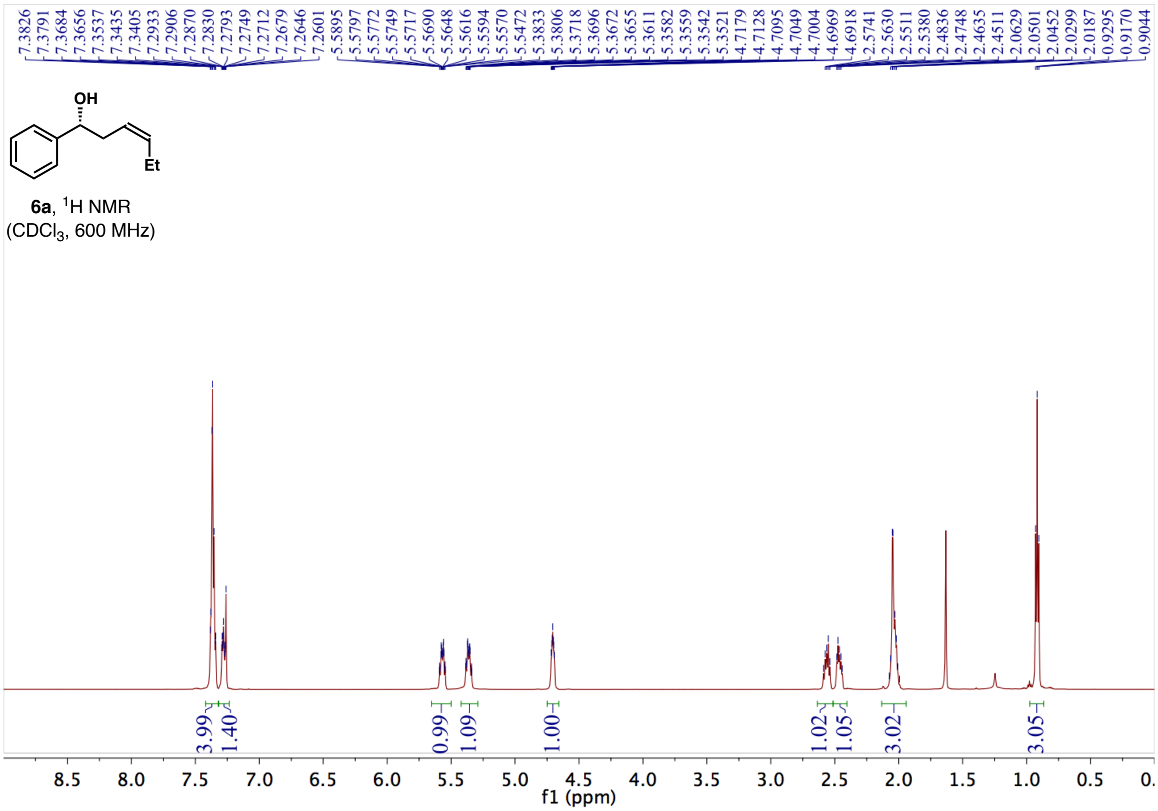
7.3826
7.3791
7.3684
7.3656
7.3537
7.3435
7.3405
7.2933
7.2906
7.2870
7.2830
7.2793
7.2749
7.2712
7.2679
7.2646
7.2601
5.5895
5.5797
5.5772
5.5749
5.5717
5.5690
5.5648
5.5616
5.5594
5.5570
5.5472
5.3833
5.3806
5.3718
5.3696
5.3672
5.3655
5.3611
5.3582
5.3559
5.3542
5.3521
4.7179
4.7128
4.7095
4.7049
4.7004
4.6969
4.6918
2.5741
2.5630
2.5511
2.5380
2.4836
2.4748
2.4635
2.4511
2.0629
2.0501
2.0452
2.0299
2.0187
0.9295
0.9170
0.9044
OH
Et
6a, 1H NMR
(CDCl3, 600 MHz)
3.99
1.40
0.99
1.09
1.00
1.02
1.05
3.02
3.05
8.5 8.0 7.5 7.0 6.5 6.0 5.5 5.0 4.5 4.0 3.5 3.0 2.5 2.0 1.5 1.0 0.5
f1 (ppm)

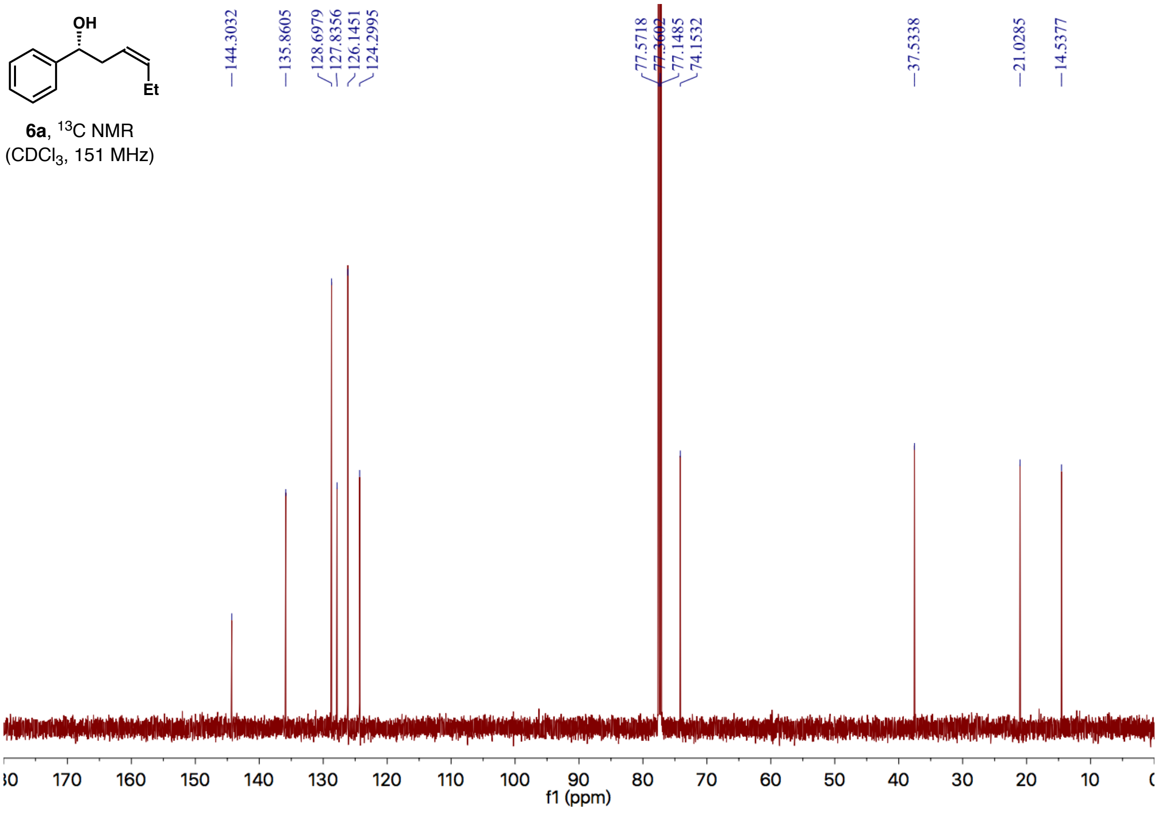
OH
Et
6a, 13C NMR
(CDCl3, 151 MHz)
144.3032
135.8605
128.6979
127.8356
126.1451
124.2995
77.5718
77.1485
74.1532
37.5338
21.0285
14.5377
170 160 150 140 130 120 110 100 90 80 70 60 50 40 30 20 10
f1 (ppm)

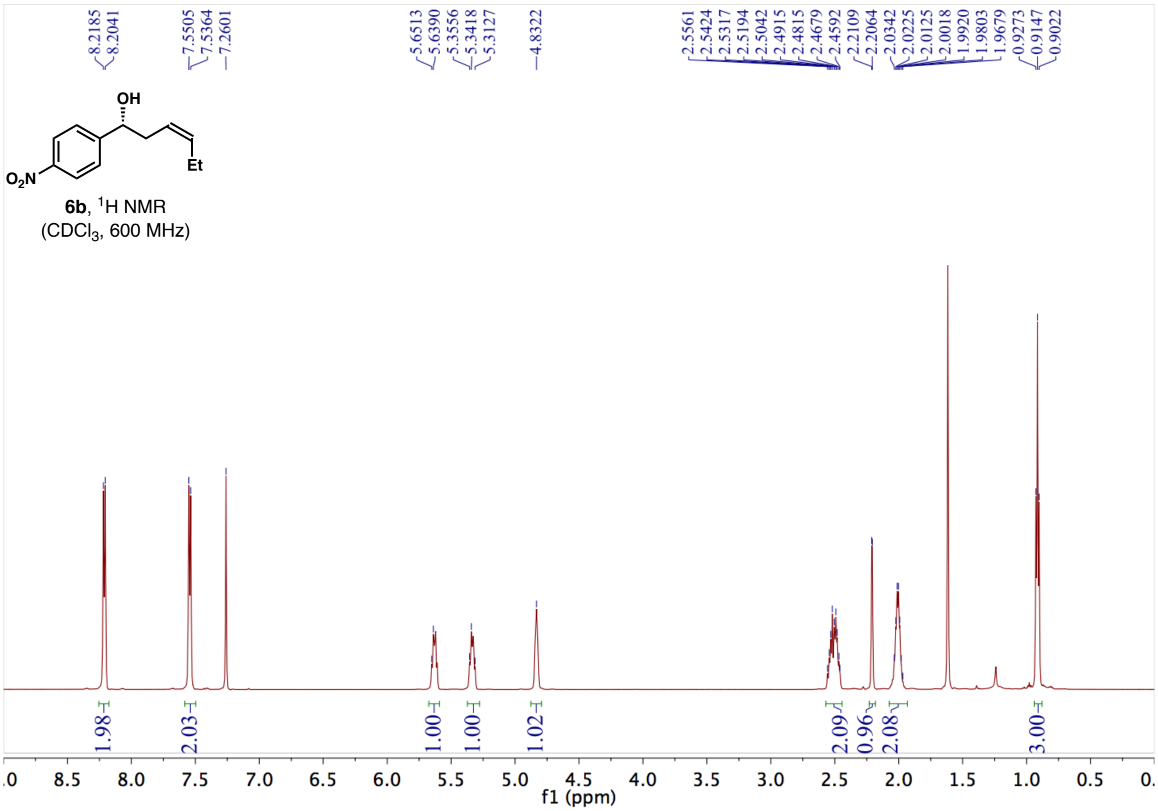
8.2185
8.2041
7.5505
7.5364
7.2601
5.6513
5.6390
5.3556
5.3418
5.3127
4.8322
2.5561
2.5424
2.5317
2.5194
2.5042
2.4915
2.4815
2.4679
2.4592
2.2109
2.2064
2.0342
2.0225
2.0125
2.0018
1.9920
1.9803
1.9679
0.9273
0.9147
0.9022
OH
O_2N
Et
6b, ^{1}H NMR
($CDCl_3$, 600 MHz)
1.98
2.03
1.00
1.00
1.02
2.09
0.96
2.08
3.00
f1 (ppm)

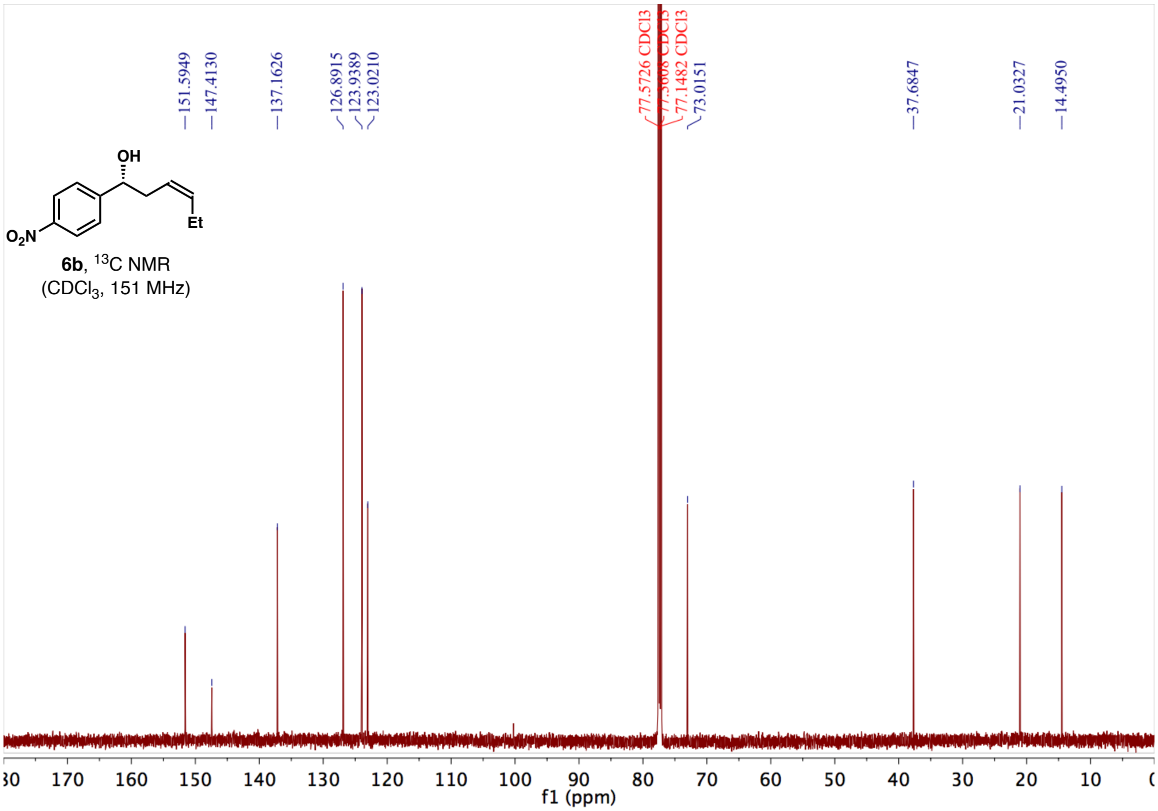
151.5949
147.4130
137.1626
126.8915
123.9389
123.0210
77.5726 CDCl3
77.1482 CDCl3
73.0151
37.6847
21.0327
14.4950
OH
O_2N
Et
6b, ^{13}C NMR
($CDCl_3$, 151 MHz)
f1 (ppm)

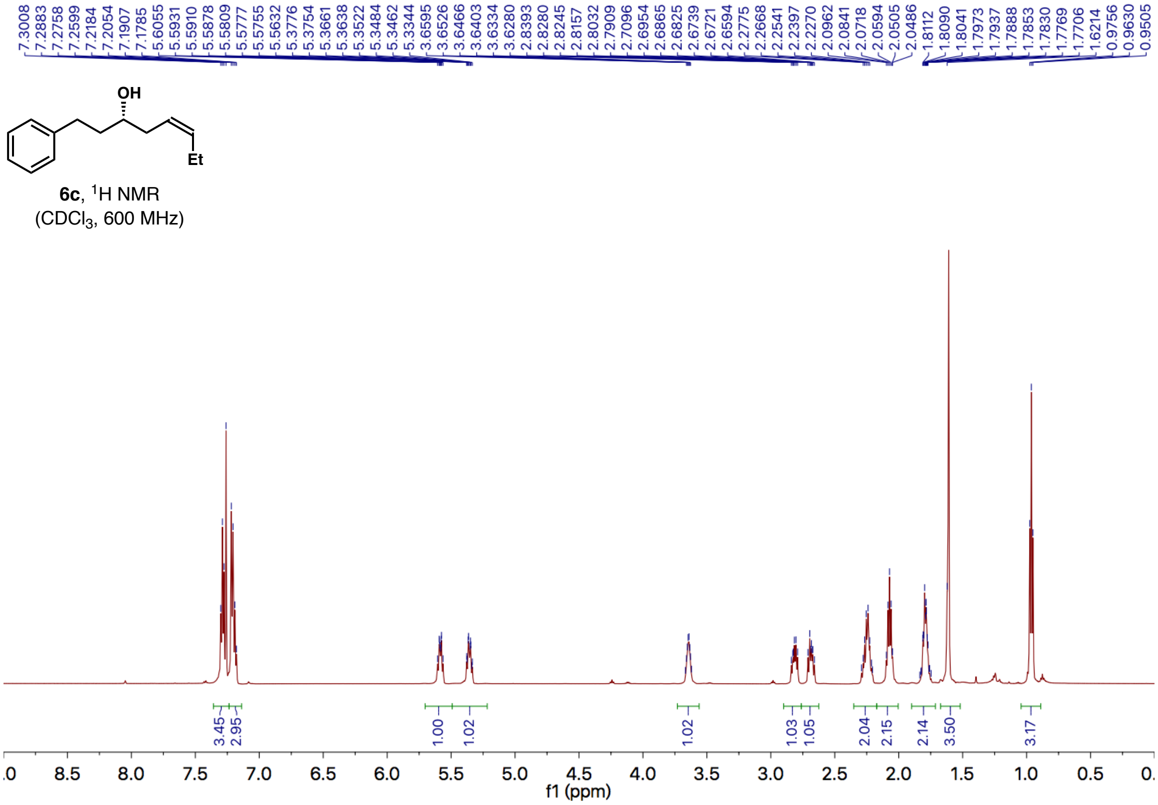
7.3008 7.2883 7.2758 7.2599 7.2184 7.2054 7.1907 7.1785 5.6055 5.5931 5.5910 5.5878 5.5809 5.5777 5.5755 5.5632 5.3776 5.3754 5.3661 5.3638 5.3522 5.3484 5.3462 5.3344 3.6595 3.6526 3.6466 3.6403 3.6334 3.6280 2.8393 2.8280 2.8245 2.8157 2.8032 2.7909 2.7096 2.6954 2.6865 2.6825 2.6739 2.6721 2.6594 2.2775 2.2668 2.2541 2.2397 2.2270 2.0962 2.0841 2.0718 2.0594 2.0505 2.0486 1.8112 1.8090 1.8041 1.7973 1.7937 1.7888 1.7853 1.7830 1.7769 1.7706 1.6214 0.9756 0.9630 0.9505
OH
Et
6c, 1H NMR
(CDCl3, 600 MHz)
3.45 2.95 1.00 1.02 1.02 1.03 1.05 2.04 2.15 2.14 3.50 3.17
f1 (ppm)

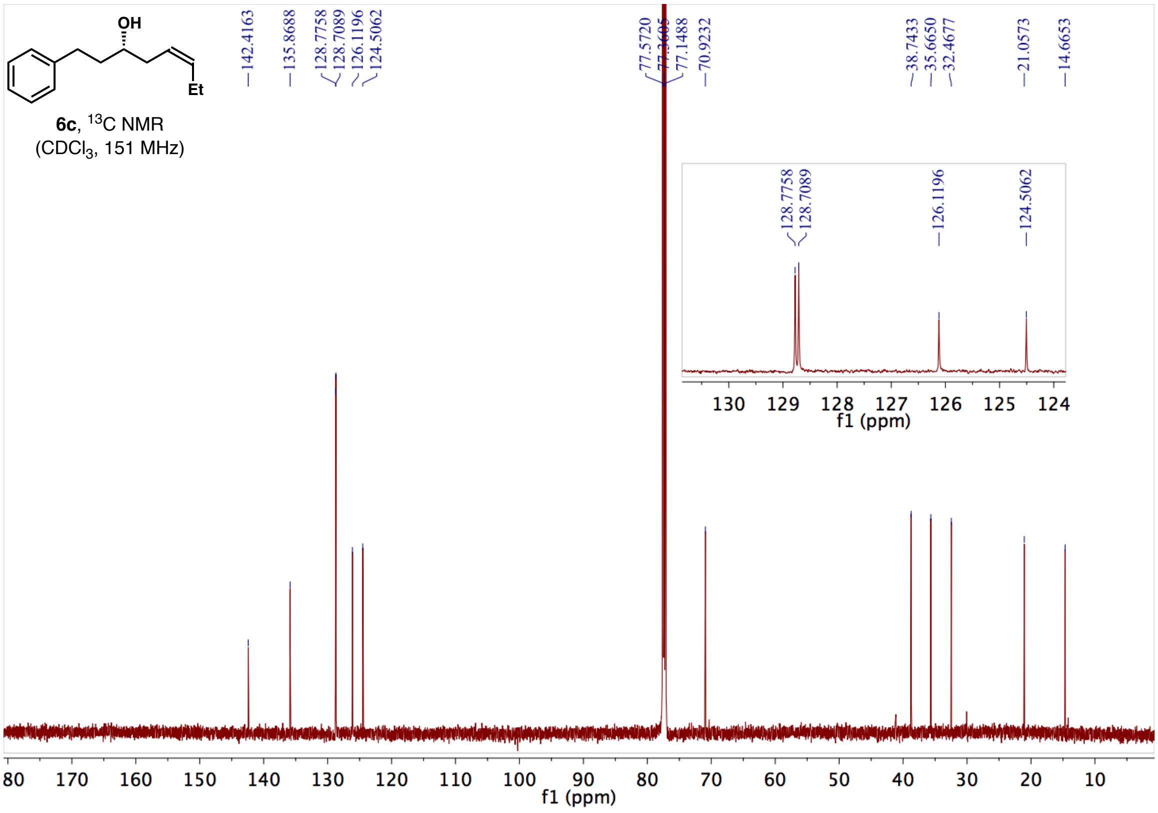
OH
Et
6c, 13C NMR
(CDCl3, 151 MHz)
142.4163 135.8688 128.7758 128.7089 126.1196 124.5062
77.5720 77.3605 77.1488 70.9232
38.7433 35.6650 32.4677 21.0573 14.6653
f1 (ppm)

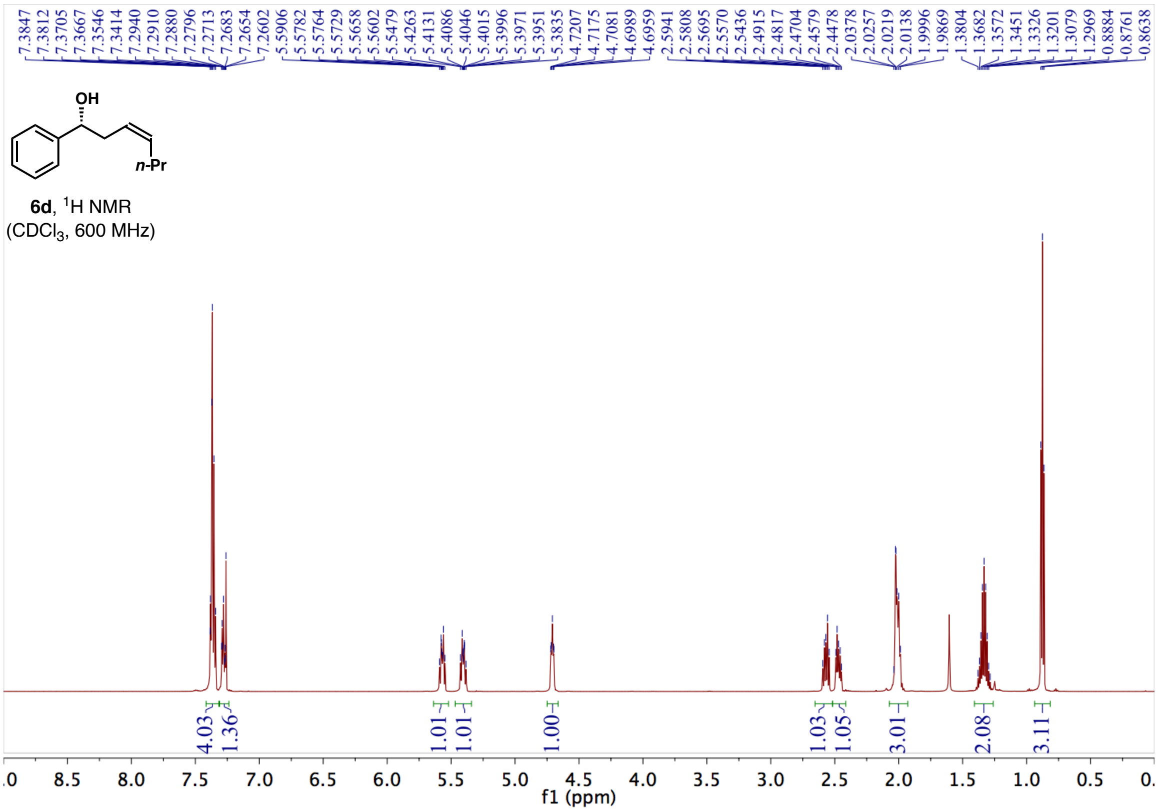
7.3847
7.3812
7.3705
7.3667
7.3546
7.3414
7.2940
7.2910
7.2880
7.2796
7.2713
7.2683
7.2654
7.2602
5.5906
5.5782
5.5764
5.5729
5.5658
5.5602
5.5479
5.4263
5.4131
5.4086
5.4046
5.4015
5.3996
5.3971
5.3951
5.3835
4.7207
4.7175
4.7081
4.6989
4.6959
2.5941
2.5808
2.5695
2.5570
2.5436
2.4915
2.4817
2.4704
2.4579
2.4478
2.0378
2.0257
2.0219
2.0138
1.9996
1.9869
1.3804
1.3682
1.3572
1.3451
1.3326
1.3201
1.3079
1.2969
0.8884
0.8761
0.8638
OH
n-Pr
6d, 1H NMR
(CDCl3, 600 MHz)
4.03
1.36
1.01
1.01
1.00
1.03
1.05
3.01
2.08
3.11
f1 (ppm)

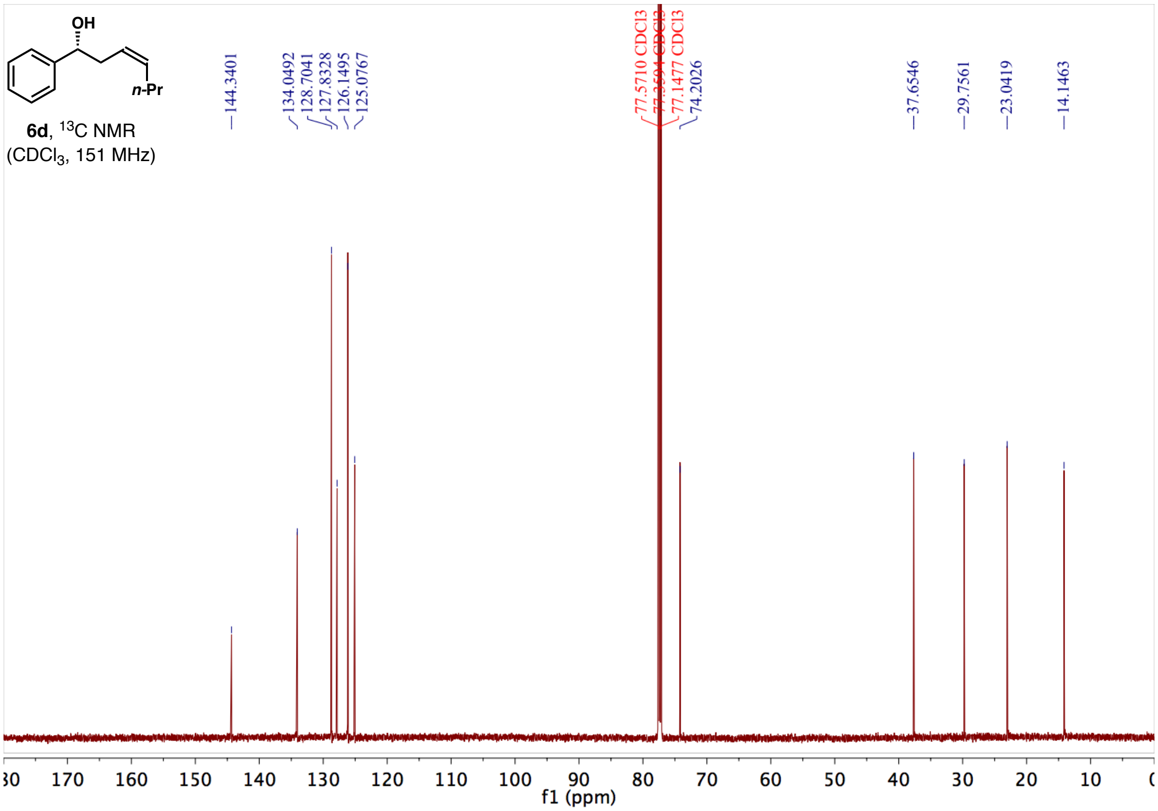
OH
n-Pr
6d, 13C NMR
(CDCl3, 151 MHz)
144.3401
134.0492
128.7041
127.8328
126.1495
125.0767
77.5710 CDCl3
77.3594 CDCl3
77.1477 CDCl3
74.2026
37.6546
29.7561
23.0419
14.1463
f1 (ppm)

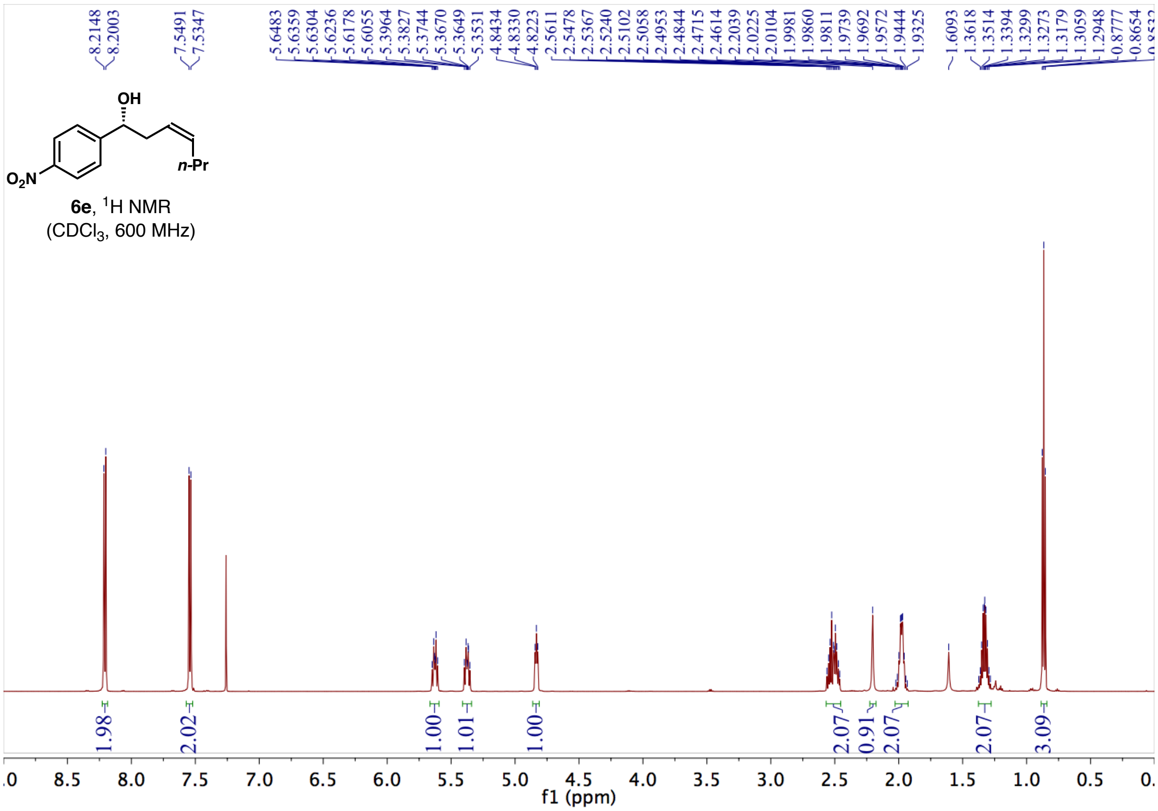
8.2148
8.2003
7.5491
7.5347
5.6483
5.6359
5.6304
5.6236
5.6178
5.6055
5.3964
5.3827
5.3744
5.3670
5.3649
5.3531
4.8434
4.8330
4.8223
2.5611
2.5478
2.5367
2.5240
2.5102
2.5058
2.4953
2.4844
2.4715
2.4614
2.2039
2.0225
2.0104
1.9981
1.9860
1.9811
1.9739
1.9692
1.9572
1.9444
1.9325
1.6093
1.3618
1.3514
1.3394
1.3299
1.3273
1.3179
1.3059
1.2948
0.8777
0.8654
OH
O_2N
n-Pr
6e, ^{1}H NMR
($CDCl_3$, 600 MHz)
1.98
2.02
1.00
1.01
1.00
2.07
0.91
2.07
2.07
3.09
8.5
8.0
7.5
7.0
6.5
6.0
5.5
5.0
4.5
4.0
3.5
3.0
2.5
2.0
1.5
1.0
0.5
f1 (ppm)

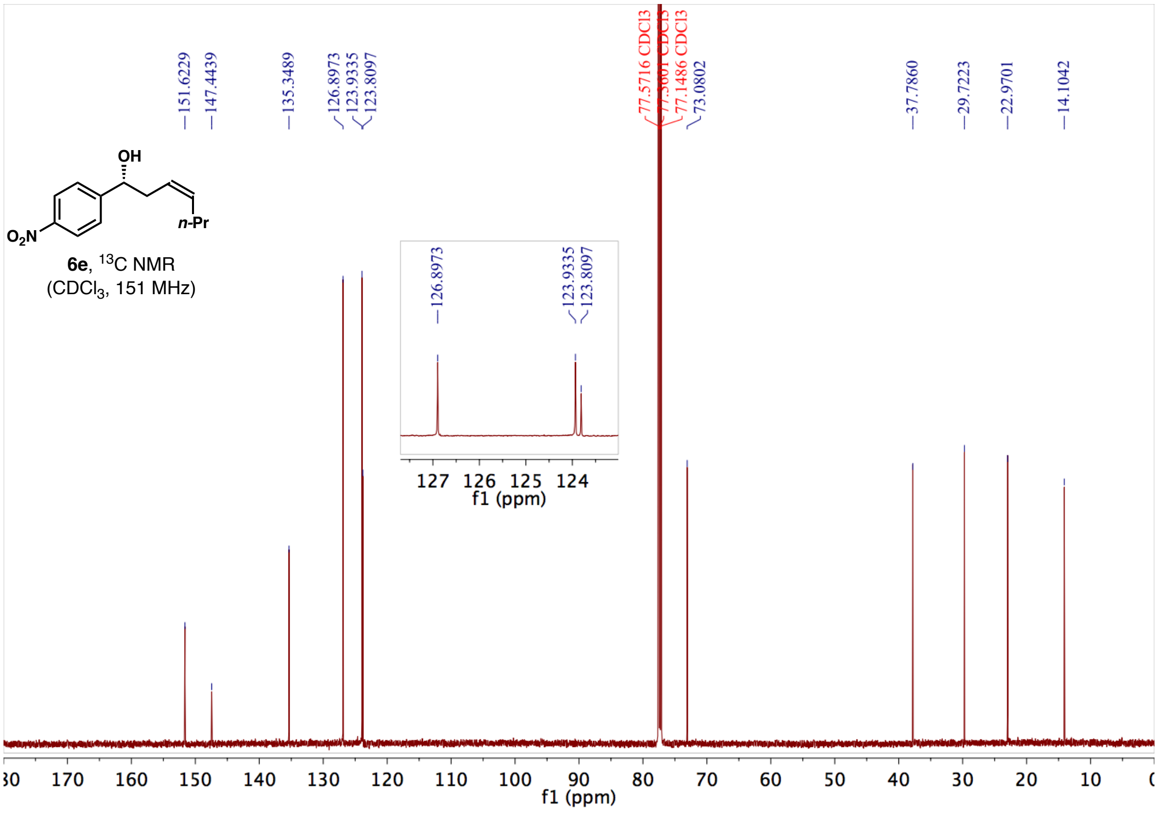
151.6229
147.4439
135.3489
126.8973
123.9335
123.8097
77.5716 CDCl3
77.1486 CDCl3
73.0802
37.7860
29.7223
22.9701
14.1042
OH
O_2N
n-Pr
6e, ^{13}C NMR
($CDCl_3$, 151 MHz)
126.8973
123.9335
123.8097
127
126
125
124
f1 (ppm)
170
160
150
140
130
120
110
100
90
80
70
60
50
40
30
20
10
f1 (ppm)

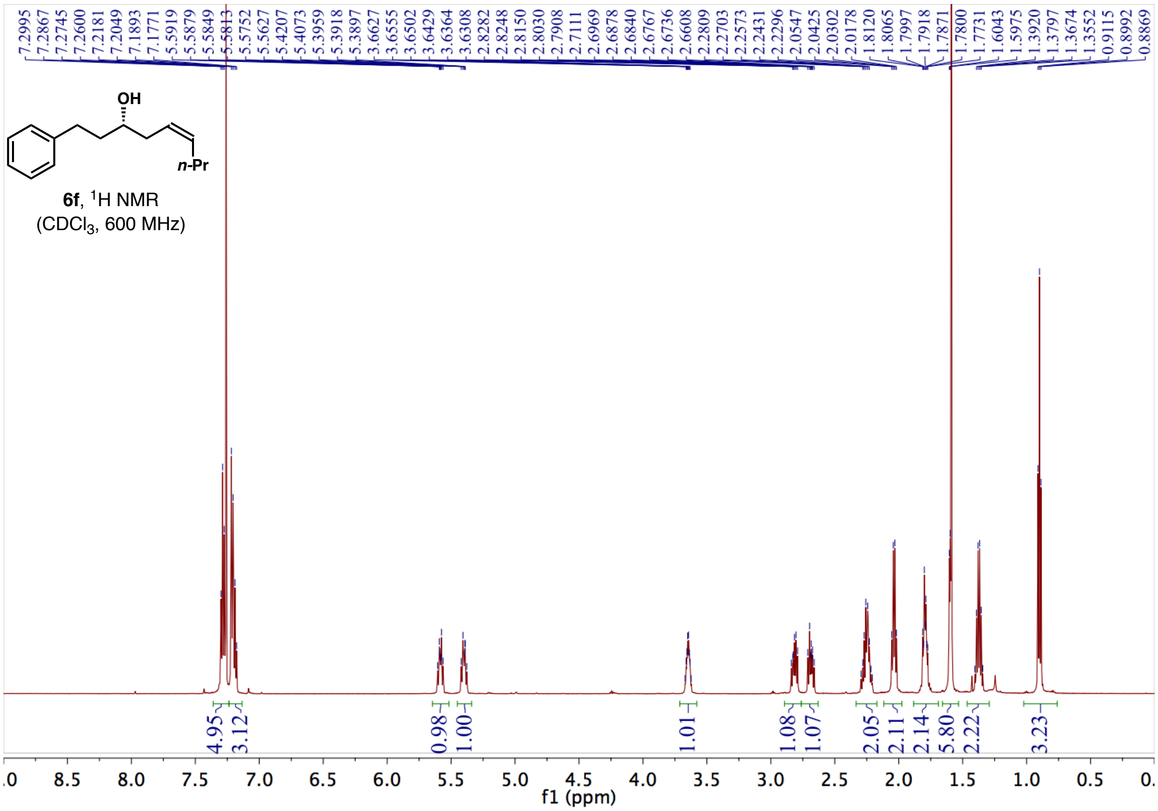
OH
n-Pr
6f, 1H NMR
(CDCl3, 600 MHz)
4.95
3.12
0.98
1.00
1.01
1.08
1.07
2.05
2.11
2.14
5.80
2.22
3.23
f1 (ppm)

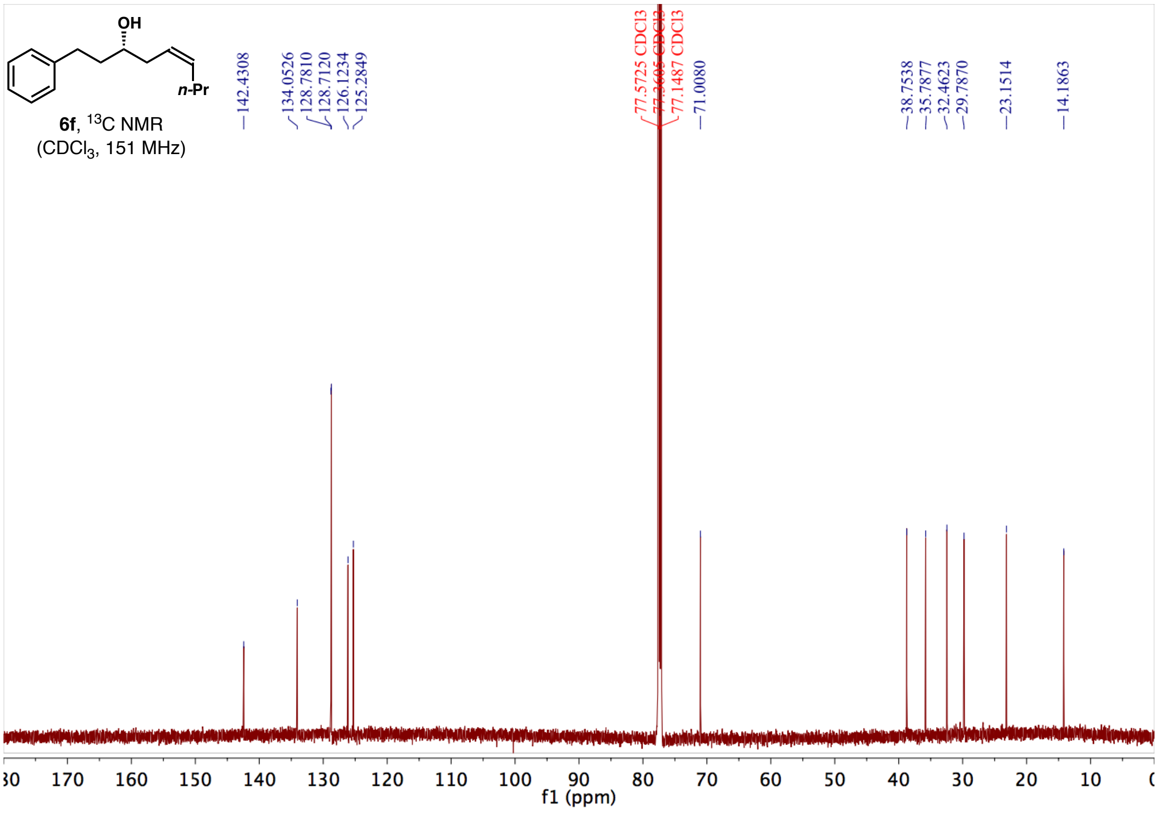
OH
n-Pr
6f, 13C NMR
(CDCl3, 151 MHz)
142.4308
134.0526
128.7810
128.7120
126.1234
125.2849
77.5725 CDCl3
77.1487 CDCl3
71.0080
38.7538
35.7877
32.4623
29.7870
23.1514
14.1863
f1 (ppm)

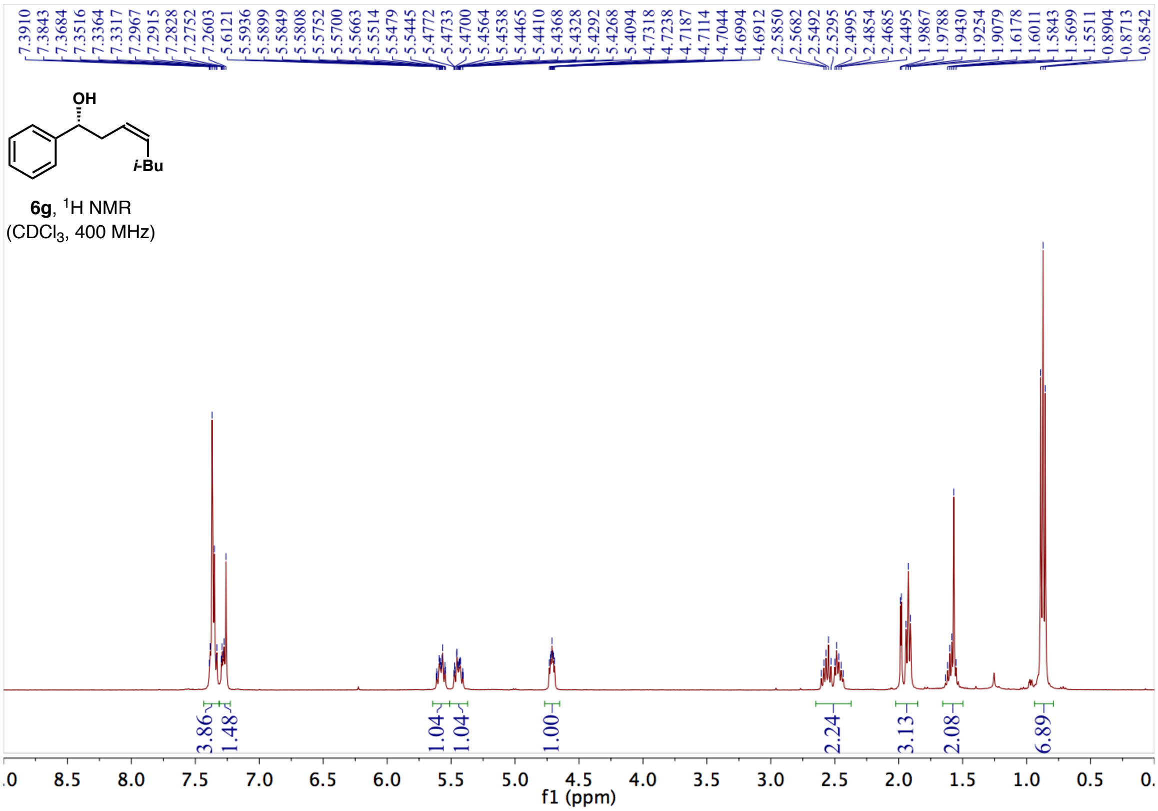
OH
i-Bu
6g, 1H NMR
(CDCl3, 400 MHz)
f1 (ppm)

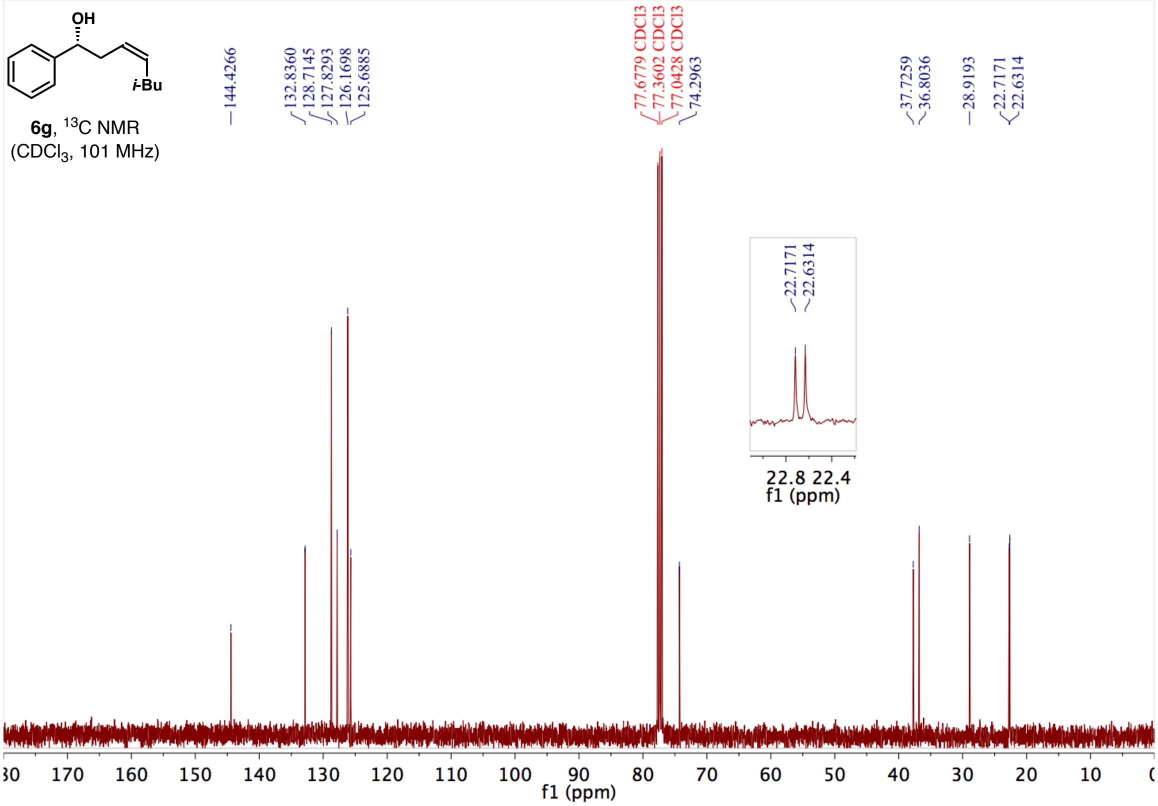
OH
i-Bu
6g, 13C NMR
(CDCl3, 101 MHz)
144.4266
132.8360
128.7145
127.8293
126.1698
125.6885
77.6779 CDCl3
77.3602 CDCl3
77.0428 CDCl3
74.2963
37.7259
36.8036
28.9193
22.7171
22.6314
f1 (ppm)

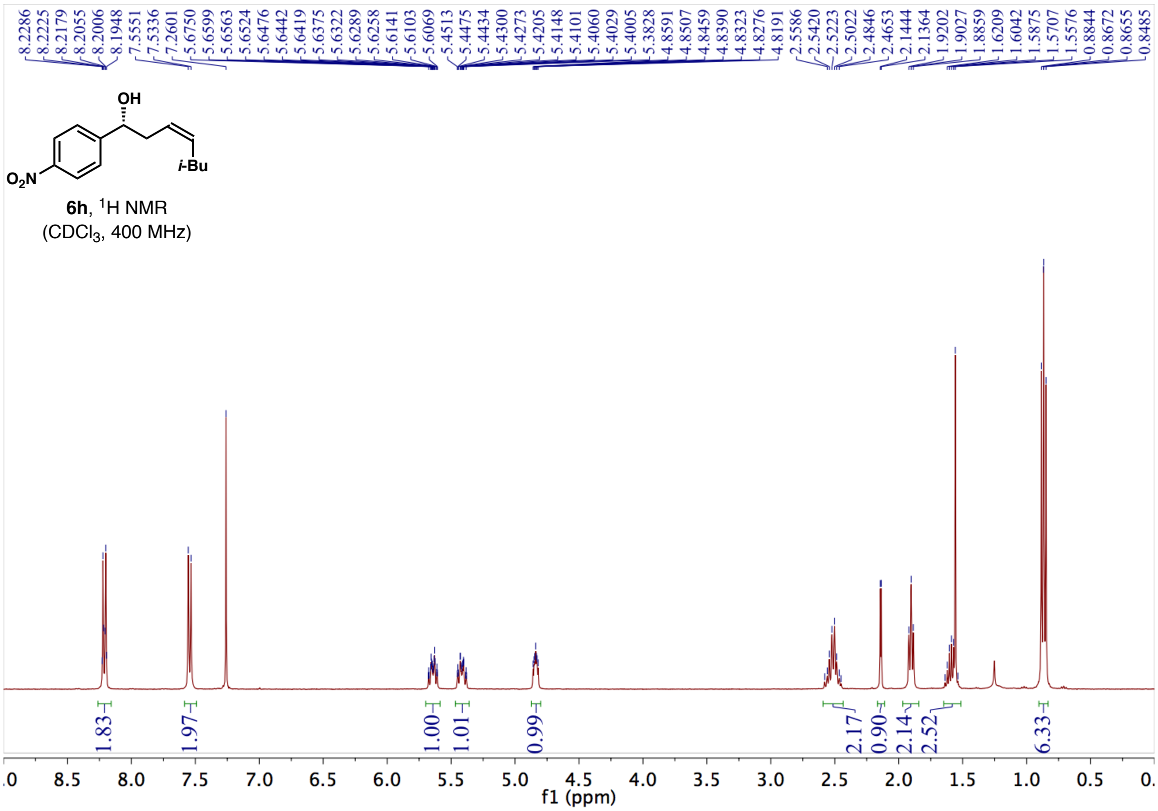
8.2286
8.2225
8.2179
8.2055
8.2006
8.1948
7.5551
7.5336
7.2601
5.6750
5.6599
5.6563
5.6524
5.6476
5.6442
5.6419
5.6375
5.6322
5.6289
5.6258
5.6141
5.6103
5.6069
5.4513
5.4475
5.4434
5.4300
5.4273
5.4205
5.4148
5.4101
5.4060
5.4029
5.4005
5.3828
4.8591
4.8507
4.8459
4.8390
4.8323
4.8276
4.8191
2.5586
2.5420
2.5223
2.5022
2.4846
2.4653
2.1444
2.1364
1.9202
1.9027
1.8859
1.6209
1.6042
1.5875
1.5707
1.5576
0.8844
0.8672
0.8655
0.8485
OH
O2N
i-Bu
6h, 1H NMR
(CDCl3, 400 MHz)
1.83
1.97
1.00
1.01
0.99
2.17
0.90
2.14
2.52
6.33
8.5 8.0 7.5 7.0 6.5 6.0 5.5 5.0 4.5 4.0 3.5 3.0 2.5 2.0 1.5 1.0 0.5
f1 (ppm)

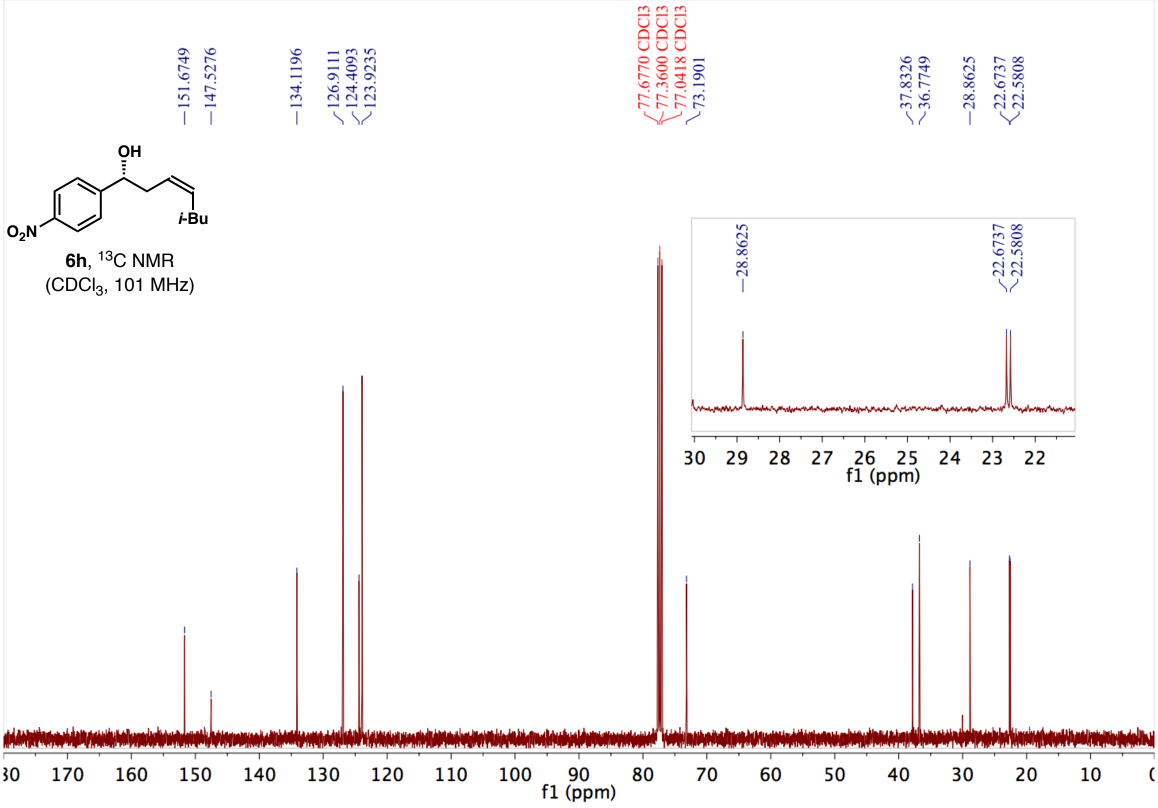
151.6749
147.5276
134.1196
126.9111
124.4093
123.9235
77.6770 CDCl3
77.3600 CDCl3
77.0418 CDCl3
73.1901
37.8326
36.7749
28.8625
22.6737
22.5808
OH
O2N
i-Bu
6h, 13C NMR
(CDCl3, 101 MHz)
28.8625
22.6737
22.5808
30 29 28 27 26 25 24 23 22
f1 (ppm)
170 160 150 140 130 120 110 100 90 80 70 60 50 40 30 20 10
f1 (ppm)

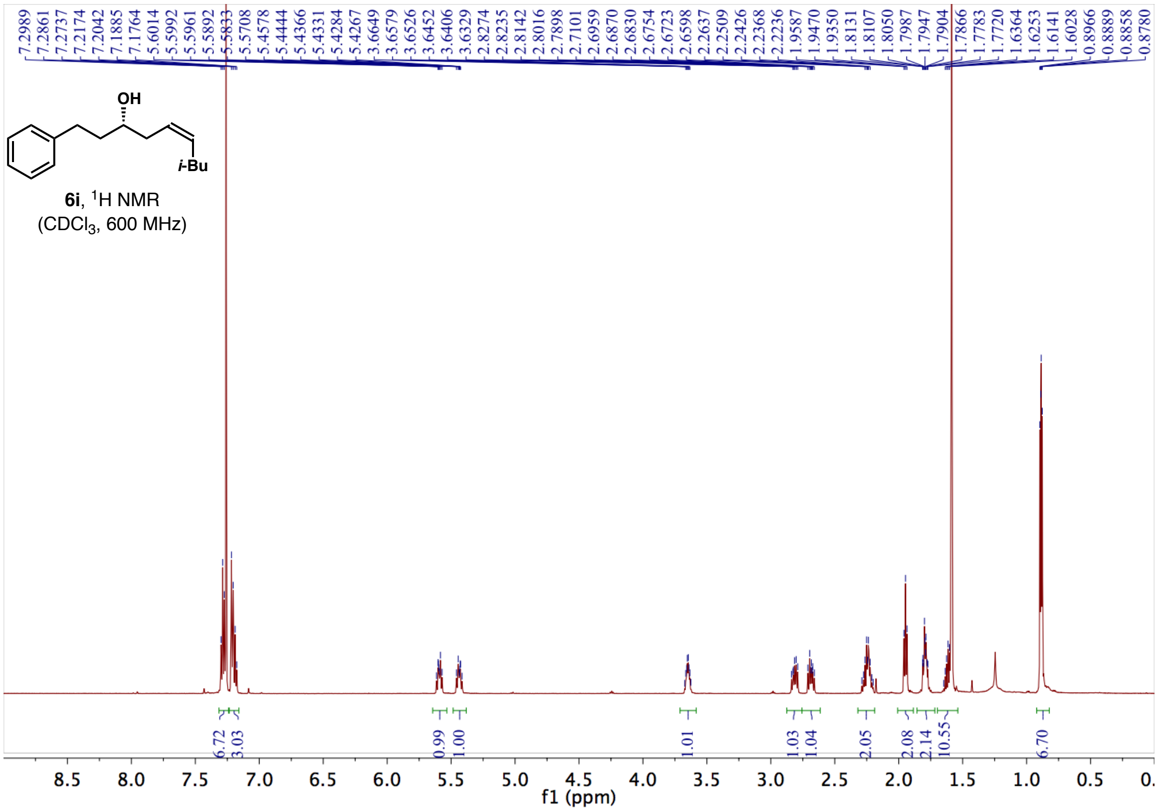
OH
i-Bu
6i, 1H NMR
(CDCl3, 600 MHz)
f1 (ppm)

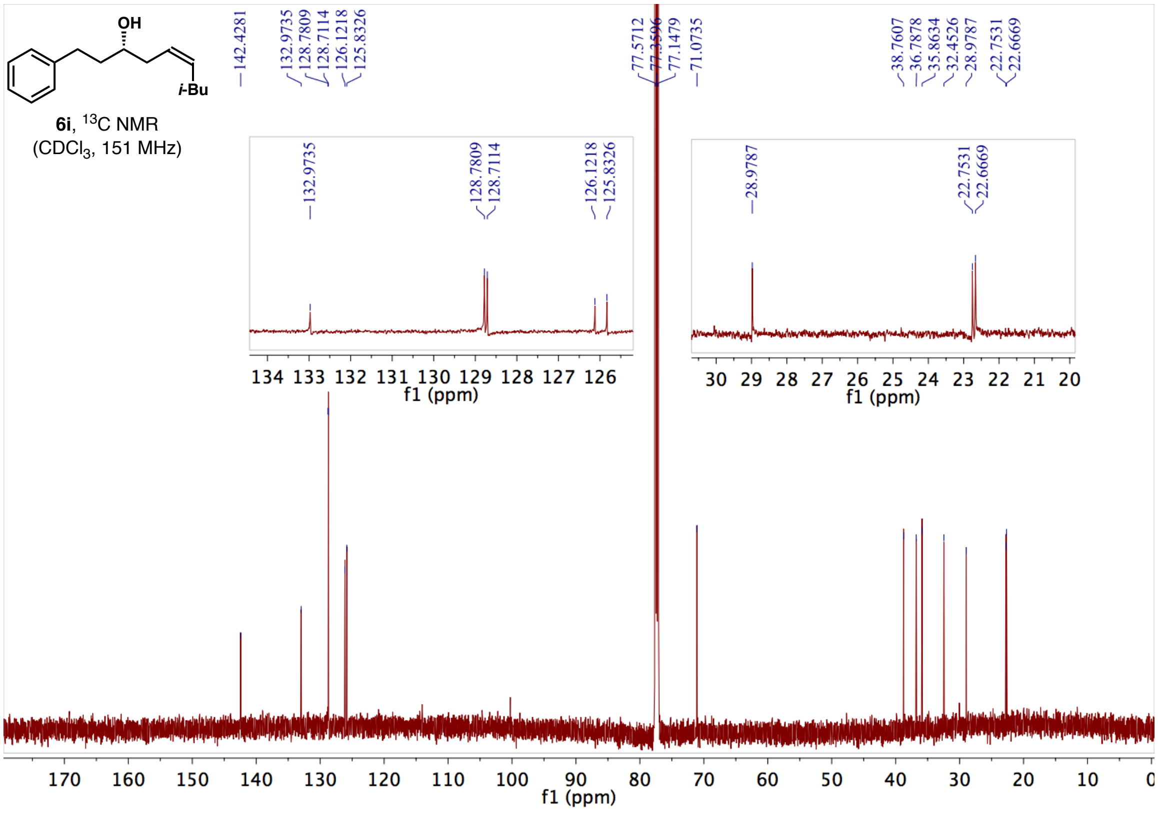
OH
i-Bu
6i, 13C NMR
(CDCl3, 151 MHz)
142.4281
132.9735
128.7809
128.7114
126.1218
125.8326
77.5712
77.3596
77.1479
71.0735
38.7607
36.7878
35.8634
32.4526
28.9787
22.7531
22.6669
f1 (ppm)

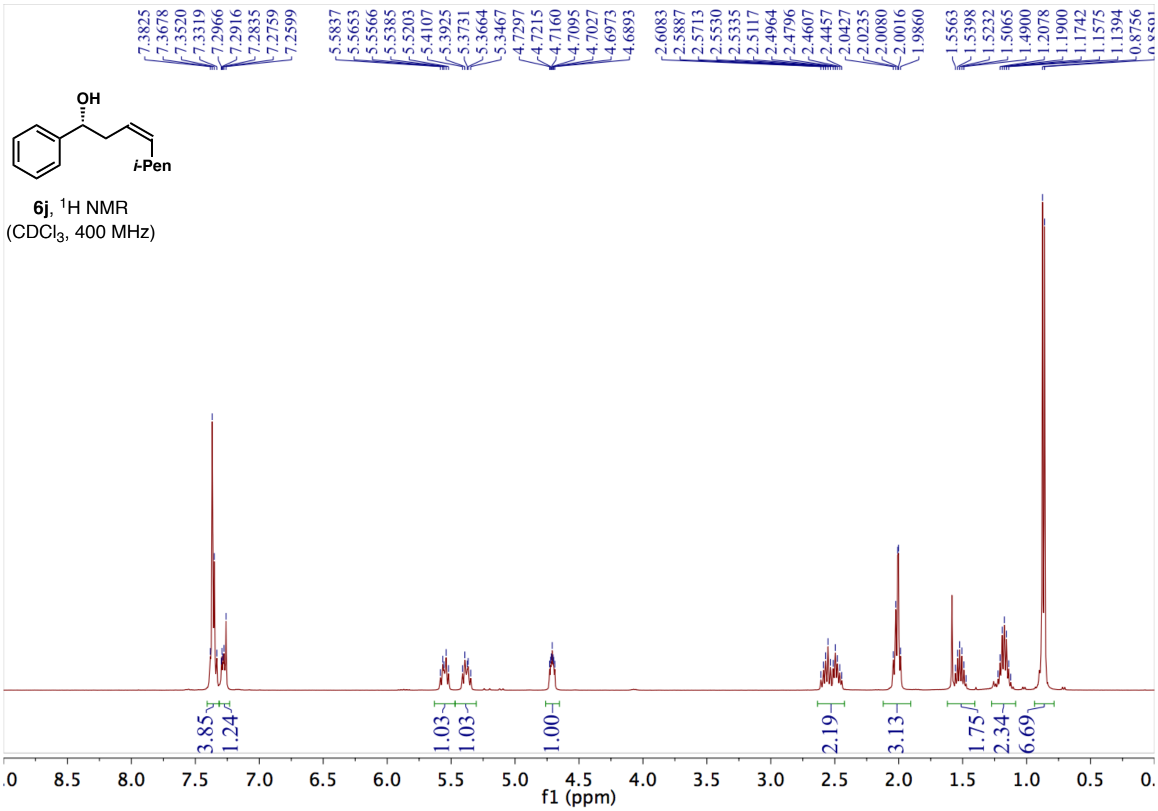

OH
i-Pen
6j, ¹H NMR
(CDCl₃, 400 MHz)
f1 (ppm)

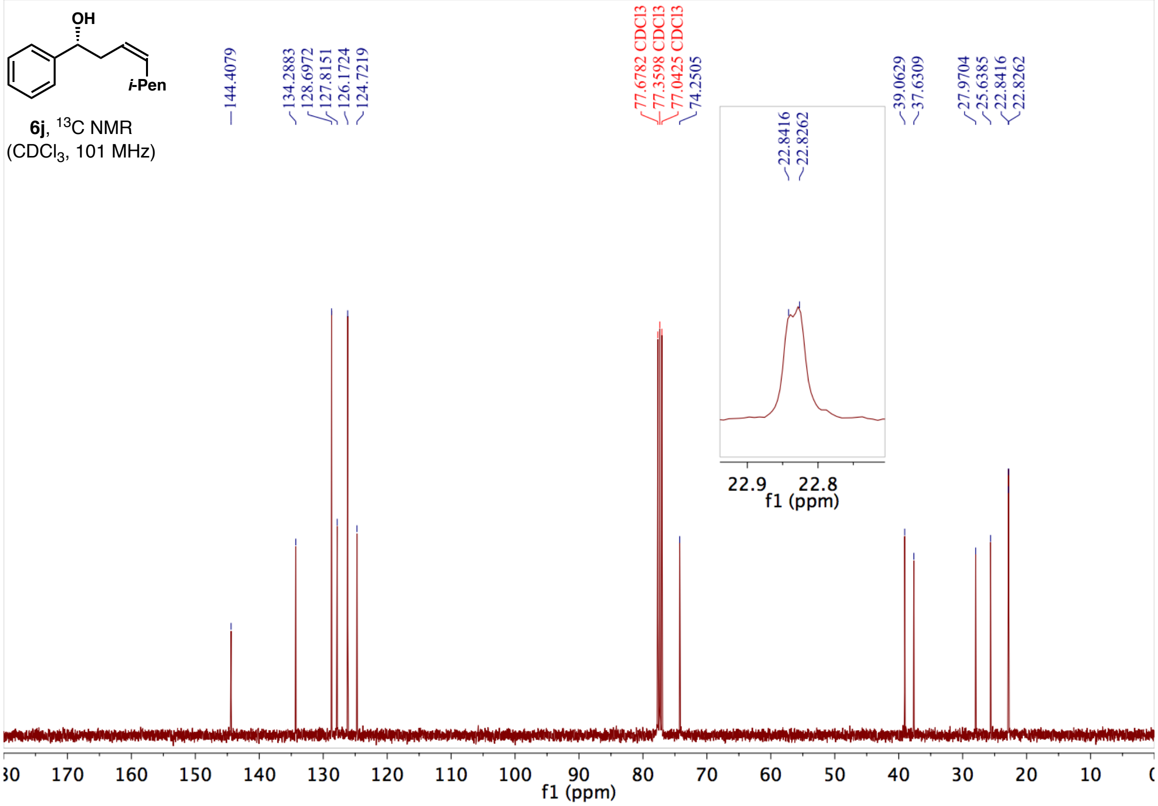

OH
i-Pen
6j, ¹³C NMR
(CDCl₃, 101 MHz)
144.4079
134.2883
128.6972
127.8151
126.1724
124.7219
77.6782 CDCl3
77.3598 CDCl3
77.0425 CDCl3
74.2505
39.0629
37.6309
27.9704
25.6385
22.8416
22.8262
f1 (ppm)

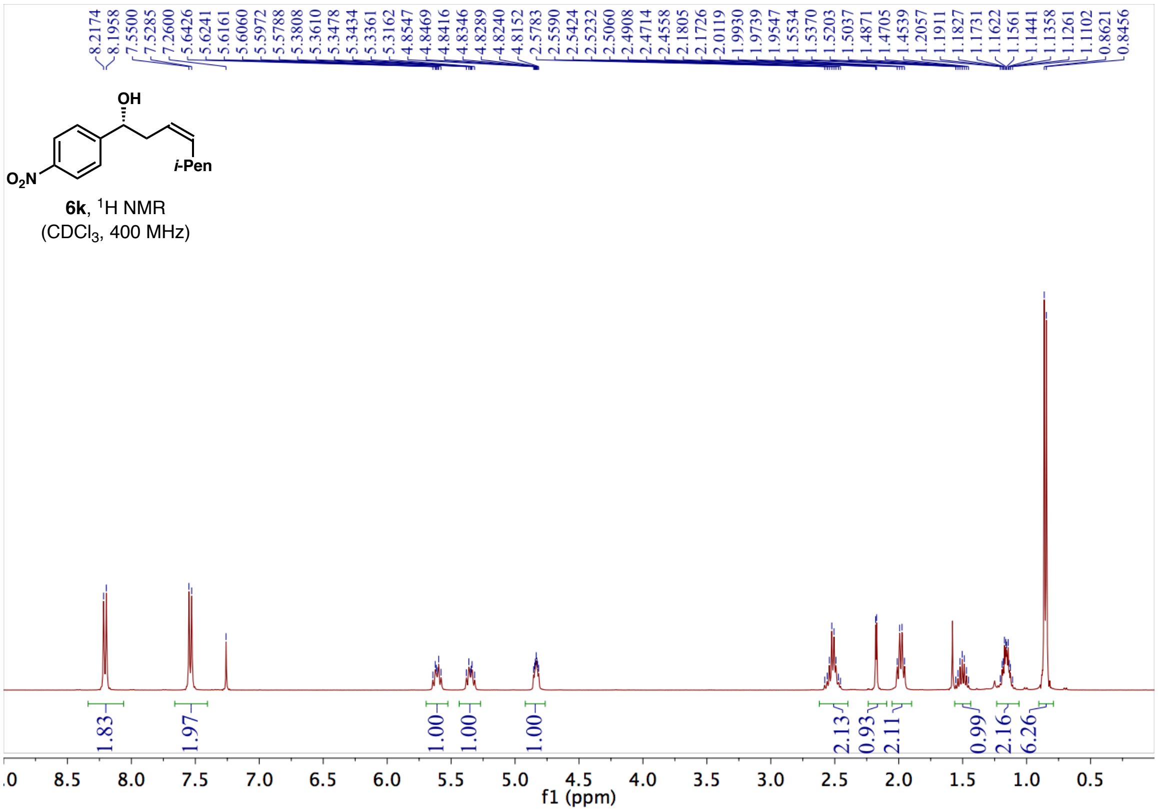
8.2174
8.1958
7.5500
7.5285
7.2600
5.6426
5.6241
5.6161
5.6060
5.5972
5.5788
5.3808
5.3610
5.3478
5.3434
5.3361
5.3162
4.8547
4.8469
4.8416
4.8346
4.8289
4.8240
4.8152
2.5783
2.5590
2.5424
2.5232
2.5060
2.4908
2.4714
2.4558
2.1805
2.1726
2.0119
1.9930
1.9739
1.9547
1.5534
1.5370
1.5203
1.5037
1.4871
1.4705
1.4539
1.2057
1.1911
1.1827
1.1731
1.1622
1.1561
1.1441
1.1358
1.1261
1.1102
0.8621
0.8456
OH
O_2N
i-Pen
6k, ^{1}H NMR
($CDCl_3$, 400 MHz)
1.83
1.97
1.00
1.00
1.00
2.13
0.93
2.11
0.99
2.16
6.26
8.5 8.0 7.5 7.0 6.5 6.0 5.5 5.0 4.5 4.0 3.5 3.0 2.5 2.0 1.5 1.0 0.5
f1 (ppm)

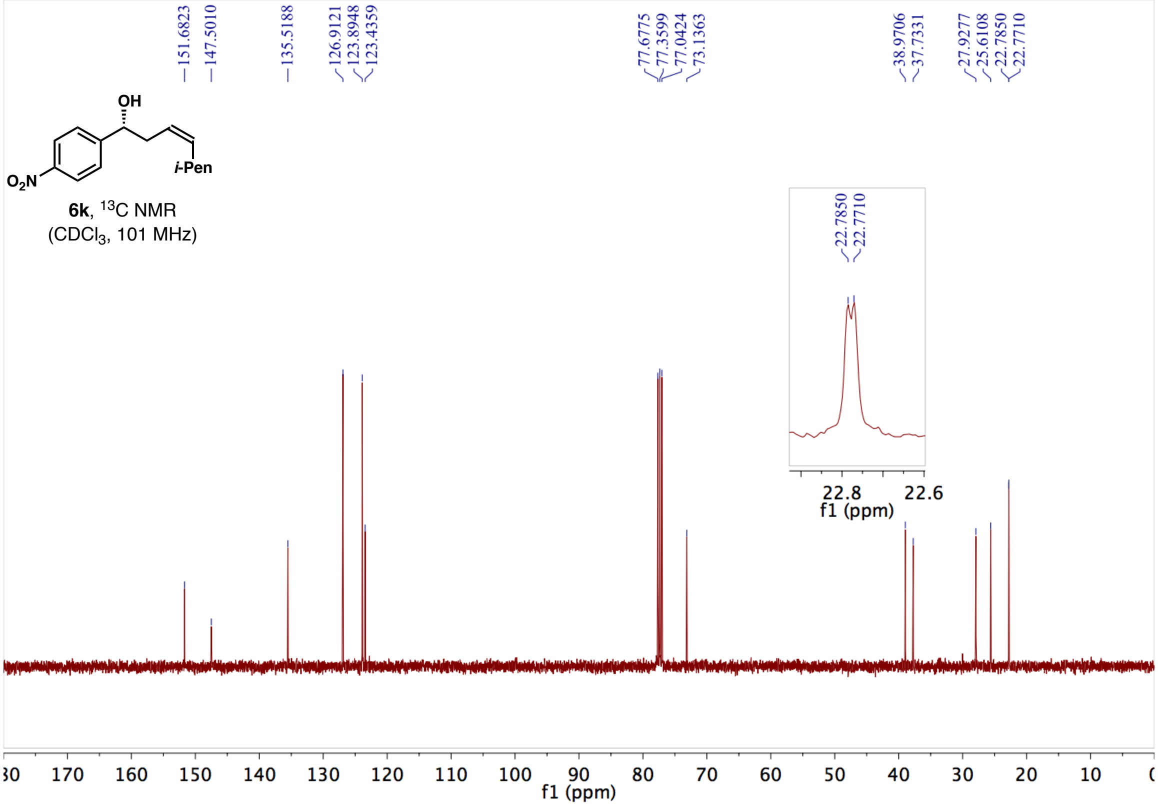
151.6823
147.5010
135.5188
126.9121
123.8948
123.4359
77.6775
77.3599
77.0424
73.1363
38.9706
37.7331
27.9277
25.6108
22.7850
22.7710
OH
O_2N
i-Pen
6k, ^{13}C NMR
($CDCl_3$, 101 MHz)
22.7850
22.7710
22.8 22.6
f1 (ppm)
170 160 150 140 130 120 110 100 90 80 70 60 50 40 30 20 10
f1 (ppm)

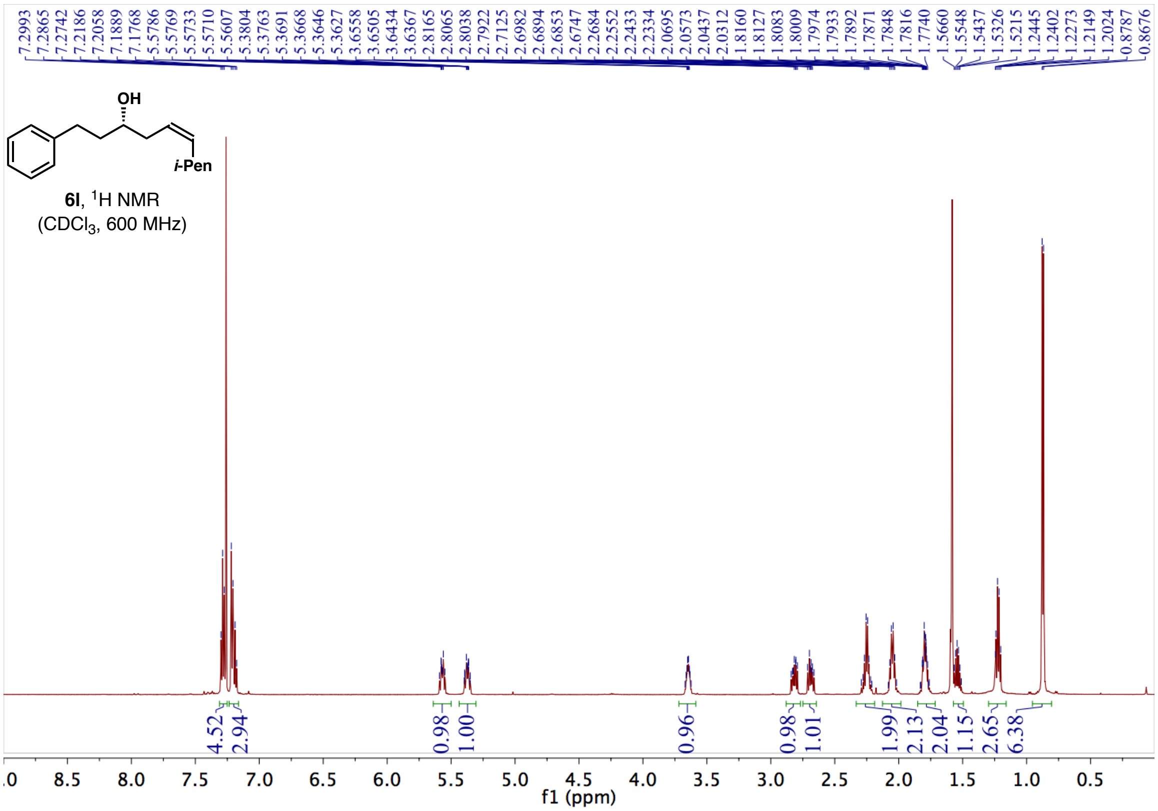

OH
i-Pen
6l, 1H NMR
(CDCl3, 600 MHz)
f1 (ppm)

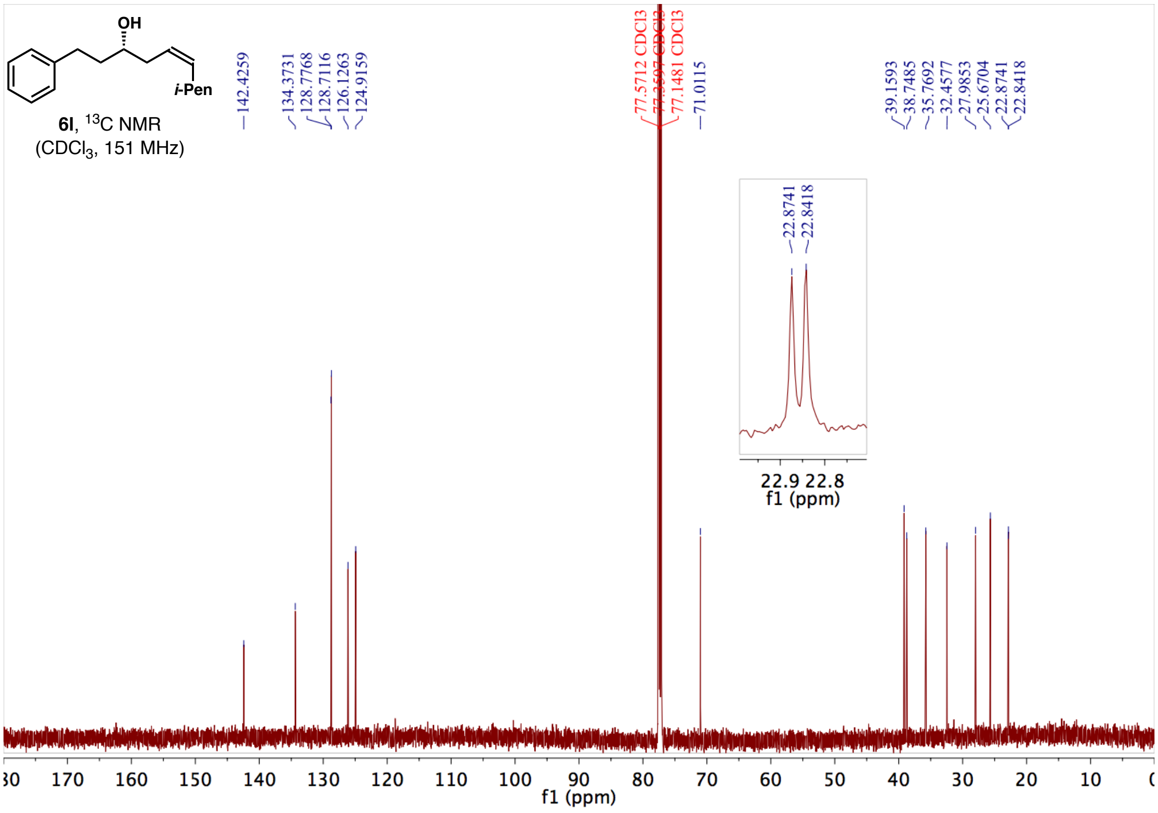

OH
i-Pen
6l, 13C NMR
(CDCl3, 151 MHz)
142.4259
134.3731
128.7768
128.7116
126.1263
124.9159
77.5712 CDCl3
77.1481 CDCl3
71.0115
39.1593
38.7485
35.7692
32.4577
27.9853
25.6704
22.8741
22.8418
f1 (ppm)

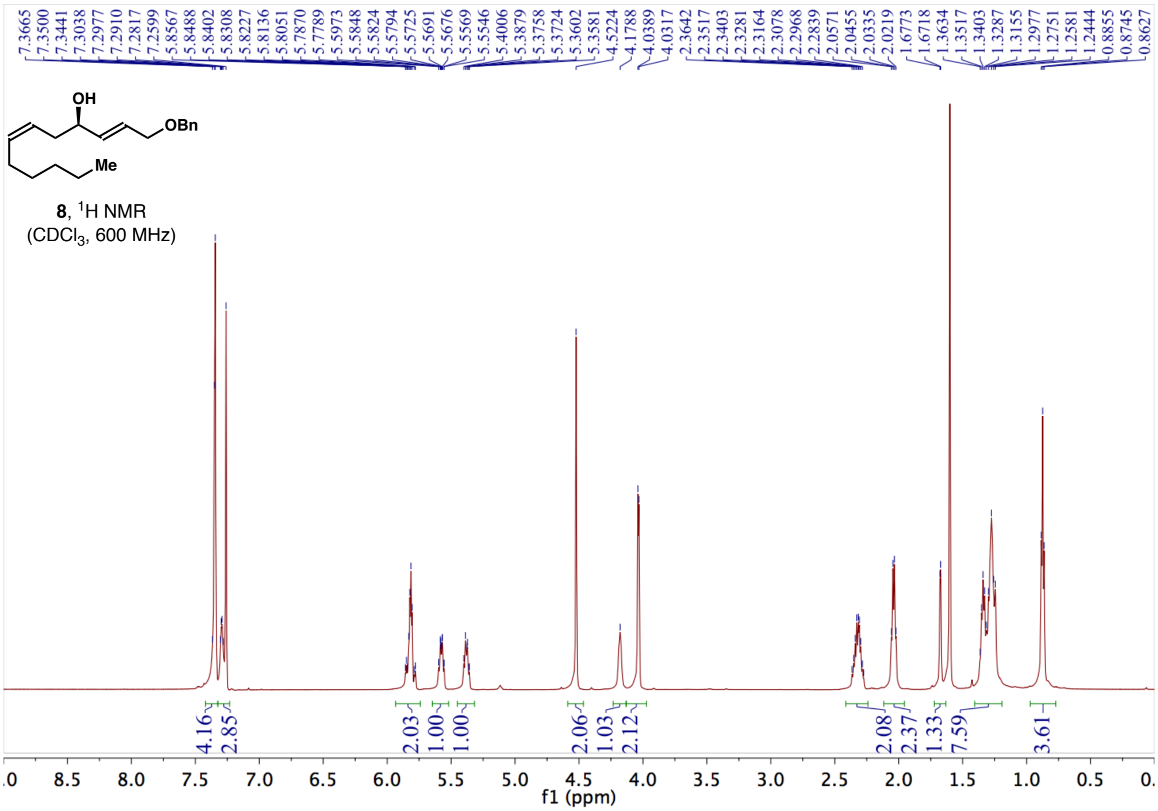
7.3665 7.3500 7.3441 7.3038 7.2977 7.2910 7.2817 7.2599 5.8567 5.8488 5.8402 5.8308 5.8227 5.8136 5.8051 5.7870 5.7789 5.5973 5.5848 5.5824 5.5794 5.5725 5.5691 5.5676 5.5569 5.5546 5.4006 5.3879 5.3758 5.3724 5.3602 5.3581 4.5224 4.1788 4.0389 4.0317 2.3642 2.3517 2.3403 2.3281 2.3164 2.3078 2.2968 2.2839 2.0571 2.0455 2.0335 2.0219 1.6773 1.6718 1.3634 1.3517 1.3403 1.3287 1.3155 1.2977 1.2751 1.2581 1.2444 0.8855 0.8745 0.8627
OH
OBn
Me
8, ^{1}H NMR
(CDCl$_3$, 600 MHz)
4.16 2.85 2.03 1.00 1.00 2.06 1.03 2.12 2.08 2.37 1.33 7.59 3.61
f1 (ppm)

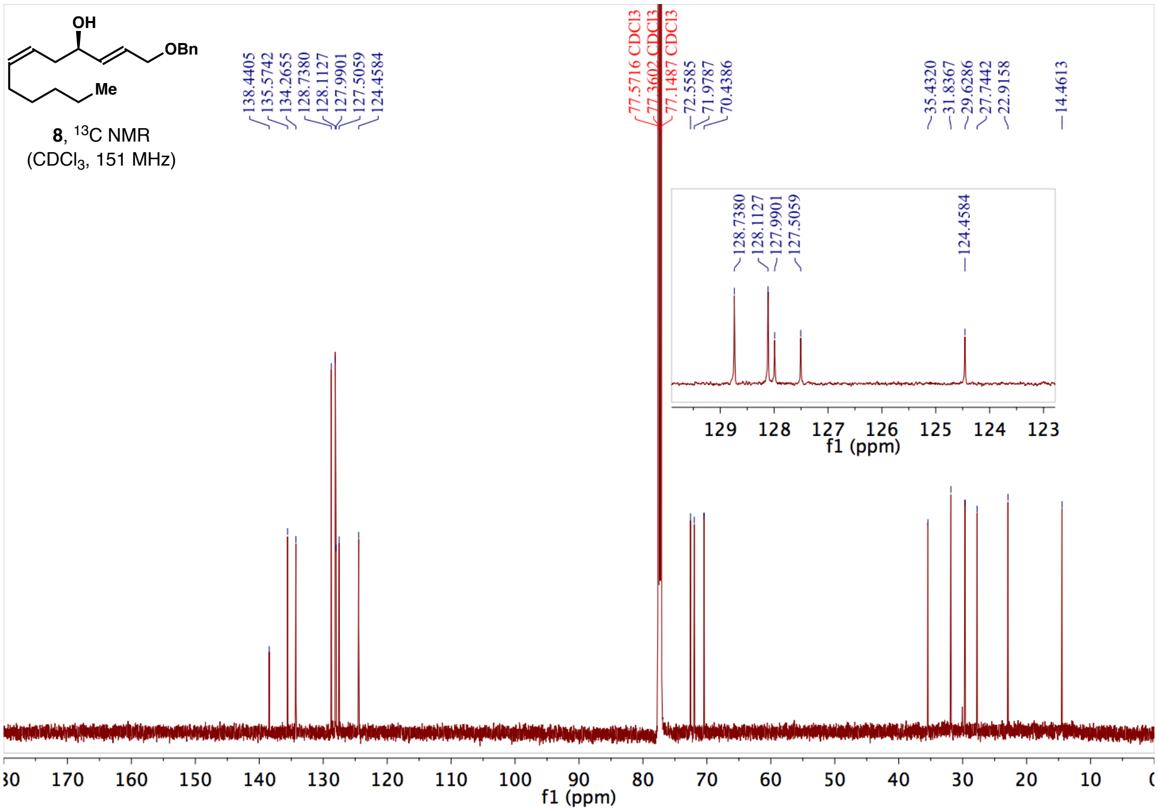
OH
OBn
Me
8, ^{13}C NMR
(CDCl$_3$, 151 MHz)
138.4405 135.5742 134.2655 128.7380 128.1127 127.9901 127.5059 124.4584
77.5716 CDCl3 77.3602 CDCl3 77.1487 CDCl3
72.5585 71.9787 70.4386
35.4320 31.8367 29.6286 27.7442 22.9158
14.4613
f1 (ppm)

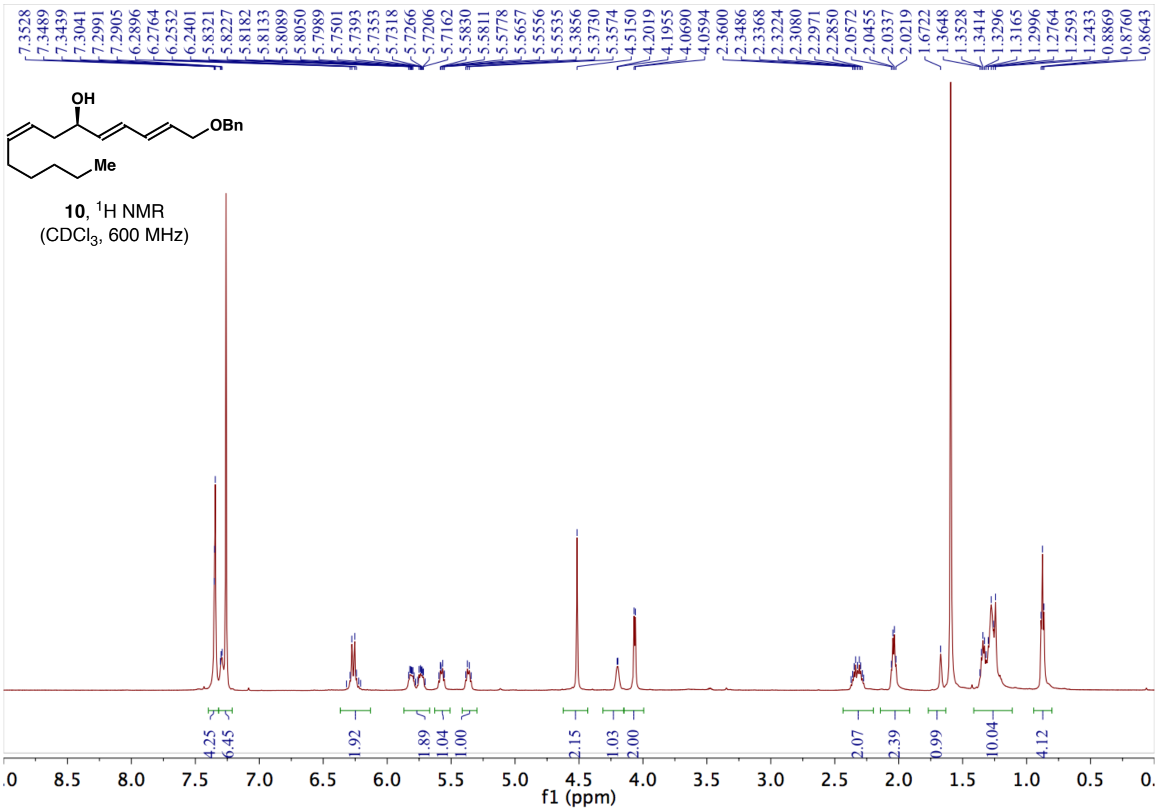

OH
OBn
Me
10, ¹H NMR
(CDCl3, 600 MHz)
f1 (ppm)

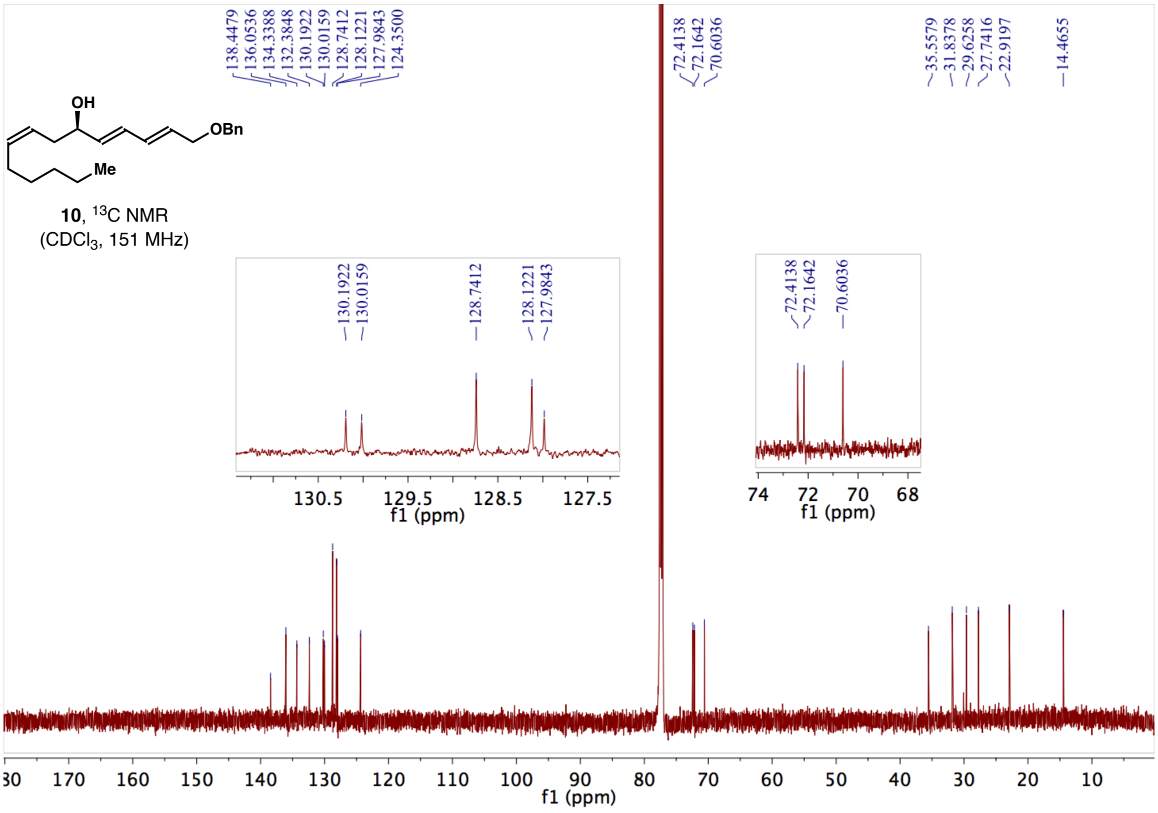

OH
OBn
Me
10, ¹³C NMR
(CDCl3, 151 MHz)
f1 (ppm)
